# In situ visualization of Braun’s lipoprotein on *E. coli* sacculi

**DOI:** 10.1126/sciadv.add8659

**Published:** 2023-01-20

**Authors:** Qi Sheng, Meng-Yao Zhang, Si-Min Liu, Zhuo-Wei Chen, Pei-Ling Yang, Hong-Su Zhang, Meng-Yun Liu, Kang Li, Long-Sheng Zhao, Ning-Hua Liu, Lu-Ning Liu, Xiu-Lan Chen, Jamie K. Hobbs, Simon J. Foster, Yu-Zhong Zhang, Hai-Nan Su

**Affiliations:** ^1^ State Key Laboratory of Microbial Technology, Shandong University, Qingdao 266237, China.; ^2^ College of Marine Life Sciences and Frontiers Science Center for Deep Ocean Multispheres and Earth System, Ocean University of China, Qingdao 266003, China.; ^3^ Laboratory for Marine Biology and Biotechnology, Pilot National Laboratory for Marine Science and Technology, Qingdao 266237, China.; ^4^ Institute of Systems, Molecular and Integrative Biology, University of Liverpool, Liverpool, UK.; ^5^ The Florey Institute for Host-Pathogen Interactions, University of Sheffield, Sheffield, UK.; ^6^ Department of Physics and Astronomy, University of Sheffield, Sheffield, UK.; ^7^ School of Biosciences, University of Sheffield, Sheffield, UK.; ^8^ Marine Biotechnology Research Center, State Key Laboratory of Microbial Technology, Shandong University, Qingdao 266237, China.

## Abstract

Braun’s lipoprotein (Lpp) plays a major role in stabilizing the integrity of the cell envelope in *Escherichia coli*, as it provides a covalent cross-link between the outer membrane and the peptidoglycan layer. An important challenge in elucidating the physiological role of Lpp lies in attaining a detailed understanding of its distribution on the peptidoglycan layer. Here, using atomic force microscopy, we visualized Lpp directly on peptidoglycan sacculi. Lpp is homogeneously distributed over the outer surface of the sacculus at a high density. However, it is absent at the constriction site during cell division, revealing its role in the cell division process with Pal, another cell envelope–associated protein. Collectively, we have established a framework to elucidate the distribution of Lpp and other peptidoglycan-bound proteins via a direct imaging modality.

## Teaser

Direct visualization of the most abundant protein in *E. coli* revealed a previously unknown mechanism in cell division process.

## INTRODUCTION

The envelope of Gram-negative bacteria is composed of three layers: the outer membrane, peptidoglycan sacculus, and inner membrane. The existence of the outer membrane allows the formation of the periplasm, which provides a critical cellular compartment for diverse processes such as protein folding and quality control, environmental sensing, signal transduction, regulation of cell growth and division, assembly of periplasmic protein complexes, etc. (*1*–*5*). To ensure a functional periplasm, Gram-negative bacteria must maintain the outer membrane. A series of proteins are known to participate in outer membrane stability, such as Braun’s lipoprotein (Lpp), peptidoglycan-associated lipoprotein (Pal), and outer membrane protein A (OmpA) (*6*–*8*). Among these, Lpp plays an important role in maintaining cellular architecture in *Escherichia coli* as it provides the only known covalent connection between the outer membrane and the peptidoglycan layer (*9*–*11*).

Lpp (a.k.a. major outer membrane lipoprotein or murein lipoprotein) is a small lipoprotein that is composed of a 5.8-kDa protein moiety and three lipid molecules (*8*). The lipid part is inserted into the outer membrane while the C terminus of the protein is covalently linked to the peptidoglycan layer (*8*). Numerous Lpp molecules support the outer membrane and, as a result, stabilize the periplasm. The role of Lpp is so important that a large pool of Lpp molecules must be maintained in a bacterial cell. In *E. coli*, Lpp is the most abundant protein by copy number, where the number of molecules has been estimated to be several hundred thousand to a million copies per cell (*12*). Loss of Lpp leads to instability of the outer membrane, formation of outer membrane blebs, and leakage of periplasmic proteins (*13*). Moreover, it was suggested that Lpp determines the width of the periplasm. Periplasmic width increases proportionally to the length of Lpp (*10*), leading to changes in signal transduction across the periplasm (*10*), the mechanical properties of the cell envelope (*9*), and structural alterations to protein complexes in the periplasm such as the flagellar motor (*11*).

Because Lpp supports the outer membrane and controls the distance between the outer membrane and the peptidoglycan layer, elucidating its distribution across the peptidoglycan sacculus is important to give an understanding of the basic structural arrangement of the cell envelope, as well as those cellular processes occurring in the periplasm. Lpp is thought to covalently bound to every 10th repeating unit of the murein (*14*); therefore, Lpp should be densely distributed on the peptidoglycan layer. However, it is not known whether Lpp is homogeneously distributed or arranged in a particular pattern, especially in terms of cell morphology and dynamics. The polar regions of the peptidoglycan sacculus differ from the cylinder in that they are metabolically inert (*15*). In addition, outer membrane proteins, including Pal, that are associated with the peptidoglycan are involved in outer membrane constriction at the division site (*4*, *16*).

Although Lpp has been studied for more than half a century (*14*), many mysteries concerning this small lipoprotein still remain to be addressed. One of the key reasons is a lack of detailed knowledge of Lpp distribution on the sacculus. To map Lpp, a direct observation of its organization on the peptidoglycan layer is required. However, it is difficult for traditional high-resolution microscopic methods such as transmission electron microscopy to directly distinguish Lpp on peptidoglycan, in part due to the diminutive protein size. Here, we have successfully achieved direct observation of Lpp at high resolution using atomic force microscopy (AFM) and revealed the distribution characteristics of Lpp on the sacculus of *E. coli* and other bacterial species. Lpp subcellular distribution has been established, and a role of Lpp in cell division has been revealed.

## RESULTS

### AFM permits direct visualization of Lpp on the *E. coli* sacculus

**Fig. 1.**
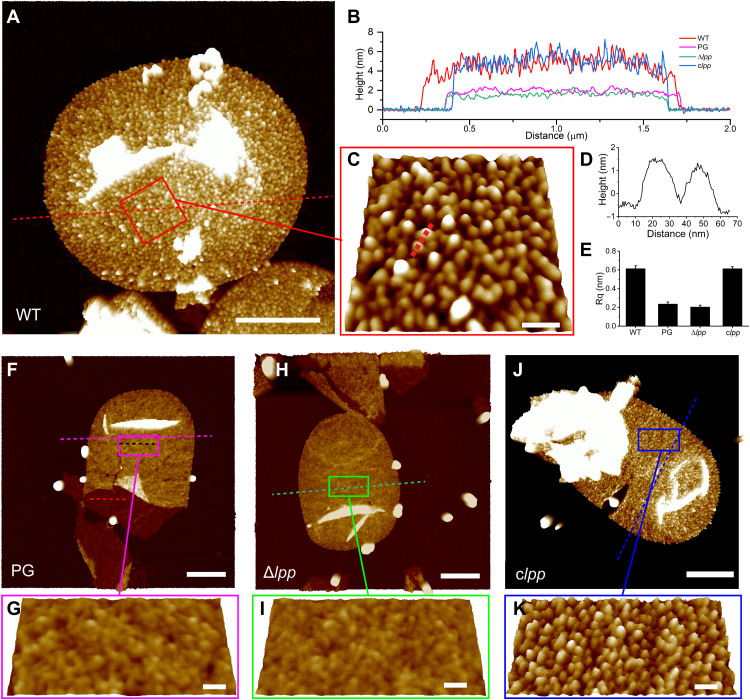
Visualization of protein particles on *E. coli* sacculi with AFM. The dense particle-like structures on the sacculus surface under AFM are identified as Lpp. (**A**) sacculus from WT *E. coli* demonstrating the presence of surface particles. (**B**) Height profiles of sacculi from (A), (F), (H), and (J). Dashed lines indicate positions for sectional analysis. (**C**) Higher-magnification image of the boxed region in (A). (**D**) Height profile of sacculus from (C) corresponding to the dashed line. (**E**) Surface roughness analysis of sacculi. (**F**) Sacculus from which proteinaceous material has been removed by trypsin. PG, peptidoglycan. (**G**) Higher-resolution image of the boxed region in (F). (**H**) Sacculus of Δ*lpp* strain. (**I**) Higher-resolution image of the boxed region in (H). (**J**) Sacculus of the *lpp*-complemented strain. (**K**) Higher-resolution image of the boxed region in (J). For (A), (F), (H), and (J): scale bars, 500 nm; height scale, 8 nm. For (C), (G), (I), and (K): pitch, 25°; scale bars, 50 nm; height scale, 3.5 nm.

Sacculus purification for AFM analysis usually contains a protease step to remove ionically bound proteins, those remaining after detergent extraction, and any proteins covalently attached to the peptidoglycan (*17*, *18*). To investigate proteins covalently bound to the sacculus of *E. coli*, the protease treatment was omitted. Isolated sacculi were then investigated by AFM. In contrast to previous AFM observations of sacculi after protease treatment, we were surprised to find numerous small particle-like structures that are densely distributed over the entire surface of the sacculus (Fig. 1A). The average thickness of the sacculus with particle-like structures was 4.9 ± 0.5 nm (*n* = 10; Fig. 1B). The sizes of the small particles are rather homogeneous (Fig. 1C), with a diameter of 17.7 ± 1.9 nm (*n* = 50; Fig. 1D).

The observed, sacculus-associated particles are sensitive to protease treatment (fig. S1), implying that they are of proteinaceous nature. After trypsin treatment, the particle-like structures were completely removed from the sacculi (Fig. 1, F and G). The thickness of sacculi was reduced to 2.1 ± 0.2 nm (*n* = 10; Fig. 1B). As the particle-like structures were removed, the surface roughness of sacculi reduced from 0.6 to 0.2 (Fig. 1E). The morphology of the sacculus after trypsin treatment was in accordance with previous observations (*17*), revealing that the particles are not an artifact of the purification procedure but are due to the omission of the protease treatment. After digestion with trypsin, the resulting solubilized peptide fragments were analyzed by liquid chromatography coupled with tandem mass spectrometry (LC-MS/MS). By far, the most predominant protein was predicted to be the major outer membrane lipoprotein Lpp (fig. S2 and table S1).

To verify whether the sacculus-associated particles are Lpp, a corresponding deletion mutant (Δ*lpp*) was used. Deletion of *lpp* led to the formation of outer membrane blebs, as expected (fig. S3). AFM of purified sacculi, without trypsin treatment, revealed that the sacculus surface was not studded with particles (Fig. 1, H and I). Moreover, the morphology, thickness, and surface roughness of the sacculus from Δ*lpp* resembled those of the sacculus from the wild-type (WT) strain, when treated with trypsin (Fig. 1, B and E). Complementation of *lpp* in the Δ*lpp* background led to the reappearance of the particle-like structures on sacculi (Fig. 1, J and K). The sacculi from the *lpp*-complemented strain also had comparable thickness and roughness to that of the parental strain. *E. coli* encodes six l,d-transpeptidases, of which three (LdtA, LdtB, and LdtC) are collectively responsible for covalently attaching Lpp onto peptidoglycan (*19*). When all these three Ldts were removed from *E. coli*, sacculus-associated particles disappeared (fig. S4).

It can thus be concluded that the proteinaceous particles on the sacculi comprise Lpp. We were unable to visualize the particle-like structures using transmission electron microscopy (fig. S5), likely due to the lack of contrast using this approach.

### Lpp is distributed exclusively on the outer surface of the sacculus

**Fig. 2.**
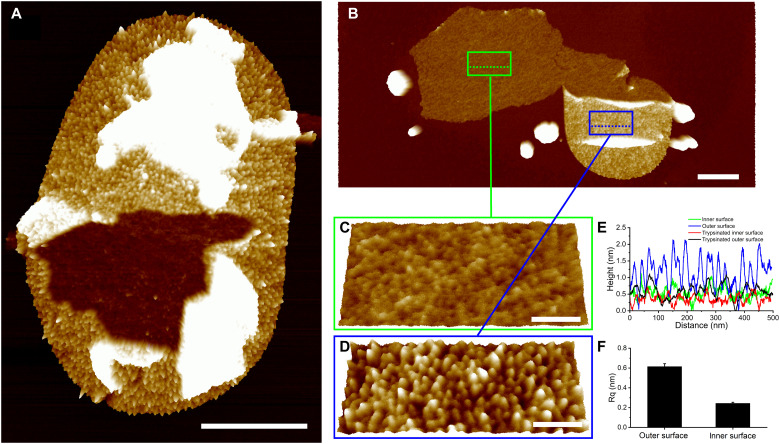
Lpp is localized on the sacculus outer surface. (**A** and **B**) Broken sacculi with both inner and outer surfaces exposed. (**C**) Inner surface, green box; (**D**) outer surface, blue box; higher-resolution images of the boxed regions in (B). (**E**) Height profiles of sacculi from (C) and (D) and trypsinated sacculi, normalized to the minimum value. Dotted lines in (B) and Fig. 1F indicate the position for sectional analysis. (**F**) Roughness analysis of the outer (D) and inner (C) surfaces of sacculi. Rq was measured on a size of 200 nm by 200 nm (*n* = 6). For (A) and (B): scale bars, 500 nm; height scale, 8 nm. For (C) and (D): pitch, 25°; scale bars, 100 nm; height scale, 3.5 nm.

Gently broken sacculi of *E. coli* were analyzed by AFM in which the inner and outer surfaces of the sacculi were clearly differentiated (Fig. 2A). The particles of Lpp are distributed exclusively on the outer surface of the sacculi (Fig. 2B). Unlike the outer surface, the inner surface of the sacculus is seemingly devoid of Lpp (Fig. 2C), with only occasional Lpp-like structures observed (Fig. 2A). Sectional analysis revealed the inner sacculus surface to be relatively smooth compared to the outer surface (Fig. 2E). The inner surface roughness (Fig. 2F) is comparable to the outer surface when Lpp is removed (Fig. 1E). This observation supports previous biochemical analysis, which proposed that Lpp is located on the outer surface of sacculus (*20*), and provides the first direct evidence for such surface specificity. Appropriate Lpp localization is crucial for *E. coli* as covalent linkage between mislocalized Lpp and the peptidoglycan is fatal (*21*).

As Lpp plays crucial roles in a range of bacterial cellular processes (*8*), it is important to ascertain its temporospatial distribution during growth and division. Sacculi from *E. coli* grown to mid-exponential and stationary phase were isolated and observed by AFM. Initially, the sacculi that were not observed to be septating were analyzed to determine the distribution of Lpp (fig. S6, A and B). In sacculi from *E. coli* at the mid-exponential phase, the density of particle-like structures was about 1100/μm^2^ both at the mid-cell and pole area (*n* = 10), while in sacculi from stationary-phase cells, the density at the mid-cell and pole increased for both to about 1300/μm^2^ (*n* = 10; fig. S6C). Statistical analysis suggested that this does not constitute a significant difference (*P* > 0.1) in Lpp distribution along the cell axis in nondividing cells at either growth phase. The sacculus surface roughness also equated to the particle distribution (fig. S6D), with no significant statistical difference among the cells at either growth phase (*P* > 0.1).

### Lpp is not recruited to the septation site during cell division

**Fig. 3.**
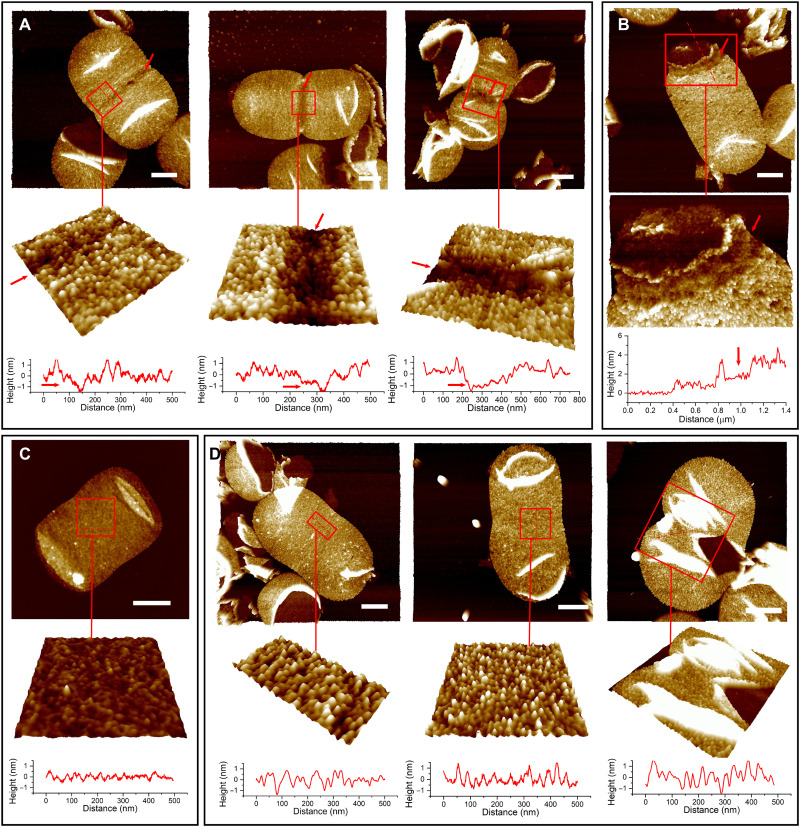
Lpp is absent at the constriction site during cell division. (**A**) A groove-like Lpp-free region is present on sacculi at different stages of cell division. Scale bars, 500 nm. Height scale is 3.5 nm for the left two enlarged images and 5 nm for the right one. Arrow indicates the Lpp-free region. (**B**) A broken sacculus showing the groove at the constriction site. Scale bar, 500 nm. Height scale is 6 nm for the enlarged image. (**C**) Sacculus with Lpp removed by trypsin treatment, showing that the groove-like structure has disappeared. Scale bar, 500 nm. Height scale is 3.5 nm for the enlarged image. (**D**) Sacculi from Δ*pal* at different stages of cell division. Scale bars, 500 nm. Height scale is 3.5 nm for the left two enlarged images and 12 nm for the right one. Images below in each panel show higher-magnification images of the division region presented in pseudo-3D to more clearly visualize the height variations across the area. For the enlarged images in (A), (C), and (D), pitch is 45°. Squares in (A) to (D) indicate the position for enlargement. Height profiles of sacculi are shown at the bottom of the enlarged image. Dashed lines indicate the position for their section analysis.

Sacculi from exponentially growing *E. coli* were analyzed, concentrating on cells that demonstrated the division-associated constriction across the short axis. In these sacculi, a groove-like area that formed by the absence of Lpp particles was present at the constriction site (Fig. 3A). The groove structure was present at different stages of cell division (Fig. 3A), indicating the lack of Lpp incorporation during the division process. Sacculi that represent late stages of division usually have folds, especially at constriction sites (fig. S7), presumably due to the hourglass-shaped geometry of the dividing cell. The groove was more visible in broken sacculi that kept the constriction site (Fig. 3B). Moreover, the groove disappeared when Lpp was removed by trypsin digestion (Fig. 3C). The width of the groove was mostly 40 to 80 nm at different stages of cell division (fig. S8), implying that Lpp was back-filled into the groove along with the constriction process. A narrower groove was only observed occasionally in sacculi likely initiating cell division (fig. S9).

The unexpected division-associated lack of Lpp incorporation at the developing septum was intriguing. Thus, to begin to determine how this might occur and its role, we analyzed a Δ*pal* strain. Pal is a lipoprotein that interacts with peptidoglycan via a noncovalent interaction. It has previously been shown to be recruited to the constriction site during cell division (*22*). The *pal* mutant can grow normally under laboratory conditions (fig. S10). Unexpectedly, the groove structure at the division site disappeared in Δ*pal* (*n* = 13, with Lc/Ls ratios of 0.69 to 0.96. Lc, width of sacculi at the constriction site. Ls, maximum width of sacculi perpendicular to the cell longaxis) (Fig. 3D). This indicates that the presence of Pal prevents Lpp incorporation into the sacculus during division itself, but this is not an essential process as Δ*pal* does not prevent cell division.

### Loss of Pal and Lpp results in a defect in outer membrane invagination

**Fig. 4.**
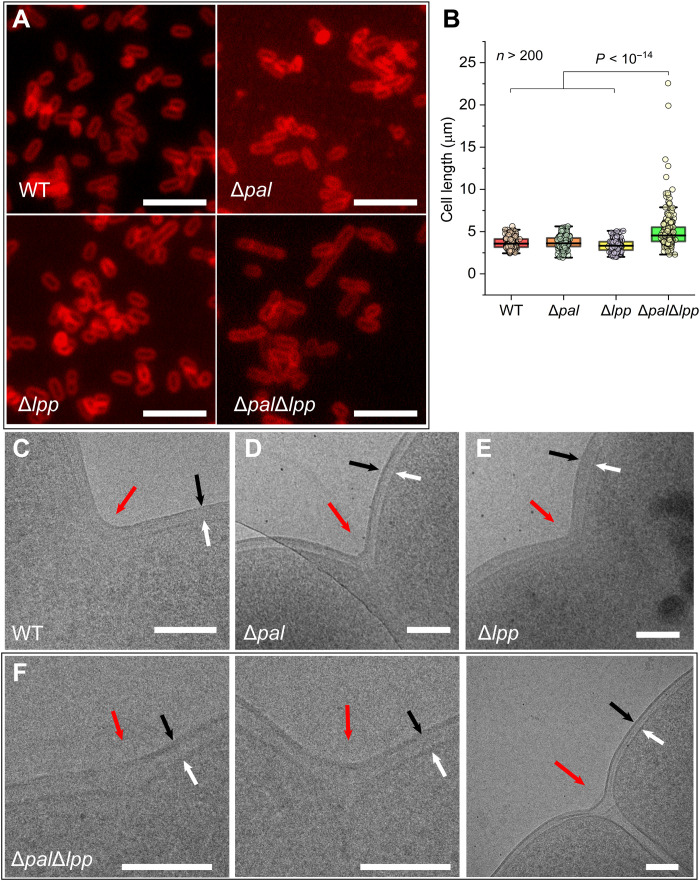
Loss of Pal and Lpp leads to an outer membrane invagination defect. (**A**) Fluorescence microscope observations on WT *E. coli* and Δ*pal*, Δ*lpp*, and Δ*pal*Δ*lpp* mutants. Cells grown in LB medium were collected at the mid-exponential phase and stained with FMTM 4-64. Scale bars, 10 μm. (**B**) Dot distribution and box plots reporting the length of each bacterial strain estimated from fluorescence microscopic observation. Shown here are the median, the 25 and 75% quartiles (boxes), and the SD (whiskers). *n* > 200 cells for each strain. (**C** to **F**) Cryo–electron microscopic observation on dividing *E. coli*, showing the effects of the loss of Pal/Lpp on outer membrane invagination. Red arrow indicates the septa. Black arrow indicates the outer membrane. White arrow indicates the inner membrane. Scale bars, 100 nm.

Pal participates in outer membrane invagination during cell division in *E. coli* (*16*, *22*). The *pal* mutant forms filamentous cells when cultured in LB growth medium without NaCl but grows normally in standard LB medium (*22*). However, short cell chains are formed in a double Δ*pal*Δ*lpp* strain when cultured in standard LB (Fig. 4, A and B), indicating that at least one of these two proteins is required for proper cell division in *E. coli*. The Δ*pal*Δ*lpp* strain does not survive in LB growth medium [0% (w/v) NaCl] (fig. S10), implying a severe impact on *E. coli* when both Pal and Lpp are lost.

The cell envelope architecture of the strains was then determined using cryo–electron microscopy. In WT *E. coli*, there is a coordination of outer membrane and inner membrane invagination at the septum during cell division (Fig. 4C). This coordination is mostly maintained if either *pal* or *lpp* is deleted (Fig. 4, D and E). However, in the Δ*pal*Δ*lpp* strain, impaired coordination of the membrane layers is apparent. Although the inner membrane can invaginate at the septal site, the outer membrane cannot, aside from a slight constriction (Fig. 4F). This results in the observed filamentation phenotype for the Δ*pal*Δ*lpp* strain.

### Protein display on sacculi is conserved in other enterobacterial species

**Fig. 5.**
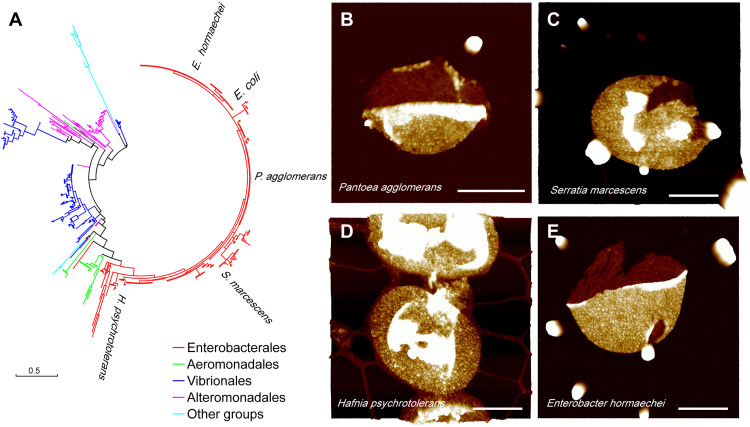
Dense protein distribution on the outer surface of sacculi from enterobacterial species. (**A**) Phylogenetic analysis of Lpp amino acid sequences inferred by using the maximum likelihood method with MEGA7. The tree is drawn to scale with branch lengths measured in the number of substitutions per site. AFM observations on sacculi from (**B**) *P. agglomerans*, (**C**) *S. marcescens*, (**D**) *Hafnia psychrotolerans*, and (**E**) *E. hormaechei*. Scale bars, 500 nm. Height scale, 8 nm.

Sequence analysis indicates that genes homologous to lpp are widely distributed in Enterobacteriales and exist in some other groups of Gammaproteobacteria, such as Vibrionales, Alteromonadales, and Aeromonadales (Fig. 5A), suggesting the importance of Lpp in Gammaproteobacteria. To determine whether the sacculus display of Lpp is conserved across species, we analyzed the sacculi from several other species of Enterobacteriales (Fig. 5, B to E). Small particle-like structures that resemble Lpp from *E. coli* are densely distributed on the entire surface of the sacculi in all species tested. Broken sacculi also revealed that the Lpp-like structures exist only on the sacculus outer surface.

## DISCUSSION

Lpp was first described in *E. coli* by Braun and Rehn in 1969 (*14*). It has covalently linked lipid molecules at the N-terminal cysteine residue, which help it to attach to the outer membrane. The C terminus of Lpp has a lysine residue that is covalently cross-linked to the peptidoglycan layer. Lpp is widely conserved across Gammaproteobacteria, and it was the only known protein that can be covalently linked to peptidoglycan in Gram-negative bacteria until 2021, when several β-barrel proteins covalently bound to peptidoglycan were identified in Alphaproteobacteria (*23*, *24*). As the most abundant protein in *E. coli*, Lpp stabilizes the outer membrane and the periplasm, providing an architecturally distinct cellular compartment for bacterial physiological processes. Since its discovery, the role of Lpp and its potential biotechnological application have been the subject of extensive studies (*8*). However, the lack of information about the distribution of Lpp on sacculi prevents a deeper understanding of the function of Lpp, as well as those cellular processes that occur in and across the periplasm.

Using a transmission electron microscopy approach, it is difficult to directly distinguish Lpp on sacculi. One reason may be that Lpp is a very small α-helical protein, with only 58 amino acid residues in its mature form, and it could be hidden in the background of the sacculus. Immunoelectron microscopy has given an indirect measure of Lpp distribution (*25*). Here, we have successfully achieved direct observation of Lpp on sacculi using high-resolution AFM. By reconstructing the signal from the AFM scanning probe, a high-resolution image of sample surface properties is obtained. AFM has been widely used in research on biological macromolecules on intact cells (*26*–*29*). Although Lpp is only a small protein, it can be readily detected by the sensitive AFM probe.

Previous estimates showed that the amount of Lpp molecules in *E. coli* is about 3 × 10^5^ to 7 × 10^5^ per cell (*25*, *30*) or more than 1 × 10^6^ per generation (*12*). It has been suggested that Lpp forms trimers in cells (*31*). In addition, only about one-third of Lpp molecules are covalently bound to peptidoglycan, while the rest are in the free form (*32*). Thus, an estimate of several tens of thousands of Lpp trimers per sacculus can be made. However, in our work, the number of particle structures on sacculi, as measured by AFM, attributable to Lpp was roughly estimated to be several thousands. This is an order of magnitude less than the theoretical amount of cross-linked Lpp trimers per sacculus. A possible explanation for the discrepancy could be that each AFM-observed particle is formed of a cluster of Lpp trimers. The sizes of the particle structures observed by AFM are rather homogeneous, but the diameter of each particle (17.7 ± 1.9 nm) is much larger than that of a theoretical Lpp trimer (~2 nm), which may support this hypothesis.

We observed that Lpp distributes almost exclusively on the outer surface of the sacculus, which corroborates its role in attaching the outer membrane to the peptidoglycan. When the Lpp outer membrane sorting signal is altered or the transport pathway is blocked, Lpp accumulates at the inner membrane (*21*, *33*). Covalent linkage of Lpp located in the inner membrane to peptidoglycan leads to cell death (*21*). Although the cellular localization of Lpp has long been suggested by chemical and genetical analysis, here, we provide the first direct observational evidence that Lpp cross-linked to peptidoglycan is distributed exclusively facing toward the outer membrane.

Even given the high abundance of Lpp molecules per cell, it is still intriguing to observe the dense packing of the protein on the sacculus surface, leaving barely any uncovered space. The dense packing is likely necessary to provide an even support structure for the outer membrane. However, it brings about an interesting question as to how Lpp is juxtaposed to other periplasmic proteins or protein complexes that perform important cellular functions. The AFM probe tip can lead to a sample broadening effect (*34*–*36*), and so, the Lpp cluster is most likely smaller than that measured. Aggregation of lipids in adjacent Lpp may also contribute to the formation of Lpp clusters.

In the cells that are not dividing, Lpp shows an apparent homogeneous distribution along the cell cylinder and pole, alluding to its required function to support the outer membrane around the entire cell periphery. However, an interesting discovery in our work is the heterogeneous distribution of Lpp in dividing cells. It is critical for an *E. coli* cell to coordinate outer membrane constriction with septation during cell division to ensure the maintenance of cellular integrity (*4*). Outer membrane proteins that tether the peptidoglycan layer, either by covalent linkage or by noncovalent interaction, are potential candidates that may be involved in this process (*4*). However, the role and dynamics of Lpp during cell division are unknown, especially as an lpp mutant can grow normally under laboratory conditions (*37*, *38*). We observed that, in dividing cells, Lpp is not present at the division site, whereby sacculi have a groove-like structure devoid of Lpp. The formation of such a groove-like structure may result from a mutually exclusive functional interaction with other proteins at the division site. Pal is a lipoprotein that can interact with peptidoglycan via noncovalent interaction. It is known to be recruited to the constriction site during cell division, and accumulation of Pal at the septum supports the invagination of the outer membrane during division (*16*, *22*). It was thus interesting to find that a pal strain displayed Lpp across the entire sacculus during division, without the groove-like structure. This supports the hypothesis that Pal may prevent Lpp cross-linking at the septal region during cell division.

**Fig. 6.**
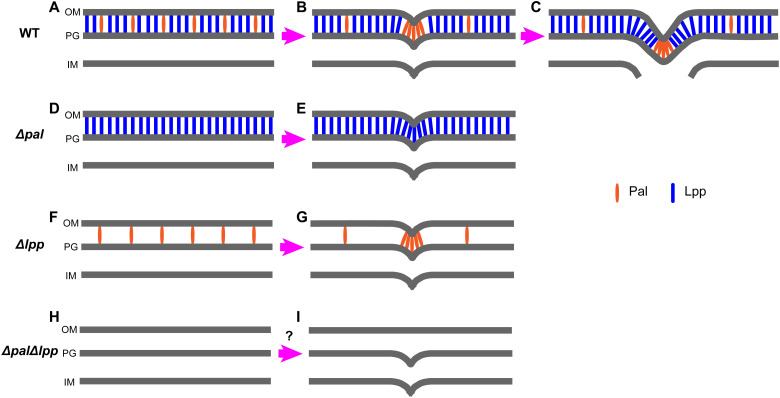
Model for the role of Lpp and Pal in outer membrane constriction during division. (**A**) In a nondividing cell, Lpp distributes homogeneously over the surface of the sacculus in WT *E. coli* (Fig. 1). Pal is distributed homogeneously over the surface of sacculus (*16*) with fewer molecules than Lpp (*12*, *30*, *41*). (**B**) After Pal is recruited to the constriction site, outer membrane constriction initiates (*16*, *22*). Lpp is not present at the constriction site (Fig. 3A). (**C**) Pal is aggregated at the leading edge of the septation site until division completes, while Lpp is linked to the sacculus except at the leading edge (Fig. 3A). (**D**) When Pal is absent, Lpp distributes over the entire surface of the sacculus. (**E**) In the *pal* mutant, Lpp occupies the constriction site permitting cell division (Fig. 3D). (**F**) In the *lpp* mutant, the density of Pal on the sacculus does not change much compared with WT *E. coli* (*41*). (**G**) The *lpp* mutant can grow and divide like WT *E. coli*. (**H**) In the *pal* and *lpp* double mutant, no Pal or Lpp is present. (**I**) During division, the lack of both proteins results in a defect in outer membrane invagination (Fig. 4F). OM, outer membrane. IM, inner membrane.

Such a mutually exclusive interaction between Pal and Lpp is reasonable, because both of them interact with the meso-diaminopimelate (m-DAP) residue in the peptide stems of peptidoglycan (*6*). The difference lies in the fact that Pal interacts with m-DAP in a noncovalent form (*39*), while Lpp is covalently linked to m-DAP (*40*). The *pal* or *lpp* mutant usually forms outer membrane blebbing (*41*), and in LB medium lacking NaCl, the *pal* mutant grows into chains (*22*, *42*, *43*). However, loss of individual Pal or Lpp does not prevent cell division of *E. coli* when cultured in standard LB growth medium. However, the Δ*pal*Δ*lpp* strain usually forms cell chains in standard LB medium. This indicates that Pal and Lpp are important for cell division, whereby the presence of either is sufficient for outer membrane invagination. This allows a simple model to be derived whereby the Pal and Lpp proteins provide a mutually compensatory mechanism to support cell division (Fig. 6).

Although the loss of both Pal and Lpp led to defects in outer membrane invagination, daughter cell separation is still present in the Δ*pal*Δ*lpp* mutant, where both cell chains and short cells can be observed. Other factors could be present to overcome the absence of Pal and Lpp, which is worthy to be explored in future research.

AFM has provided the first direct evidence of the distribution pattern of Lpp on the *E. coli* sacculus, a display that is conserved across disparate bacterial species. These initial discoveries now provide access to be able to understand the molecular mechanisms, at the nanoscale, that underpin basic cellular architecture and the maintenance of cellular processes that permit growth and division.

## MATERIALS AND METHODS

### Bacterial strains and growth conditions

Bacterial species used in this study are shown in table S2. All *E. coli* mutant strains were derived from the MG1655 background. Bacterial strains were cultured using LB. Cells from a single colony taken from an agar plate were placed in LB and grown at 37°C overnight with constant agitation. These cells were then resuspended in fresh LB to OD_600_ (optical density at 600 nm) = 0.05 to 0.1 and grown to exponential phase or stationary phase.

A series of *E. coli* mutants with target gene deletions were constructed from the WT MG1655 strain using a λ Red–mediated recombination system. Briefly, polymerase chain reaction (PCR) products were generated from template plasmids carrying a kanamycin-resistant (K^+^) gene flanked by flippase (FLP) recognition target sites using primers with 50-nucleotide extensions that were homologous to regions adjacent to the target genes. The chromosomal genes were replaced by the corresponding PCR products through the λ Red–mediated recombination system. The resulting K^+^ colonies were selected and verified by PCR, and the kanamycin-resistant cassette was removed by introducing a pCP20 helper plasmid that carried the Flp recombinase and ampicillin-resistant gene. The Red and FLP helper plasmids were subsequently cured by growth at 37°C as they are temperature sensitive. All primers used in this study are listed in table S3.

### Purification of sacculi

Sacculi were purified as described previously (*17*, *18*) with modifications. Cells were collected by centrifugation (3000*g*, 5 min) and washed three times with distilled water. Cells were then collected and resuspended in distilled water and broken by a high-pressure cell disrupter (Constant Systems Ltd). The suspension was centrifuged (3000*g*, 5 min), and the pellet was discarded. The collected suspension was then centrifuged at 20,800*g* for 60 min, and the pellet was collected. The collected pellet was resuspended in 5% (w/v) SDS in distilled water and boiled for 60 min. The resulting mixture was washed with Milli-Q water by ultracentrifugation (156,000*g*, 20 min) for at least three times. The collected pellet was suspended in Milli-Q water for AFM imaging or suspended in sodium phosphate buffer (10 mM, pH 7.4) for proteomic analysis.

### AFM imaging

A drop (1.5 μl) of sacculi suspension was spread onto freshly cleaved mica and air-dried. AFM imaging was carried out using a Multimode VIII AFM with a Nanoscope V controller (Bruker, USA). Silicon cantilevers (XSC11/AI BS, MikroMasch) were used for imaging under ambient conditions. AFM imaging was carried out in ScanAsyst mode in air condition.

### AFM image analysis

All displayed AFM images are three-dimensional (3D) height images. The z aspect ratio of all AFM images is 0.1. Section analysis and surface roughness analysis were carried out using NanoScope Analysis v1.40 software.

### Proteomic analysis

Sacculi were digested with trypsin (final concentration of 2 mg/ml) at 37°C with continuous stirring for at least 12 hours, and the reaction was stopped by the addition of 1% (v/v) trifluoroacetic acid. The solution was incubated at 37°C for 15 min and then centrifuged at 92,000*g* for 30 min. The supernatant was collected and dried in a vacuum concentrator. The sample was suspended in Milli-Q water. Peptides were analyzed by LC-MS/MS on an Orbitrap Fusion Lumos mass spectrometer. The raw files were processed with Proteome Discoverer version 2.3 to generate a list of identified proteins. Protein identifications were accepted if they contained at least two identified peptides. Subcellular localization determination for the identified proteins was performed using PSORTb version 3.0.3 (*44*).

### Fluorescence microscope imaging

*E. coli* strains were imaged as described previously (*45*). Bacterial cells from mid-log phase were collected and resuspended in 10 mM sodium phosphate buffer (pH 7.4). Cells were then treated with the lipophilic fluorescent dye FM^TM^ 4-64 (Thermo Fisher Scientific, USA; final concentration of 13.3 μg/ml) on ice for 1 min to stain the bacterial membrane (*45*). Excess dye was removed by centrifugation, and cells were resuspended with 10 mM sodium phosphate buffer (pH 7.4). Fluorescence microscopic observation was carried out with an inverted fluorescence microscope (Nikon Ti-E, Japan). Cell length was measured with ImageJ 1.53k software.

### Cryo–electron microscope imaging

A drop of a 4-μl sample was applied to a glow-discharged holey carbon grid (Quantifoil Au R2/1 200 mesh). After being blotted for 2 s in 100% humidity at 8°C, the grids were plunged into liquid ethane with Mark IV Vitrobot (Thermo Fisher Scientific, USA). The micrographs were recorded at 73,000× and 45,000× via FEI EPU (Thermo Fisher Scientific, USA) on a 200-kV Glacios microscope (Thermo Fisher Scientific, USA) using a Ceta 16 M camera at a defocus of −3 μm at a total dose of 26 e^−^/Å^2^.

### Phylogenetic analysis

Amino sequences of Lpp were downloaded from the InterPro database (https://ebi.ac.uk/interpro) using the Interpro tag IPR016367 (*46*). A total of 2747 sequences were found in the database. To show the distribution of Lpp in different bacterial clades, one sequence from each bacterial species was kept for phylogenetic analysis. A total of 590 Lpp sequences (data S1) from different bacterial species were used to construct a maximum likelihood phylogenetic tree with MEGA7 (*47*, *48*).

### Statistical analysis

Data are presented as the arithmetic means ± SD. Statistical significance was evaluated using Student’s *t* test. *P* values less than 0.05 were considered statistically significant.

## Notes

View/request a protocol for this paper from *Bio-protocol*.
