# Supplementary material for: In situ visualization of Braun’s lipoprotein on E. coli sacculi
Source: Sci Adv. 2023 Jan 20;9(3):eadd8659. doi: 10.1126/sciadv.add8659 (PMC9858504; doi:10.1126/sciadv.add8659)
Supplement: Supplementary file 1 — Figs. S1 to S10 Tables S1 to S3 [file sciadv.add8659_sm.pdf]

Science Advances
AAAS

Supplementary Materials for

In situ visualization of Braun's lipoprotein on *E.* coli sacculi

Qi Sheng *et al.*

Corresponding author: Simon J. Foster, s.foster@sheffield.ac.uk; Yu-Zhong Zhang, zhangyz@sdu.edu.cn; Hai-Nan Su, suhn@sdu.edu.cn

Sci. Adv. **9**, eadd8659 (2023)
DOI: 10.1126/sciadv.add8659

The PDF file includes:

Figs. S1 to S10
Tables S1 to S3
Legend for data S1

Other Supplementary Material for this manuscript includes the following:

Data S1

Supplementary Figures

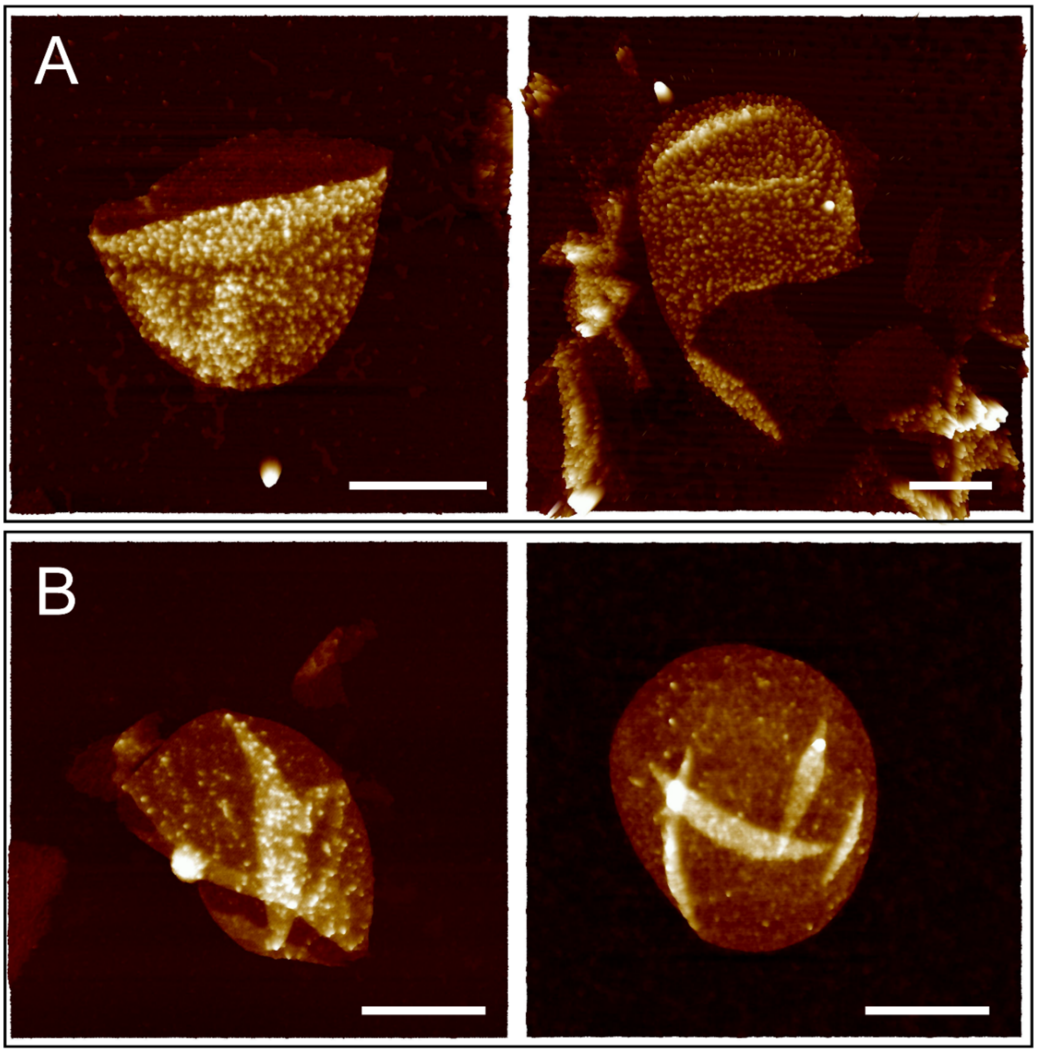

Fig. S1. Particle-like structures on sacculi are sensitive to trypsin treatment. Sacculi from *E. coli* strain MG1655 were isolated following the methods described in the main text. The isolated sacculi were then treated with trypsin (2 mg/mL). After trypsin treatment, the sacculi were washed with MilliQ water and imaged with AFM in ScanAsyst mode in air condition. All images displayed are 3D height images. (**A**) sacculi treated with trypsin for 2 hours. (**B**) sacculi treated with trypsin for 8 hours. The amounts of particle-like structures decrease along with the trypsin treatment times, indicating that these particle-like structures are sensitive to trypsin treatment. Scale bar 500 nm.

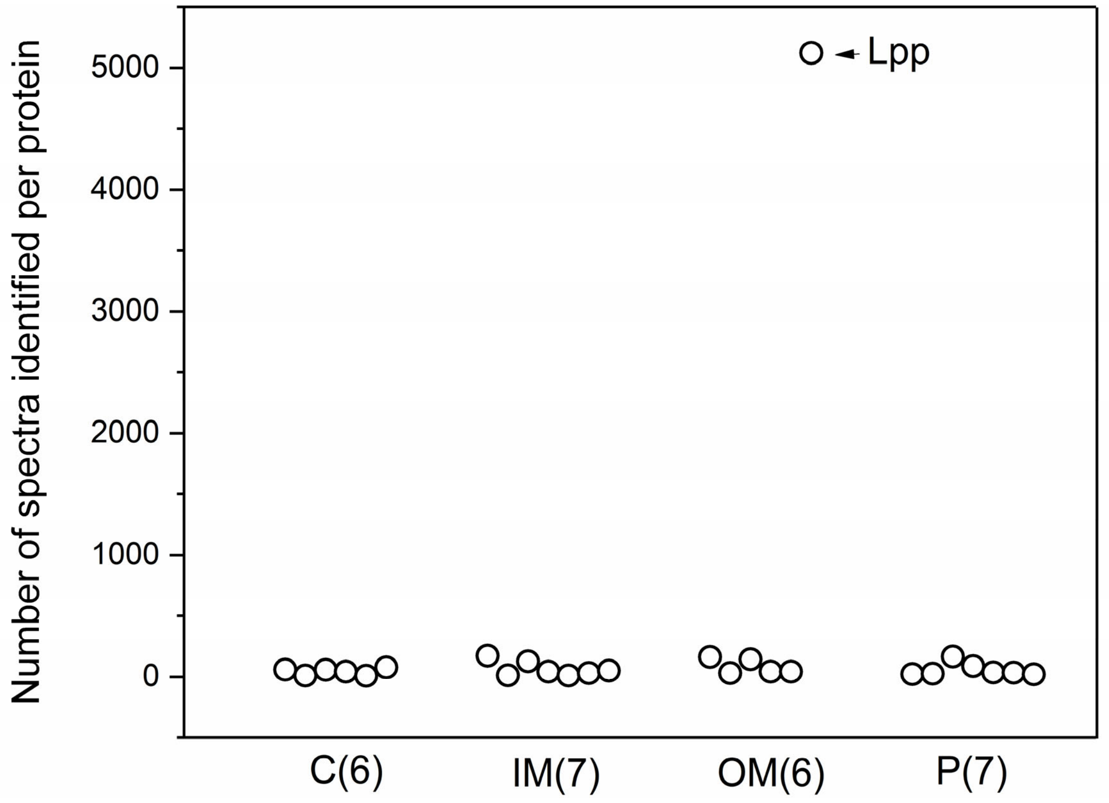

Fig. S2. Lpp is the most predominant protein on isolated sacculi. Sacculi from *E. coli* strain MG1655 were isolated and then treated with trypsin for 12 hours for a full digestion. Peptides were then analyzed by LC-MS/MS. Out of 26 proteins identified, the most predominant protein (in terms of spectra per protein) was predicted to be Lpp. The identified proteins are classified according to their predicted subcellular localization. C, cytoplasmic; IM, inner membrane; OM, outer membrane; P, periplasmic. The numbers in brackets are the number of predicted proteins in each subcellular localization.

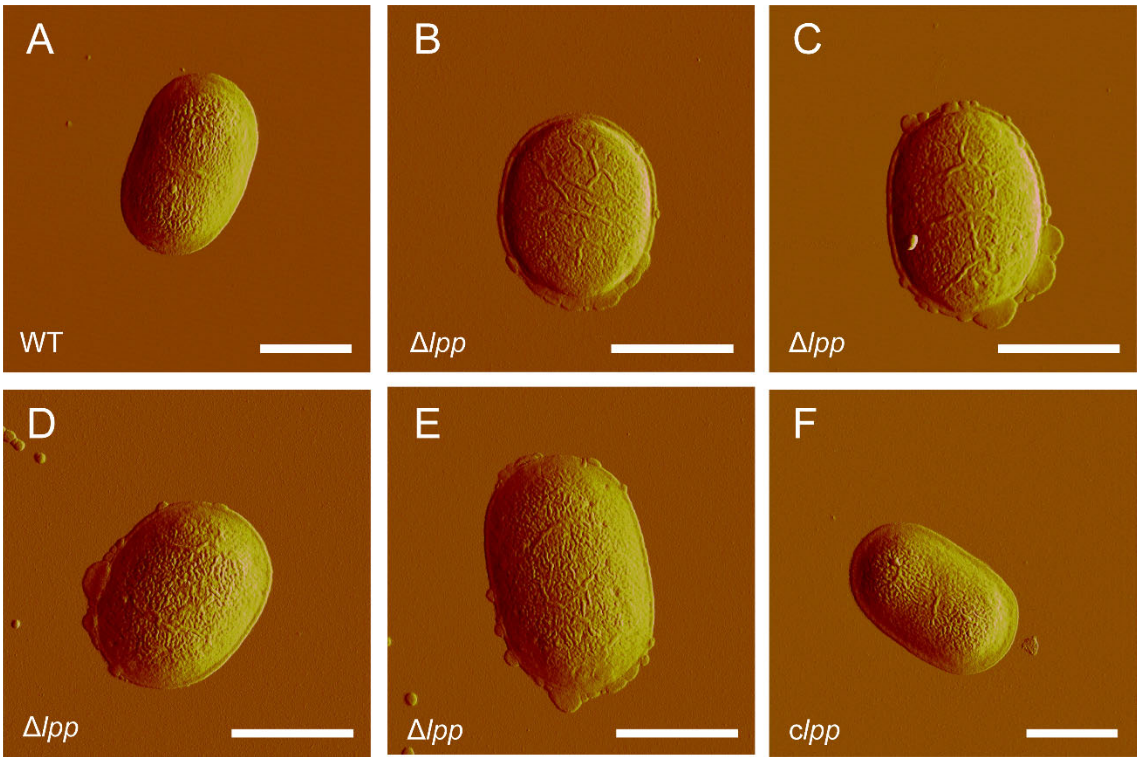

Fig. S3. Deletion of *lpp* lead to formation of outer membrane blebs. The WT (strain MG1655), Δ*lpp* and c*lpp E. coli* strains were cultured using LB medium at 37°C with constant agitation for 12 h. Cells were collected and washed with distilled water, then imaged with AFM in ScanAsyst mode in air. All images displayed are peakforce error images. (**A**) representative image of WT cell. No bleb structure can be noticed on WT cell. (**B-E**) four representative images of Δ*lpp* cells. In the Δ*lpp* strain, outer membrane blebs are formed on the cell surface. (**F**) representative image of c*lpp* cell. When the *lpp* gene was complemented back into Δ*lpp*, the bleb structure is not visualized in c*lpp* cells. Scale bar, 1 µm.

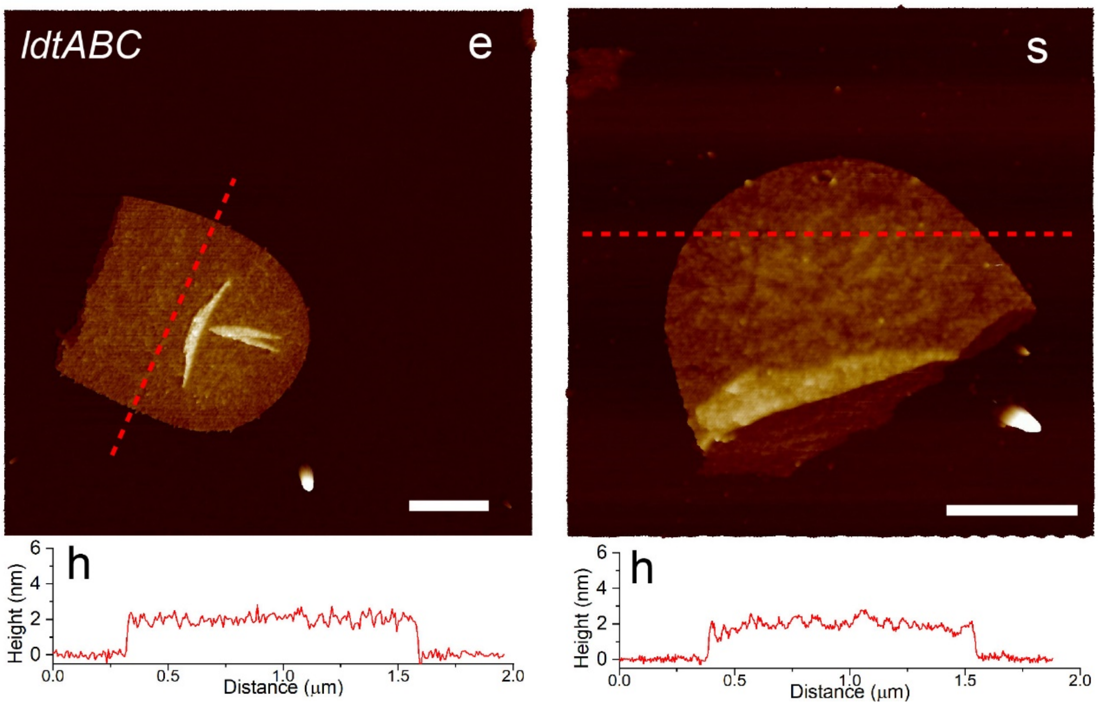

Fig. S4. Role of LdtABC in Lpp sacculus display. AFM was used to observe sacculi from *E. coli* Δ*ldtABC* mutant strain at different growth stages. Sacculi were isolated without the protease treatment step. e, exponential phase. s, stationary phase. h, height profile of sacculi from the corresponding images above. Dashed line indicates position for section analysis. Scale bar 500 nm. After the three L, D-transpeptidase genes *ldtA*, *ldtB*, *ldtC* were deleted, densely distributed Lpp particles on sacculi were not apparent.

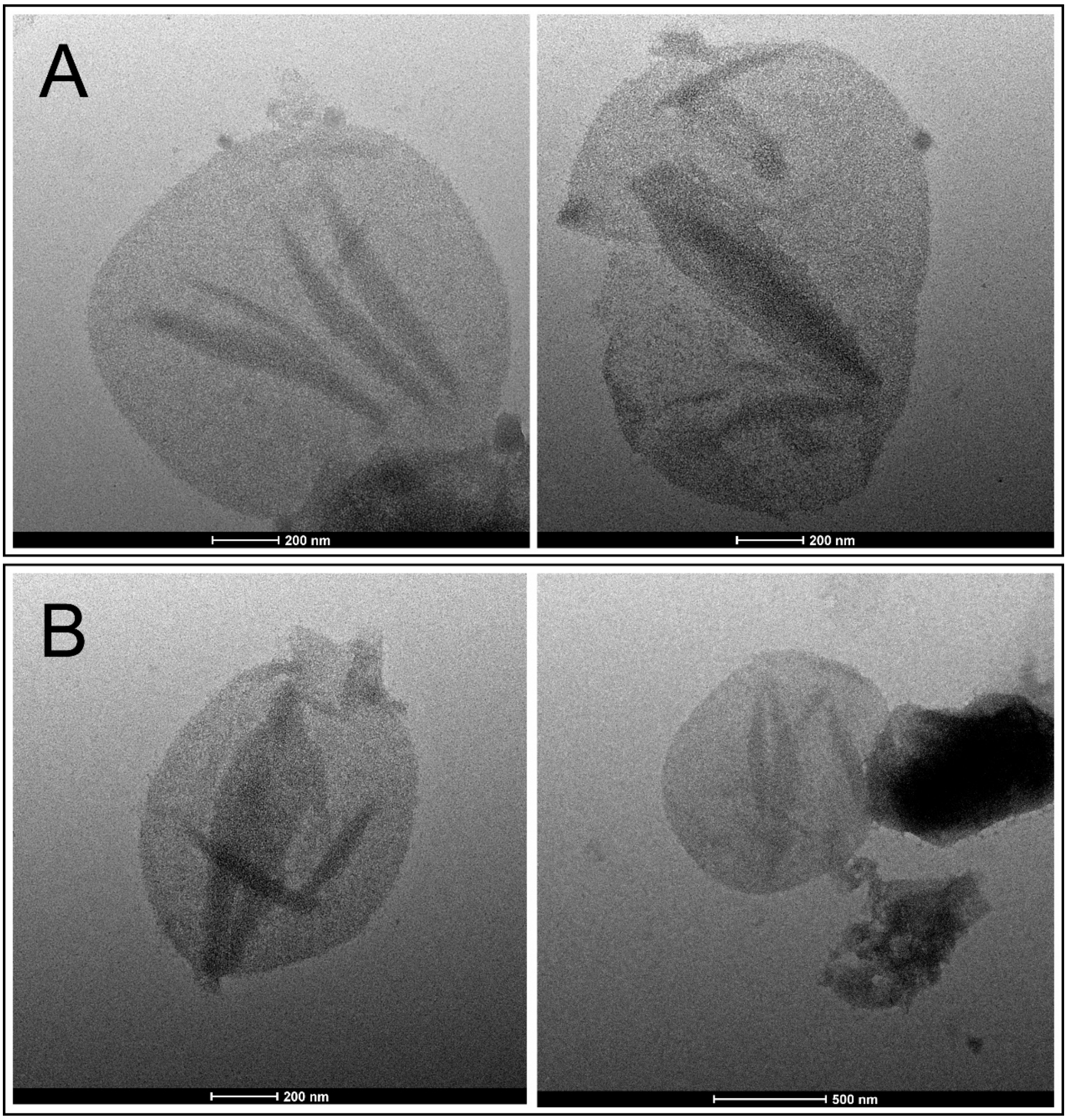

Fig. S5. Lpp on sacculi is not visualized with TEM. Sacculi from *E. coli* strain MG1655 were isolated, without the protease treatment step. The isolated sacculi were then observed with FEI Tecnai G2 F20 TEM. (**A**) sacculi stained with uranyl acetate. (**B**) sacculi stained with both uranyl acetate and lead citrate. Lpp cannot be distinguished from TEM images.

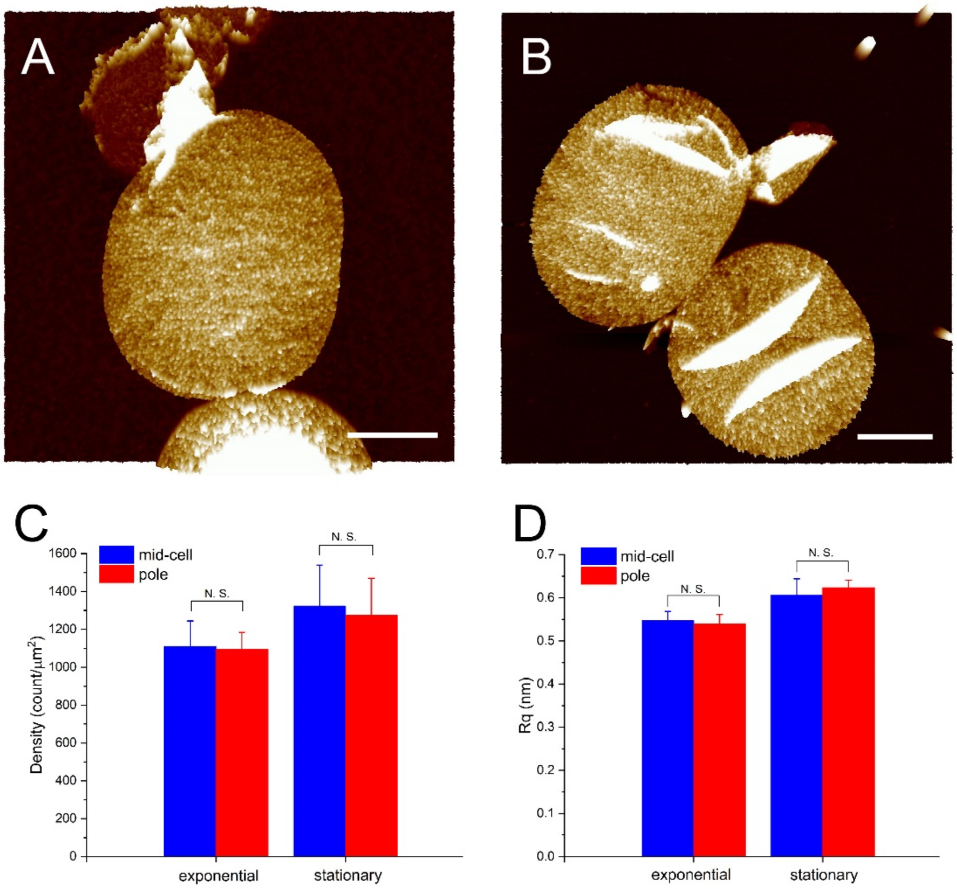

Fig. S6. Subcellular and growth phase analysis of Lpp distribution. Sacculi from *E. coli* strain MG1655 grown in LB at 37 °C to different growth phases were isolated without protease treatment. AFM observations were carried out with ScanAsyst mode in air. (**A**) exponential phase. (**B**) stationary phase. (**C**) density of particle structures on sacculi. (**D**) roughness analysis on sacculi from different growth phases. There is no significant difference in Lpp distribution along the cell axis in non-dividing cells at exponential or stationary growth phase. Scale bar, 500 nm.

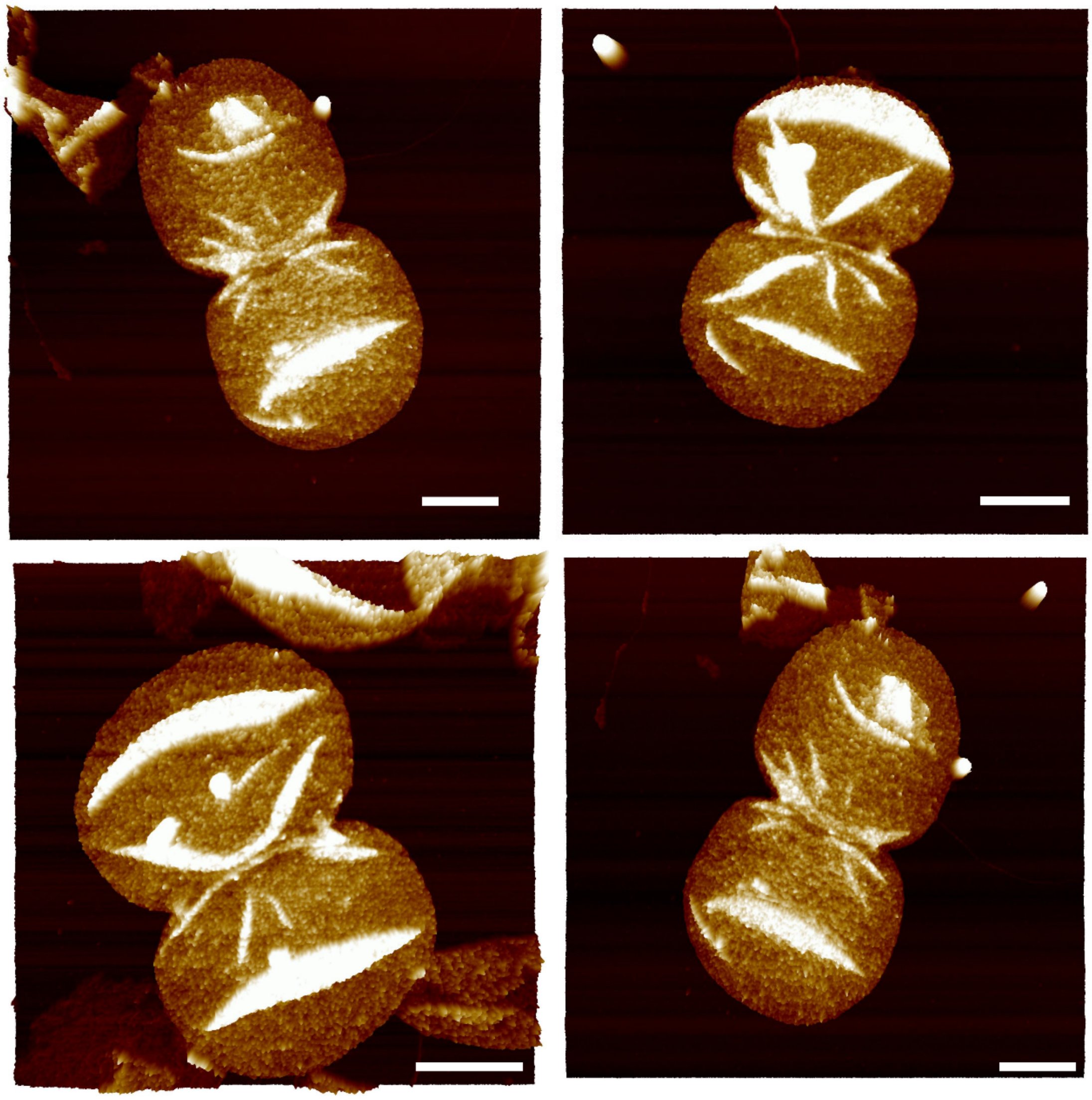

Fig. S7. AFM imaging of *E. coli* sacculi which represent cells at late stages of division. Sacculi from *E. coli* strain MG1655 were isolated without protease treatment. AFM observations were carried out with ScanAsyst mode in air. The sacculi here represent later stages of cell division, compared with Fig. 3A in the main text. However, these sacculi mostly have folds at the constriction sites, maybe due to the geometry of sacculi at this area. These folded sacculi prevented the observation of whether there are groove-like Lpp free regions at the constriction sites. Scale bar, 500 nm.

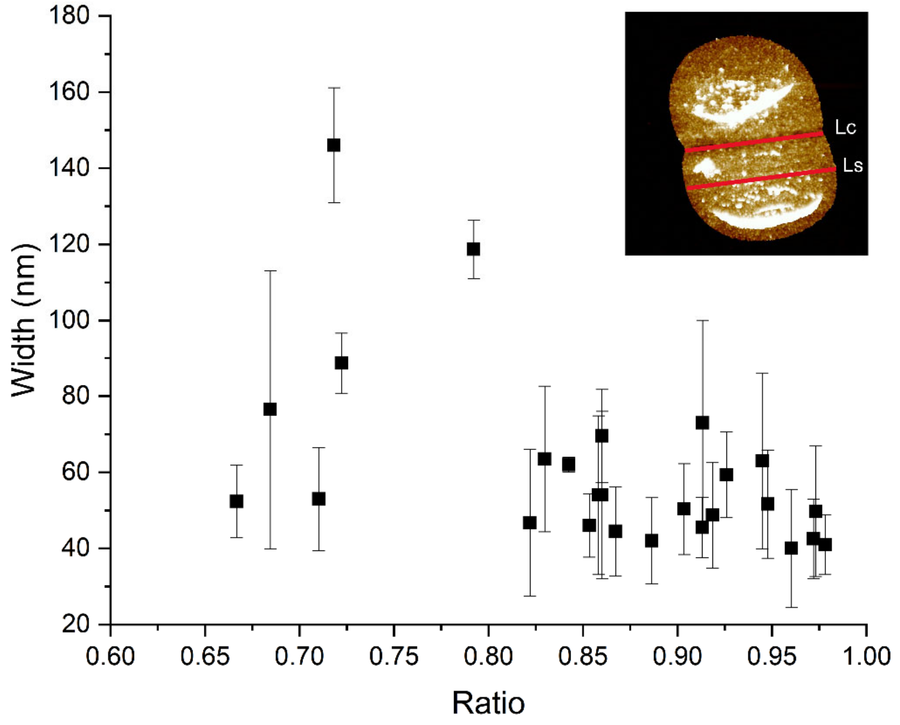

Fig. S8. The width of the Lpp-free groove on sacculi during cell division. Relative division progression was determined by calculating a ratio using the following the equation: Lc/Ls. Ls is the maximum width of sacculi perpendicular to the cell long axis, while Lc is the width of sacculi at the constriction site. The ratio of Lc/Ls is used to roughly estimate the extent of cell division. Smaller values of Lc/Ls represent sacculi at later dividing stages, and *vice versa*. The width of the Lpp-free groove ranges mostly between 40-80 nm at different stages of cell division. Each point in this image is from an individual sacculus (n=26). The groove width data for sacculi with a ratio smaller than 0.65 is not available, because the sacculi that represent later division stages are easily folded at the constriction site (Fig. S7).

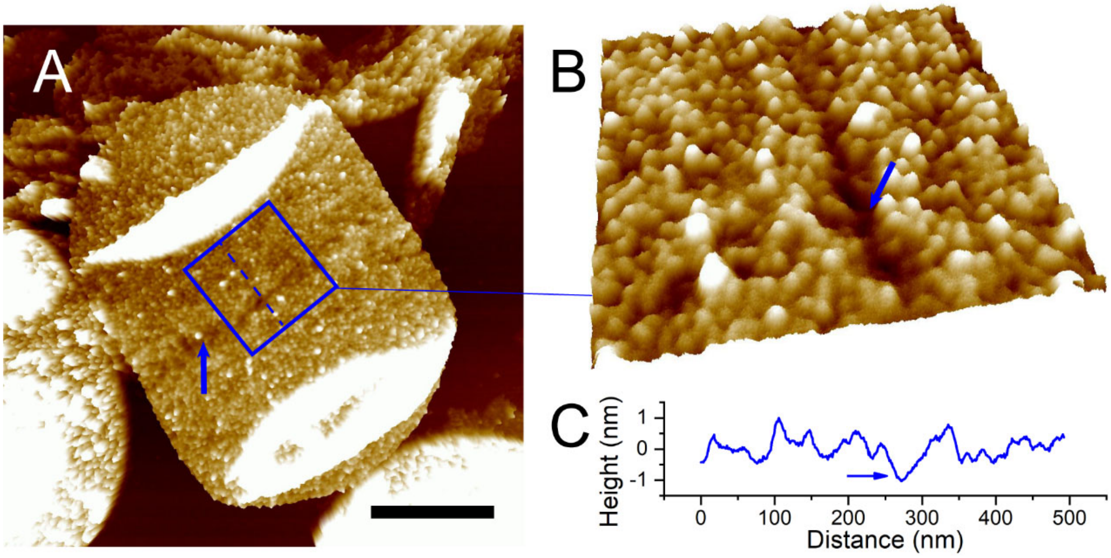

Fig. S9. A narrow Lpp-free groove exists before cell constriction initiates. (A) a groove-like Lpp free region about ~20 nm in width is present on a sacculus. (**B**) higher resolution images of boxed region in A. (**C**) height profiles of sacculi from A. Dashed line in A indicates position for sectional analysis. Arrow indicates the Lpp free region. Scale bar, 500 nm.

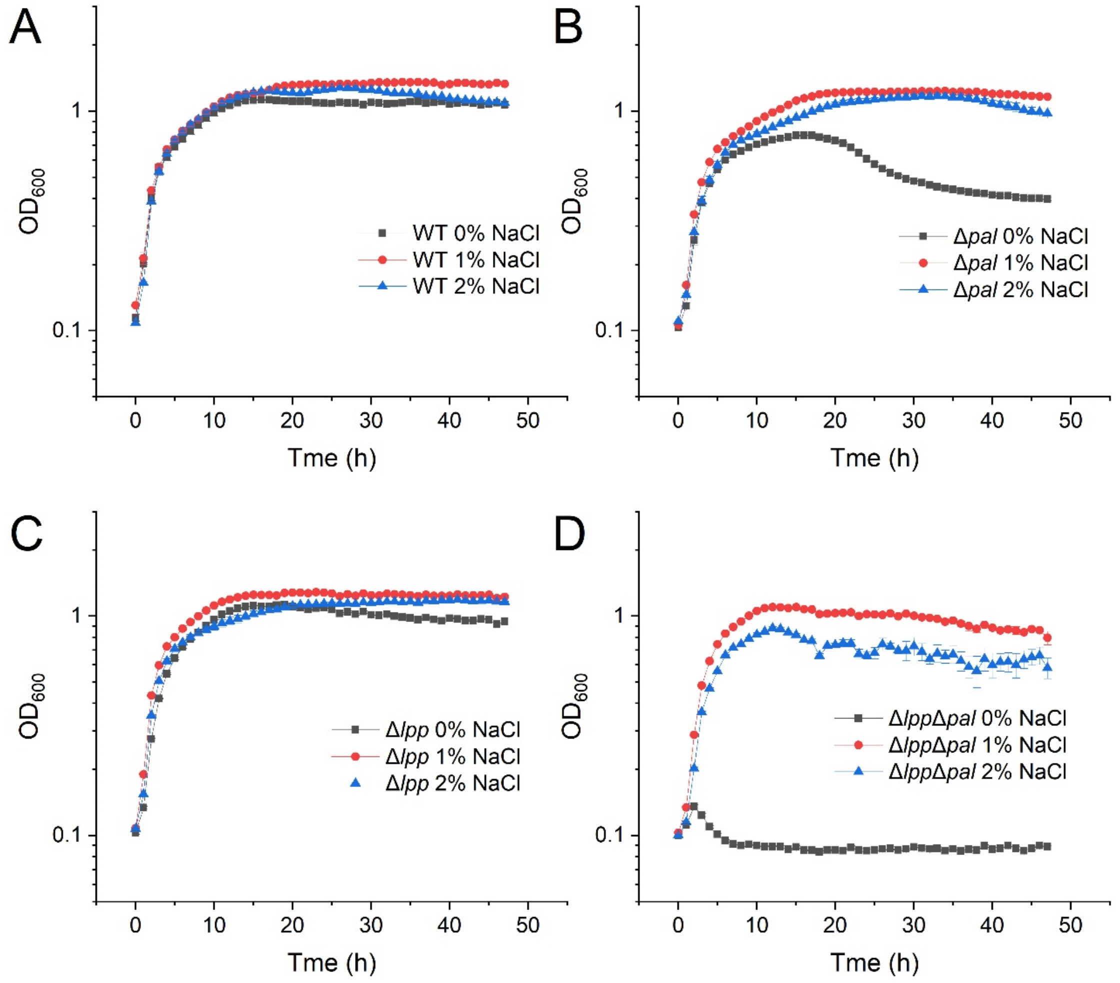

Fig. S10. Growth curves of WT *E. coli* and derived Δ*pal*, Δ*lpp,* and Δ*lpp*Δ*pal* mutants. WT *E. coli* strain MG1655 (**A**) and derived Δ*pal* (**B**), Δ*lpp* (**C**), and Δ*lpp*Δ*pal* (**D**) mutants were incubated at 37°C in LB medium with different NaCl contents (w/v). 0% NaCl, LB medium without sodium chloride; 1% NaCl, standard LB medium; 2% NaCl, LB medium with extra sodium chloride. Growth was monitored with BioScreen C by OD_{600} measurements. Each growth curve plotted represents an average of three readings. WT, Δ*pal*, and Δ*lpp E. coli* grow normally in LB medium with 1% or 2% NaCl, while the Δ*lpp*Δ*pal* strain is viable but grows to a lesser extent. In LB medium without NaCl, growth of Δ*pal* is affected. The double mutant Δ*lpp*Δ*pal* cannot survive in LB medium without NaCl.

Supplementary Tables

Table S1. LC-MS/MS prediction of proteins from sacculi samples of *E. coli* after digestion with trypsin.

Description	Accession	PSMs	Peptides	Predicted subcellular localization
murein lipoprotein	NP_416192.1	5122	13	Outer Membrane
cytochrome bd-I ubiquinol oxidase subunit I	NP_415261.2	172	6	Cytoplasmic Membrane
L,D-transpeptidase LdtE	NP_416193.1	166	5	Periplasmic
outer membrane protein A	NP_415477.1	163	5	Outer Membrane
K(+) binding protein	NP_417151.1	143	5	Outer Membrane
L-glutamate:4-aminobutyrate antiporter	NP_416009.1	126	4	Cytoplasmic Membrane
L,D-transpeptidase LdtA	NP_416494.1	87	6	Periplasmic
DNA protection during starvation protein	NP_415333.1	77	3	Cytoplasmic
glutamate decarboxylase B	NP_416010.1	59	4	Cytoplasmic
translation elongation factor Tu 1	NP_417798.1	57	5	Cytoplasmic
C4-dicarboxylate transporter DcuA	NP_418561.1	51	4	Cytoplasmic Membrane
pyridine nucleotide transhydrogenase subunit beta	NP_416119.1	42	4	Cytoplasmic Membrane
starvation lipoprotein	NP_417963.4	42	3	Outer Membrane
tryptophanase	NP_418164.4	41	3	Cytoplasmic
outer membrane lipoprotein SlyB	YP_025304.1	41	3	Outer Membrane
BOF family protein YgiW	NP_417496.1	35	2	Periplasmic
ribosome- and membrane-associated DUF883 domain-containing protein YqjD	NP_417569.1	34	2	Periplasmic
protease IV, a signal peptide peptidase	NP_416280.1	30	4	Cytoplasmic Membrane
outer membrane porin C	NP_416719.1	30	2	Outer Membrane
L,D-transpeptidase LdtD	NP_415445.1	24	2	Periplasmic
putative lipoprotein YajG	NP_414968.4	21	2	Periplasmic
periplasmic chaperone OsmY	NP_418793.1	19	4	Periplasmic
glucose-specific PTS enzyme IIBC component	NP_415619.1	11	3	Cytoplasmic Membrane

50S ribosomal subunit protein L6	NP_417764.1	10	2	Cytoplasmic
NlpC/P60 family lipoprotein NlpC	NP_416223.1	10	2	Cytoplasmic Membrane
50S ribosomal subunit protein L10	NP_418412.1	7	2	Cytoplasmic

Table S2. A list of bacterial strains used in this study.

Species	Strain	Description	Source
Escherichia coli	MG1655	Wild type strain	a
Escherichia coli	MG1655 Δ*lpp*	*lpp* gene deletion mutant of MG1655	This study
Escherichia coli	MG1655 c*lpp*	Complemented strain of the *lpp* mutant	This study
Escherichia coli	MG1655 Δ*ldtABC*	*ldtA*, *ldtB*, and *ldtC* genes mutant of MG1655	This study
Escherichia coli	MG1655 Δ*pal*	*pal* gene deletion mutant of MG1655	This study
Escherichia coli	MG1655 Δ*pal*Δ*lpp*	*pal* and *lpp* gene deletion mutant of MG1655	This study
Pantoea agglomerans	SDUM028003	Wild type strain	b
Serratia marcescens	SDUM054004	Wild type strain	b
Enterobacter hormaechei	SDUM033005	Wild type strain	b
Hafnia psychrotolerans	18-2	Wild type strain	c

a, kindly provided by Prof. Yongzhen Xia from State Key Laboratory of Microbial Technology, Shandong University.

b, kindly provided by Prof Zongjun Du and Dr Dashuai Mu from Marine College, Shandong University.

c, kept by Hai-Nan Su’s lab.

Table S3. Primers used in this study.

Primer(s)	Sequence(s) (5'-3')
lpp-knock-F	AATACTTGTAACGCTACATGGAGATTAACTCAATCTAGAGGGTATTAATAATGAAAGCAGCATTACACGTCTTGAGCGAT
lpp-knock-R	ACAAAAAAAATGGCGCACAATGTGCGCCATTTTTCACTTCACAGGTACTATTACTTGCGGTATTTAGTCAGGAACACTTAACGGCTGACATG
ldtA-knock-F	ATAAAAGCTATACTTAACGGATAGCTTTCGCGACATAGGAAAGGGACATGATGCGTGCAGCATTACACGTCTTGAGCGAT
ldtA-knock-R	TTCAAAAAAGCCTGCTTTCTAGCAGGCTTTTTGCTTTCTAATTACCAACGCTCTTAAAACATCTGTCTTGACAGGAACACTTAACGGCTGACATG
ldtB-knock-F	CTCTAATATTCTCAACCCAATGGCCTGCCAGGCACAAAATCTCGCTTAACATGAATGCAGCATTACACGTCTTGAGCGAT
ldtB-knock-R	AAATAAAAAAGGCGGATTATGAGTCCGCCTTTTGCTTTAAGTTTTGAAGATTAATTCAGACGAACCGGCAGGAACACTTAACGGCTGACATG
ldtC-knock-F	TCAGGCTTATCTGTTTATTACAATAACCTTATATTTATTATGGATTTTTGGTGATGGCAGCATTACACGTCTTGAGCGAT
ldtC-knock-R	GATTTTTTCCCCGCGACATGCCGTGTCGCGGGGATTTTTTATCCGGGCTTTACAGCGTTTGTGGGCTCAGGAACACTTAACGGCTGACATG
pal-knock-F	GTCGCCGTATCTGTGATAATAATTAATTGAATAGTAAAGGAATCATTGAAATGCAAGCAGCATTACACGTCTTGAGCGAT
pal-knock-R	AACGACAGACTCAATAGTTGATGTCTGAAGTTACTGCTCATGCAATTCTCTTAGTAAACCAGTACCGCCAGGAACACTTAACGGCTGACATG

Other Supplementary Materials for this manuscript

Data S1. (separate file)

Amino sequences of Lpp from different bacterial species downloaded from the InterPro database (https://www.ebi.ac.uk/interpro) used for the phylogenetic analysis.
