# Supplementary material for: In situ visualization of Braun’s lipoprotein on E. coli sacculi
Source: Sci Adv. 2023 Jan 20;9(3):eadd8659. doi: 10.1126/sciadv.add8659 (PMC9858504; doi:10.1126/sciadv.add8659)
Supplement: Supplementary file 2 — Data S1 [file sciadv.add8659_data_s1.zip › sciadv.add8659_data_s1.pdf]

Article Title: ***In situ* visualization of Braun's lipoprotein on *E. coli* sacculi**

Supplementary Materials for this manuscript

Data S1. Amino sequences of Lpp from different bacterial species downloaded from the InterPro database (https://www.ebi.ac.uk/interpro) used for the phylogenetic analysis:

>A0A6H3ED51| Acinetobacter baumannii
MNRTKLVLGAVILGSTLLAGCSSNAKIDQLSSDVQTLNAKVDQLSNDVNAMR
SDVQAAKDDAARANQRLDNQAHSYRK
>A0A0T6UY96| Aeromonas allosaccharophila
MKKLLLVGVVGLSALLGGCANTSELETSVQNLSNKVDQLAQDVSAVRADQS
KIAADVAEAKQEAMRANQRIDNMATSYKK
>A0A2H9U2J6| Aeromonas cavernicola
MKKMLLVGVVGLSALLGGCANTSELETSVQNLSNKVDQLAQDVSAVRADQS
KIAADVAEAKQEAMRANQRIDNMATSYKK
>A0A081LHZ1| Aeromonas caviae
MKKMLLVGVVGLSALLGGCANTSELETSVQNLSNKVDQLAQDVSAVRADQS
KIAADVAEAKQEAMRANQRIDNMATSYKK
>A0A433G800|Aeromonas dhakensis
MKKMLLVGVVGLSALLGGCANTSELETSVQNLSNKVDQLAQDVSAVRADQS
KIAADVAEAKQEAMRANQRIDNMATSYKK
>N9TW73|Aeromonas diversa
MKKMLLVGVVGLSALLGGCANTELETSVQNLSNKVDQLAQDVSAVRADQS
KIAADVADAKAEAARANQRLDNMATSYKK
>A0A175VJE8|Aeromonas enteropelogenes
MKKLLLVGVVGLSALLGGCANTSELETSVQNLSNKVDQLAQDVSAVRADQS
KIAADVAEAKQEAMRANQRIDNMATSYKK
>A0A7W7ULK3|Aeromonas fluvialis
MKKLLLVGVVGLSALLGGCANTSELETSVQNLSNKVDQLAQDVSAVRADQS
KIAADVAEAKQEAMRANQRIDNMATSYKK
>A0A081UTT1|Aeromonas hydrophila
MKKMLLVGVVGLSALLGGCANTSELETSVQNLSNKVDQLAQDVSAVRADQS
KIAADVAEAKQEAMRANQRIDNMATSYKK
>A0A2S5FF78|Aeromonas jandaei
MKKLLLVGVVGLSALLGGCANTSELETSVQNLSNKVDQLAQDVSAVRADQS
KIAADVAEAKQEAMRANQRIDNMATKYKK
>A0A2M8H8F0|Aeromonas lusitana
MKKMLLVGVVGLSALLGGCANTSELETSVQNLSNKVDQLAQDVSAVRADQS
KIAADVATANQEAMRANQRIDNMATSYKK
>A0A6M4YC58|Aeromonas media

MKKMLLVGVVGLSALLGGCANTSELETSVQNLSNKVDQLAQDVSAVRADQS
KMAADIATANQEAARANQRIDNMATSYKK
>R1HBK5|Aeromonas molluscorum
MKKLLLVGVVGLSALLGGCANTSELETSVQNLSNKVDQLAQDVSAVRADQS
KMAADVAQANQEAMRANQRLDNMATSYKK
>A0A091AE99|Aeromonas salmonicida
MKKLLLVGVVGLSALLGGCANTSELETSVQNLSNKVDQLAQDVSAVRADQS
KIAADVAEAKQEAMRANQRIDNMATSYKK
>A0A0S2SE52|Aeromonas schubertii
MKKMLLVGVVGLSALLGGCANTELETSVQNLSNKVDQLAQDVSAVRADQS
KIAADVADAKAEAARANRRLDNMATSYKK
>A0A5J6WV94|Aeromonas simiae
MKKMLLVGVVGLSALLGGCANTELETSVQNLSNKVDQLAQDVSAVRADQS
KIAADVADAKAEAARANQRLDNMATSYKK
>A0A1S2CNY0|Aeromonas sobria
MKKLLLVGVVGLSALLGGCANTSELETSVQNLSNKVDQLAQDVSAVRADQS
KIAADVSEAKQEAMRANQRIDNMATSYKK
>A0A5F0K7G7|Aeromonas taiwanensis
MKKMLLVGVVGLSALLGGCANTSELETSVQNLSNKVDQLAQDVSAVRADQS
KIAADVAEAKQEAMRANQRIDNMATSYKK
>A0A6S4V2T5|Aeromonas veronii
MKKLLLVGVVGLSALLGGCANTSELETSVQNLSNKVDQLAQDVSAVRADQS
KIAADVAEAKQEAMRANQRIDNMATKYKK
>R9PNI8|Agarivorans albus
MNKFLVAGAIASTILLAGCSSNTTALENNVQSLSDKVDALSNEVAALRAEQSS
LSGDIKSAQMSADQAKAEAMRANERIDNIASSYTK
>A0A420ED24|Alginatibacterium sediminis
MKKLLIAGAIVSTFALAGCSNQTTTEQESFTLNDQVEALAAEVAALRAEQDA
ANAAQMTADQAKADAMRANERLDNMATSYTK
>A0A2X0WL41| Anaerobiospirillum thomasii
MTKYSALLGALALGATALVGCTSPALDQLNAKVDAIAADVDALKTQQSKLA
NDVAFVKSDAARANERLDNLARRYKK
>D2TW17| Arsenophonus nasoniae
MNRTKVVLGSIVLASALLAGCSNSSQINKLSSDVQTLNGKVDQLSNDVQSLR
SDVQTAKDEAARANQRLDNQVRRYKK
>A0A447LNC1| Atlantibacter hermannii
MNRTKLVLGAVILGSTLLAGCSSNAKIDQLSSDVQTLNAKVDQLSNDVNAMR
SDVQAAKDDAARANQRLDNQATKYRK
>A0A427V212| Atlantibacter subterranea
MNRTKLVLGAVILGSTLLAGCSSNAKIDQLSSDVQTLNAKVDQLSNDVNAMR
SDVQAAKDDAARANQRLDNQATKYRK
>Q1LTZ8| Baumannia cicadellinicola subsp. Homalodisca coagulata

MTLNKLVLGAVMLSSTLMAGCSCSEQIDKLNSSIKSNIKTLDTKFEQKFNQIN
RDVHTIQTDIKSAKDEVATMQTDMKSAKDEVATMQTDIKLAKSEAARAHIRL
NNQSSAYHK
>A0A4R3Z3L9| Biostraticola tofi
MNRTKLVLGAVILGSTLLAGCSSNAKIDQLSSDVQTLNAKVDQLSNDVTAIRS
DVQAAKDDAARANQRLDNQATSYRK
>Q7VR58| Blochmannia floridanus
MNFRKLICFTLTISSLILIGCASTSQITELSSEIQKLNRKIDQVNENINVLNPAIEQI
KNEANRANKRLDNQISIYKK
>Q494E3| Blochmannia pennsylvanicus
MNRNRLICSAIILSSFVLANCASTNKIKELSSEVHDLNERIDQISSDIDVLRPEIE
KTKNEAARANQRIDNQITSYHK
>E8Q667| Blochmannia vafer
MNNKILMYFVTVFSTLILVSCASTTKITELSAEVQELSTRLDQLNSNVDILKPEI
EQAKNEAHRANKRLDNQISLYRK
>A0A421DRY9|Brenneria alni
MNRTKLVLGAVILGSTLLAGCSSNAKIDQLSSDVQTLNAKVDQLSNDVNAIRS
DVQAAKDDAARANQRLDNQVRTYKK
>A0A0G4JZC5|Brenneria goodwinii
MNRTKLVLGAVILGSTLLAGCSSNAKIDQLSSDVQTLNAKVDQLSNDVNAIRS
DVQAAKDDAARANQRLDNQVRTYKK
>A0A2U1USB4|Brenneria nigrifluens
MNRTKLVLGAVILGSTLLAGCSSNAKIDQLSSDVQTLNAKVDQLSNDVNAIRS
DVQAAKDDAARANQRLDNQVRTYKK
>A0A2U1TXJ6|Brenneria roseae
MNRTKLVLGAVILGSTLLAGCSSNAKIDQLSSDVQTLNAKVDQLSNDVNAIRS
DVQAAKDDAARANQRLDNQVRTYKK
>A0A4P8QN49|Brenneria rubrifaciens
MNRTKLVLGAVILGSTLLAGCSSNAKIDQLSSDVQTLNAKVDQLSNDVNAIRS
DVQAAKDDAARANQRLDNQVRTYKK
>A0A366I6K2|Brenneria salicis
MNRTKLVLGAVILGSTLLAGCSSNAKIDQLSSDVQTLNAKVDQLSNDVNAIRS
DVQAAKDDAARANQRLDNQVRTYKK
>A0A4U2UHX9| Bruguierivorax albus
MNRTKLVLGAVILGSTLLAGCSSNAKIDQLSSDVQTLNTKVDQLSNDVNAIRS
DVQAAKDDAARANQRLDNQAHSYRK
>A0A2C6DPH4| Budvicia aquatica
MNRTKLVLGAVILGATLLAGCAKTDTTTNSKLTQLISDVAALRSDVQVAKDEA
ARANQRLDNMTRSYKK
>A0A381C8P8|Buttiauxella agrestis
MNRTKLVLGAVILASTMLAGCSSNAKIDQLSSDVQTLNAKVDQLSNDVNAM
RSDVQAAKDDAARANQRLDNQAHSYRK

>A0A1B7ISF9|Buttiauxella brennerae
MNRTKLVLGAVILASTMLAGCSSNAKIDQLSSDVQTLNAKVDQLSNDVNAM
RSDVQAAKDDAARANQRLDNQAHSYRK
>A0A1B7HQ03|Buttiauxella gaviniae
MNRTKLVLGAVILASTMLAGCSSNAKIDQLSSDVQTLNAKVDQLSNDVNAM
RSDVQAAKDDAARANQRLDNQAHSYRK
>A0A3A5JVS8|Buttiauxella izardii
MNRTKLVLGAVILASTMLAGCSSNAKIDQLSSDVQTLNAKVDQLSNDVNAM
RSDVQAAKDDAARANQRLDNQAHSYRK
>A0A1B7HX51|Buttiauxella noackiae
MNRTKLVLGAVILASTMLAGCSSNAKIDQLSSDVQTLNAKVDQLSNDVNAM
RSDVQAAKDDAARANQRLDNQAHSYRK
>A0A3N5DG22|Buttiauxella warmboldiae
MNRTKLVLGAVILASTMLAGCSSNAKIDQLSSDVQTLNAKVDQLSNDVNAM
RSDVQAAKDDAARANQRLDNQAHSYRK
>S3IZ65|Cedecea davisae
MNRTKLVLGAVILASTMLAGCSSNAKIDQLSSDVQTLNAKVDQLSNDVNAVR
SDVQAAKDDAARANQRLDNQAHSYRK
>A0A2N0CWH1|Cedecea lapagei
MNRTKLVLGAVILASTMLAGCSSNAKIDQLSSDVQTLNAKVDQLSNDVNAIR
SDVQAAKDDAARANQRLDNQATKYRK
>A0A089UXR1|Cedecea neteri
MNRTKLVLGAVILASTMLAGCSSNAKIDQLSSDVQTLNAKVDQLSNDVNAVR
SDVQAAKDDAARANQRLDNQAHSYRK
>A0A4R1K415| Celerinatantimonas diazotrophica
MKKTLLIAGALAAPLLLAGCSNTSNKTLSNKLDSLSDQVSALQAEQSTLSSDV
NSAKMAAQEAKSEAARANQRIDHIAKSYTK
>W0LEU4| Chania multitudinisentens
MNRTKLVLGAVILASTMLAGCSSNAKIDQLSSDVQTLNAKVDQLSNDVNAIR
SDVQAAKDDAARANQRLDNQAHAYKK
>A0A2N5ESX3|Chimaeribacter arupi
MNRTKLVLGAVILGSTLLAGCSSNAKIDQLSSDVQTLNAKVDQLSNDVNAIRS
DVQAAKDDAARANQRLDNQAHAYKK
>A0A2N5EGB1|Chimaeribacter californicus
MNRTKLVLGAVILGSTLLAGCSSNAKIDQLSSDVQTLNAKVDQLSNDVNAIRS
DVQAAKDDAARANQRLDNQAHAYKK
>A0A2N5EDG5|Chimaeribacter coloradensis
MNRTKLVLGAVILGSTLLAGCSSNAKIDQLSSDVQTLNAKVDQLSNDVNAIRS
DVQAAKDDAARANQRLDNQAHAYKK
>A0A2S4RZ28|Citrobacter amalonaticus
MNRTKLVLGAVILGSTLLAGCSSNAKIDQLSSDVQTLNAKVDQLSNDVNAMR
SDVQAAKDDAARANQRLDNQATKYRK

>A0A1R0G303|Citrobacter braakii
MNRTKLVLGAVILGSTLLAGCSSNAKIDQLSSDVQTLNAKVDQLSNDVNAMR
SDVQAAKDDAARANQRLDNAATKYRK
>A0A7X1EGD8|Citrobacter cronae
MNRTKLVLGAVILGSTLLAGCSSNAKIDQLSSDVQTLNAKVDQLSNDVNAMR
SDVQAAKDDAARANQRLDNAATKYRK
>A0A223JRB5|Citrobacter farmeri
MNRTKLVLGAVILGSTLLAGCSSNAKIDQLSSDVQTLNAKVDQLSNDVNAMR
SDVQAAKDDAARANQRLDNQATKYRK
>A0A0A5P5A5|Citrobacter freundii
MNRTKLVLGAVILGSTLLAGCSSNAKIDQLSSDVQTLNAKVDQLSNDVNAMR
SDVQAAKDDAARANQRLDNAATKYRK
>A0A3E2KR45|Citrobacter gillenii
MNRTKLVLGAVILGSTLLAGCSSNAKIDQLSSDVQTLNAKVDQLSNDVNAMR
SDVQAAKDDAARANQRLDNAATKYRK
>A0A078L9Y2|Citrobacter koseri
MNRTKLVLGAVILGSTLLAGCSSNAKIDQLSSDVQTLNAKVDQLSNDVNAMR
SDVQAAKDDAARANQRLDNQATKYRK
>A0A6N6K3E9|Citrobacter pasteurii
MNRTKLVLGAVILGSTLLAGCSSNAKIDQLSSDVQTLNAKVDQLSNDVNAMR
SDVQAAKDDAARANQRLDNAATKYRK
>A0A5B0TAJ5|Citrobacter portucalensis
MNRTKLVLGAVILGSTLLAGCSSNAKIDQLSSDVQTLNAKVDQLSNDVNAMR
SDVQAAKDDAARANQRLDNAATKYRK
>A0A482PJB8|Citrobacter rodentium
MNRTKLVLGAVILGSTLLAGCSSNAKIDQLSSDVQTLNAKVDQLSNDVNAMR
SDVQAAKDDAARANQRLDNQATKYRK
>A0A6L5EJ39|Citrobacter telavivum
MNRTKLVLGAVILGSTLLAGCSSNAKIDQLSSDVQTLNAKVDQLSNDVNAMR
SDVQAAKDDAARANQRLDNQATKYRK
>A0A4P7J362|Citrobacter tructae
MNRTKLVLGAVILGSTLLAGCSSNAKIDQLSSDVQTLNAKVDQLSNDVNAMR
SDVQAAKDDAARANQRLDNAATKYRK
>A0A5P2MH95|Citrobacter werkmanii
MNRTKLVLGAVILGSTLLAGCSSNAKIDQLSSDVQTLNAKVDQLSNDVNAMR
SDVQAAKDDAARANQRLDNAATKYRK
>A0A549V7D8|Citrobacter youngae
MNRTKLVLGAVILGSTLLAGCSSNAKIDQLSSDVQTLNAKVDQLSNDVNAMR
SDVQAAKDDAARANQRLDNAATKYRK
>A0A222G9E9|Colwellia beringensis
MLLKKLTLVALVAIISGCANNSALEANISQLNQKVDNLTEKVNSLSSQTKAVSA
EVNELGMAQEQTNQAVKDTNERIDNVVASYKK

>A0A1H7J5Z4|Colwellia chukchiensis
MLLKKLTIVTFVAVLSGCANNSALEENISRLNQKVDSLSEQVNSLNSKTQKLS
GKVNDLSAAQKQTSKAVNATQMAVDKTNERIDNMVASYKK
>A0A5C6QII1|Colwellia demingiae
MLKKITAVSLALALSACANTDALDANITSLTSKVDALSSQVSDLEAQQQSVSA
DVQAAKAAAEQAATDAKAANERIDNVVASYKK
>A0A5C6QAI2|Colwellia hornerae
MLIKTIALAALIMTATGCANTDALEASVSSLNQKVDTLTNKVNALTDEVADVK
SQQTMNTETIEGVKSSVANANERMDNIASSYKK
>A0A1Z5I520|Colwellia marinimaniae
MLKKITAISLALTLTACANTEALDANISSLTSKVAALSAQVADLEVQQQSMASD
AKAAKSAAEQAASDAKAANERIDNVVASYKK
>A0A5S3P3F5|Colwellia ponticola
MIKTITAATLALALTACSNTDALDANISSLTNKVDTLSSQVANLESQQQTIAND
AAAAKKAAQRAATDAKEANQRIDNVVASYKK
>A0A099KF85|Colwellia psychrerythraea
MLKKITAISLALTLTACANTDALDANITSLTNKVDALSAQVADLEAQQQASAA
DVQAAKSAAEQAASDAKAANERIDNVVASYKK
>K8A2K3|Cronobacter condimenti
MNRTKLVLGAVILGSTLLAGCSSNAKIDQLSSDVQTLNAKVDQLSNDVNAMR
SDVQAAKDDAARANQRLDNMATKYRK
>K8AP89|Cronobacter dublinensis
MNRTKLVLGAVILGSTLLAGCSSNAKIDQLSSDVQTLNAKVDQLSNDVNAMR
SDVQAAKDDAARANQRLDNMATKYRK
>V5TZI5|Cronobacter malonaticus
MNRTKLVLGAVILGSTLLAGCSSNAKIDQLSSDVQTLNAKVDQLSNDVNAMR
SDVQAAKDDAARANQRLDNMATKYRK
>A0A2T7AS91|Cronobacter muytjensii
MNRTKLVLGAVILGSTLLAGCSSNAKIDQLSSDVQTLNAKVDQLSNDVNAMR
SDVQAAKDDAARANQRLDNMATKYRK
>A0A2S9U986|Cronobacter sakazakii
MNRTKLVLGAVILGSTLLAGCSSNAKIDQLSSDVQTLNAKVDQLSNDVNAMR
SDVQAAKDDAARANQRLDNMATKYRK
>A0A2T7B7X9|Cronobacter turicensis
MNRTKLVLGAVILGSTLLAGCSSNAKIDQLSSDVQTLNAKVDQLSNDVNAMR
SDVQAAKDDAARANQRLDNMATKYRK
>A0A376ECN4|Cronobacter universalis
MNRTKLVLGAVILGSTLLAGCSSNAKIDQLSSDVQTLNAKVDQLSNDVNAMR
SDVQAAKDDAARANQRLDNMATKYRK
>A0A1G2W8J8| Desulfuromonadaceae bacterium GWB2_53_15
MKIRTMVIAMAAVLAMTLTGCATSRQMEKVEADQKLLDAKVEQALQNAQV
AKTAADEAKQKAADATSRAEQAEKAAQEREQLANEKMQRADAAFQKSMRK

>A0A375ABX1|Dickeya aquatica
MNRTKLVLGAVILGSTLLAGCSSNAKLDQLSSDVSSLNEKVSALTSKVDALAT
DVQAAKDDAARANQRLDNQVRTYKK
>C6CFM8|Dickeya chrysanthemi
MNRTKLVLGAVILGSTLLAGCSSNAKLDQLSSDVSSLNEKVSALTSKVDALAT
DVQAAKDDAARANQRLDNQVRTYKK
>E0SGT5|Dickeya dadantii
MNRTKLVLGAVILGSTLLAGCSSNAKLDQLSSDVSSLNEKVSALTSKVDALAT
DVQAAKDDAARANQRLDNQVRTYKK
>A0A3A4CUU0|Dickeya dianthicola
MNRTKLVLGAVILGSTLLAGCSSNAKLDQLSSDVSSLNEKVSALTSKVDALAT
DVQAAKDDAARANQRLDNQVRTYKK
>A0A2K8QPD6|Dickeya fangzhongdai
MNRTKLVLGAVILGSTLLAGCSSNAKLDQLSSDVSSLNEKVSALTSKVDALAT
DVQAAKDDAARANQRLDNQVRTYKK
>C6C486|Dickeya paradisiaca
MNRTKLVLGAVILGSTLLAGCSSNAKLDQLSSDVSSLNDKVSALTSKVDALAT
DVQAAKDDAARANQRLDNQVRTYKK
>A0A5B8IA70|Dickeya poaceiphila
MNRTKLVLGAVILGSTLLAGCSSNAKLDQLSSDVSSLNEKVSALTSKVDALAT
DVQAAKDDAARANQRLDNQVRTYKK
>A0A2K8VV11|Dickeya solani
MNRTKLVLGAVILGSTLLAGCSSNAKLDQLSSDVSSLNEKVSALTSKVDALAT
DVQAAKDDAARANQRLDNQVRTYKK
>A0A3N0G4W6|Dickeya undicola
MNRTKLVLGAVILGSTLLAGCSSNAKLDQLSSDVSSLNEKVSALTSKVDALAT
DVQAAKDDAARANQRLDNQVRTYKK
>A0A2K9QG76|Dickeya zeae
MNRTKLVLGAVILGSTLLAGCSSNAKLDQLSSDVSSLNEKVSALTSKVDALAT
DVQAAKDDAARANQRLDNQVRTYKK
>A0A076LY14|Edwardsiella anguillarum
MNRTKLVLGAVILGSTLLAGCSSNAKIDQLSSDVQTLNTKVDQLSSDVNAMR
ADVQAAKDDAARANQRLDNMAHAYKK
>A0A376DGD9|Edwardsiella hoshinae
MNRTKLVLGAVILGSTLLAGCSSNAKIDQLSSDVQTLNTKVDQLSSDVNAMR
ADVQAAKDDAARANQRLDNMAHAYKK
>C5BGU3| Edwardsiella ictaluri
MNRTKLVLGAVILGSTLLAGCSSNAKINQLSSDVQTLNTKVDQLSSDVNAMR
ADVQAAKDDAARANQRLDNMAHAYKK
>A0A034STA9| Edwardsiella piscicida
MNRTKLVLGAVILGSTLLAGCSSNAKIDQLSSDVQTLNTKVDQLSSDVNAMR
ADVQAAKDDAARANQRLDNMAHAYKK

>A0A2A7U3E0| Edwardsiella tarda
MNRTKLVLGAVILGSTLLAGCSSNAKIDQLSSDVQTLNTKVDQLSSDVNAMR
ADVQAAKDDAARANQRLDNMAHAYKK
>A0A370R3G9| Enterobacillus tribolii
MNRTKLVLGAVILGSTLLAGCSSNAKIDQLSSDVQTLNAKVDQLSNDVNAMR
SDVQAAKDDAARANQRLDNQTRSYKK
>A0A1T4KN39|Enterobacter agglomerans
MNRTKLVLGAVVLASTMLAGCSSNAKIDQLSSDVQTLNAKVDQLSNDVNAV
RSDVQAAKDDAARANQRLDNQAHSYRK
>A0A0F0RLB2|Enterobacter asburiae
MNRTKLVLGAVILGSTLLAGCSSNAKIDQLSSDVQTLNAKVDQLSNDVNAIRS
DVQAAKDDAARANQRLDNQATKYRK
>A0A2J7SZI3|Enterobacter bugandensis
MNRTKLVLGAVILGSTLLAGCSSNAKIDQLSSDVQTLNAKVDQLSNDVNAMR
SDVQAAKDDAARANQRLDNQATKYRK
>A0A0A3Z2M5|Enterobacter cancerogenus
MNRTKLVLGAVILGSTLLAGCSSNAKIDQLSSDVQTLNAKVDQLSNDVNAIRS
DVQAAKDDAARANQRLDNQAHSYRK
>A0A5C1BZ52|Enterobacter chengduensis
MNRTKLVLGAVILGSTLLAGCSSNAKIDQLSSDVQTLNAKVDQLSNDVNAMR
SDVQAAKDDAARANQRLDNQATKYRK
>A0A3A3ZUS0|Enterobacter chuandaensis
MNRTKLVLGAVILGSTLLAGCSSNAKIDQLSSDVQTLNAKVDQLSNDVNAMR
SDVQAAKDDAARANQRLDNQATKYRK
>A0A094ZLD6|Enterobacter cloacae
MNRTKLVLGAVILGSTLLAGCSSNAKIDQLSSDVQTLNAKVDQLSNDVNAMR
SDVQAAKDDAARANQRLDNQATKYRK
>A0A5A9BJM0|Enterobacter dykesii
MNRTKLVLGAVILGSTLLAGCSSNAKIDQLSSDVQTLNAKVDQLSNDVNAMR
SDVQAAKDDAARANQRLDNQATKYRK
>A0A330D9P0|Enterobacter hormaechei
MNRTKLVLGAVILGSTLLAGCSSNAKIDQLSSDVQTLNAKVDQLSNDVNAMR
SDVQAAKDDAARANQRLDNQATKYRK
>A0A3R9PX53|Enterobacter huaxiensis
MNRTKLVLGAVILGSTLLAGCSSNAKIDQLSSDVQTLNAKVDQLSNDVNAIRS
DVQAAKDDAARANQRLDNQATKYRK
>A0A0F0XSW1|Enterobacter kobei
MNRTKLVLGAVILGSTLLAGCSSNAKIDQLSSDVQTLNAKVDQLSNDVNAMR
SDVQAAKDDAARANQRLDNQATKYRK
>E3G5Z9|Enterobacter lignolyticus
MNRTKLVLGAVILGSTLLAGCSSNAKIDQLSSDVQTLNAKVDQLSNDVNAMR
SDVQAAKDDAARANQRLDNQAHSYRK

>G8LLF0|Enterobacter ludwigii
MNRTKLVLGAVILGSTLLAGCSSNAKIDQLSSDVQTLNAKVDQLSNDVNAMR
SDVQAAKDDAARANQRLDNQATKYRK
>A0A2U2NVJ5|Enterobacter mori
MNRTKLVLGAVILGSTLLAGCSSNAKIDQLSSDVQTLNAKVDQLSNDVNAMR
SDVQAAKDDAARANQRLDNQATKYRK
>A0A4R0FWX0|Enterobacter quasihormaechei
MNRTKLVLGAVILGSTLLAGCSSNAKIDQLSSDVQTLNAKVDQLSNDVNAMR
SDVQAAKDDAARANQRLDNQATKYRK
>A0A0W2ICH8|Enterobacter roggenkampii
MNRTKLVLGAVILGSTLLAGCSSNAKIDQLSSDVQTLNAKVDQLSNDVNAMR
SDVQAAKDDAARANQRLDNQATKYRK
>A0A0F1BBT8|Enterobacter sichuanensis
MNRTKLVLGAVILGSTLLAGCSSNAKIDQLSSDVQTLNAKVDQLSNDVNAMR
SDVQAAKDDAARANQRLDNQATKYRK
>A0A198GNG7|Enterobacter soli
MNRTKLVLGAVILGSTLLAGCSSNAKIDQLSSDVQTLNAKVDQLSNDVNAMR
SDVQAAKDDAARANQRLDNQATKYRK
>A0A5A8ZU18|Enterobacter vonholyi
MNRTKLVLGAVILGSTLLAGCSSNAKIDQLSSDVQTLNAKVDQLSNDVNAIRS
DVQAAKDDAARANQRLDNQATKYRK
>A0A4R0G7H3|Enterobacter wuhouensis
MNRTKLVLGAVILGSTLLAGCSSNAKIDQLSSDVQTLNAKVDQLSNDVNAMR
SDVQAAKDDAARANQRLDNQATKYRK
>A0A135ICJ2|Enterovibrio coralii
MKIRLLTLAAIASSALLAGCSNNSALEQSVADLSSKVDSLSNQVSALQGDVAD
VAAASALSYDEAARANERIDNMAQSYTK
>A0A1T4VGP1|Enterovibrio nigricans
MKIRLLTLAAIASTAILAGCSNNSALEQSVADLSSKVDSLSNQVSALQGDVAD
VAAASALSYDEAARANERIDNMAQSYTK
>A0A2N7LH47|Enterovibrio norvegicus
MKMRLLTLAAIASSAVLVGCSGSSSLEQSVADLSSKVDSLSNQVSALQGDVSD
VAAASALSYDEAARANERIDNMAQSYTK
>A0A1C3EKM1|Enterovibrio pacificus
MKKRLLALAPLACCALLVGCSSSNGVEKSVAGLSAKVYQLSSQVDQLTEQLE
QVSEEAASLKGGIFDAKATSDLAYDEATRANERIDLMSQSIVKSQSYVK
>P02939|Erwinia amylovora
MNRTKLVLGAVILGSTLLAGCSSNAKIDQLSTDVQTLNAKVDQLSNDVTAIRS
DVQAAKDDAARANQRLDNQAHSYRK
>A0A4R6PBR7|Erwinia aphidicola
MNRTKLVLGAVILGSTLLAGCSSNAKIDQLSTDVQTLNAKVDQLSNDVNAIRS
DVQAAKDDAARANQRLDNQAHSYRK

>D8MRI2|Erwinia billingiae
MNRTKLVLGAVILGSTLLAGCSSNAKIDQLSTDVQTLNAKVDQLSNDVNAMR
SDVQAAKDDAARANQRLDNQAHSYRK
>A0A358L3Z1|Erwinia citreus
MNRTKLVLGAVILGSTLLAGCSSNAKIDQLSTDVQTLNAKVDQLSNDVNAIRS
DVQAAKDDAARANQRLDNQAHSYRK
>A0A0U5GM87|Erwinia gerundensis
MNRTKLVLGAVILGSTLLAGCSSNAKIDQLSTDVQTLNAKVDQLSNDVNAVR
SDVQAAKDDAARANQRLDNQAHSYRK
>A0A0L7T6E6|Erwinia iniecta
MNRTKLVLGAVILGSTLLAGCSSNAKIDQLSTDVQTLNAKVDQLSNDVNAVR
SDVQAAKDDAARANQRLDNQAHSYRK
>A0A014N944|Erwinia mallotivora
MNRTKLVLGAVILGSTLLAGCSSNAKIDQLSTDVQTLNAKVDQLSNDVNAMR
SDVQAAKDDAARANQRLDNQAHSYRK
>A0A3Q8HGP4|Erwinia persicina
MNRTKLVLGAVILASTMLAGCSSNAKIDQLSTDVQTLNAKVDQLSNDVNAIR
SDVQAAKDDAARANQRLDNQAHSYRK
>V5Z7F8|Erwinia piriflorinigrans
MNRTKLVLGAVILGSTLLAGCSSNAKIDQLSTDVQTLNAKVDQLSNDVTAIRS
DVQAAKDDAARANQRLDNQAHSYRK
>A0A3N6S0N0|Erwinia psidii
MNRTKLVLGAVILGSTLLAGCSSNAKIDQLSTDVQTLNAKVDQLSNDVNAMR
SDVQAAKDDAARANQRLDNQAHSYRK
>D2TBK3|Erwinia pyrifoliae
MNRTKLVLGAVILGSTLLAGCSSNAKIDQLSTDVQTLNAKVDQLSNDVTAIRS
DVQAAKDDAARANQRLDNQAHSYRK
>A0A4R7GNL8|Erwinia rhapontici
MNRTKLVLGAVILASTMLAGCSSNAKIDQLSTDVQTLNAKVDQLSNDVNAIR
SDVQAAKDDAARANQRLDNQAHSYRK
>B2VEN0|Erwinia tasmaniensis
MNRTKLVLGAVILGSTLLAGCSSNAKIDQLSTDVQTLNAKVDQLSNDVTAIRS
DVQAAKDDAARANQRLDNQAHSYRK
>A0A0M2K722|Erwinia tracheiphila
MNRTKLVLGAVILGSTLLAGCSSNAKIDQLSTDVQTLNAKVDQLSNDVNAIRS
DVQAAKDDAARANQRLDNQAHSYRK
>A0A0A3Z1T5|Erwinia typographi
MNRTKLVLGAVILASTMLAGCSSNAKIDQLSTDVQTLNAKVDQLSNDVNAIR
SDVQAAKDDAARANQRLDNQAHSYRK
>A0A6L6IN41|Escherichia alba
MNRTKLVLGAVILGSTLLAGCSSNAKIDQLSSDVQTLNAKVDQLSNDVNAMR
SDVQAAKDDAARANQRLDNQATKYRK

>A0A2T3RT11|Escherichia albertii
MKATKLVLGAVILGSTLLAGCSSNAKIDQLSSDVQTLNAKVDQLSNDVNAMR
SDVQAAKDDAARANQRLDNMATKYRK
>A0A4S5AZP9|Escherichia coli
MKATKLVLGAVILGSTLLAGCSSNAKIDQLSSDVQTLNAKVDQLSNDVNAMR
SDVQAAKDDAARANQRLDNMATKYRK
>A0A564UQ82|Escherichia fergusonii
MKATKLVLGAVILGSTLLAGCSSNAKIDQLSSDVQTLNAKVDQLSNDVNAMR
SDVQAAKDDAARANQRLDNMATKYRK
>A0A370V3B9|Escherichia marmotae
MKATKLVLGAVILGSTLLAGCSSNAKIDQLSSDVQTLNAKVDQLSNDVNAMR
SDVQAAKDDAARANQRLDNMATKYRK
>A0A2N0N0J1| Ewingella americana
MNRTKLVLGAVILASTMLAGCSSNAKIDQLSSDVSTLNSKVDQLSNDVNAIRS
DVQAAKDDAARANQRLDNQAHAYKK
>E1SS57|Ferrimonas balearica
MKAKLFIGLAMSALLFGCANTSELEAQVQSMSNKVDSLSDQVGALRADHSQ
MNSDIRAARAAAEDAQAEAARANARIDNMATGYRK
>A0A6H1UBP0|Ferrimonas lipolytica
MKVKLLAGLAMSTMLFGCANTADLEAQVSALSNKVDSLSTQVGSLSGQQDS
LAADIKATKSAAMDAQDEAARANSRIDAMASDYRKK
>A0A1M5NVC6|Ferrimonas marina
MKAKLLVGLAMSGLLFGCANTADLEAQVSELSNKVDDLSNQVGSLSAQHSE
MNSNINATKSAAMEAQQEAERANARIDNMASGYRKK
>A0A1G8UCA1|Ferrimonas sediminum
MKSKVLLVATAASALFLAGCANNDELAAQVSALSNKVDSFQAETQADQQRQ
DRDINAAKAAADDAAAEAMRANQRIDNIATSYKK
>A0A0J8VL66| Franconibacter pulveris
MNRTKLVLGAVILGSTLLAGCSSNAKIDQLSSDVQTLNAKVDQLSNDVNAMR
SDVQAAKDDAARANQRLDNQATKYRK
>A0A3N1PB44| Gallaecimonas pentaromativorans
MKRTLTLAALVVASTALFGCSSNTSSQSEMLSNKLDQLSSKVDKIASDQAMLK
QEVQDAKAAAEQASMEAKRANQRIDNMSSTYYKK
>K2IUG9| Gallaecimonas xiamenensis
MKRTVMLAALVLGSTALFGCTSNNMDSQAEMLSNKIDQLSSKVDQLASDQA
MMKKDIQDAKAAAESASMEARRANQRIDNMSSTYYKK
>Q39PY3|Geobacter metallireducens
MKGILLLIAMLLVLPVTMIGCATSGDLERVQADQRMLDAKVEQAVQDAQAA
KASADAAKAKADNATARADSAIKAAEERERIADEKAKKADATFQKSMRK
>A0A0C1TWT7|Geobacter soli
MKKRLSVISVTLVLAAALAGCATSREMEQVQADQRLLDAKIEQALQEAQAA
KASADAAKLEAQDATTRAENAEKAAQERERIADEKAKKADAVFQKSMRK

>A0A0D5N7N3|Geobacter sulfurreducens
MKTRLSVISVTLILAAALAGCATSREMEQVQADQRLLDAKIEQALQEAQAAK
AAADAAKLEAQDATTRAESAEKAAQERERLADEKAKKADAVFQKSMRK
>A0A250B6X2| Gibbsiella quercinecans
MNRTKLVLGAVILGSTLLAGCSSNAKIDQLSSDVQTLNAKVDQLSNDVNAIRS
DVQAAKDDAARANQRLDNQAHAYKK
>A0A128F5D3|Grimontia celer
MKIRLLAVAAIASSALLAGCSSKSGLEQSVADLSSKVDSLSNQVSALQGDVAD
VAAASALSYDEAARANERIDNMAQSYSK
>A0A377J844|Grimontia hollisae
MKIRLLALAAIASSAVLAGCSNNSALEQSVADLSSKVDSLSNQVSALQGDVAD
VASATNQAYDEAARANERIDNMAQSYTK
>R1J0X1|Grimontia indica
MKIRLLAVAAIASSALLAGCSSNSALEQSVADLSSKVDSLSNQVSALQGDVAD
VAAASALSYDEAARANERIDNMAQSYSK
>A0A128F2V5|Grimontia marina
MKIRLLAVAAIASSALLAGCSSNKALEQSVADLSSKVDSLSNQVSALQGDVAD
VAAASALAYDEAARANERIDNMAQSYSK
>A0A6M1RFM0|Grimontia sedimenti
MKIRLLAVAAIASSAVLAGCSSKSGLEQSVADLSSKVDSLSNQVSALQGDVAD
VAAASALSYDEAARANERIDNMAQSYSK
>A0A0K0HVN1|Hafnia alvei
MNRTKLVLGAVILASTMLAGCSSNAKIDQLSTDVQTLNTKVDQLSNDVNAM
RADVQTAKDDAARANQRLDNMATKYKK
>A0A2A2MEW9|Hafnia paralvei
MNRTKLVLGAVILASTMLAGCTSNAKIDQLSTDVQTLTTKVDQLSNDVNAMR
TDVQSAKDEAARANQRLDNIRGGYKK
>A0A1S8YR01| Izhakiella australiensis
MNRTKLVLGAVILGSTLLAGCSSNAKIDQLSTDVQTLNAKVDQLSNDVNAMR
SDVQAAKDDAARANQRLDNQAHSYRK
>A0A1I4V1T2| Izhakiella capsodis
MNRTKLVLGAVILGSTLLAGCSSNAKIDQLSTDVQTLNAKVDQLSNDVNAMR
SDVQAAKDDAARANQRLDNQAHSYRK
>A0A4P8YMH0| Jejubacter calystegiae
MNRTKLVLGAVILGSTLLAGCSSNAKIDQLSSDVQTLNAKVDQLSNDVNAMR
SDVQAAKDDAARANQRLDNQAHSYRK
>A0A411WJQ4| Jinshanibacter zhutongyuii
MNRTKVVLGAVILGATLLAGCAKSTDTSTNSKLTQLISDVAALRSDVQVAKDE
AARANQRLDNMTRSYKK
>A0A7Y8Y9Z1| Kalamiella piersonii
MNRTKLVLGAVILGSTLLAGCSSNAKIDQLSSDVQTLNAKVDQLSNDVNAIRS
DVQAAKDDAARANQRLDNQAHSYRK

>A0A094ZTE8|Klebsiella aerogenes
MNRTKLVLGAVILGSTLLAGCSSNAKIDQLSSDVQTLNAKVDQLSNDVNAMR
SDVQAAKDDAARANQRLDNQAHSYRK
>A0A7U2ZV27|Klebsiella africana
MNRTKLVLGAVILGSTLLAGCSSNAKIDQLSSDVQTLNAKVDQLSNDVNAMR
SDVQAAKDDAARANQRLDNQAHSYRK
>A0A285B574|Klebsiella grimontii
MNRTKLVLGAVILGSTLLAGCSSNAKIDQLSSDVQTLNAKVDQLSNDVNAMR
SDVQAAKDDAARANQRLDNAATKYRK
>A0A564KPN2|Klebsiella huaxiensis
MNRTKLVLGAVILGSTLLAGCSSNAKIDQLSSDVQTLNAKVDQLSNDVNAMR
SDVQAAKDDAARANQRLDNAATKYRK
>A0A5R9LNF4|Klebsiella indica
MNRTKLVLGAVILGSTLLAGCSSNAKIDQLSSDVQTLNAKVDQLSNDVNAMR
SDVQAAKDDAARANQRLDNQATKYRK
>A0A0G3PL07|Klebsiella michiganensis
MNRTKLVLGAVILGSTLLAGCSSNAKIDQLSSDVQTLNAKVDQLSNDVNAMR
SDVQAAKDDAARANQRLDNAATKYRK
>A0A0G3S7W6|Klebsiella oxytoca
MNRTKLVLGAVILGSTLLAGCSSNAKIDQLSSDVQTLNAKVDQLSNDVNAMR
SDVQAAKDDAARANQRLDNAATKYRK
>A0A0V9HIR3|Klebsiella pneumoniae
MNRTKLVLGAVILGSTLLAGCSSNAKIDQLSSDVQTLNAKVDQLSNDVNAMR
SDVQAAKDDAARANQRLDNQAHSYRK
>A0A1C1F567|Klebsiella quasipneumoniae
MNRTKLVLGAVILGSTLLAGCSSNAKIDQLSSDVQTLNAKVDQLSNDVNAMR
SDVQAAKDDAARANQRLDNQAHSYRK
>A0A087FLU7|Klebsiella variicola
MNRTKLVLGAVILGSTLLAGCSSNAKIDQLSSDVQTLNAKVDQLSNDVNAIRS
DVQAAKDDAARANQRLDNQAHSYRK
>A0A378GML0|Kluyvera ascorbata
MNRTKLVLGAVILGSTLLAGCSSNAKIDQLSSDVQTLNAKVDQLSNDVNAMR
SDVQAAKDDAARANQRLDNQAHSYRK
>A0A2X3DW47|Kluyvera cryocrescens
MNRTKLVLGAVILGSTLLAGCSSNAKIDQLSSDVQTLNAKVDQLSNDVNAVR
SDVQAAKDDAARANQRLDNQAHSYRK
>A0A1B7JXF1|Kluyvera georgiana
MNRTKLVLGAVILGSTLLAGCSSNAKIDQLSSDVQTLNAKVDQLSNDVNAMR
SDVQAAKDDAARANQRLDNQAHSYRK
>A0A1X0XAW1|Kluyvera intermedia
MNRTKLVLGAVILGSTLLAGCSSNAKIDQLSSDVQTLNAKVDQLSNDVNAIRS
DVQAAKDDAARANQRLDNQAHSYRK

>A0A1I6ZFC1|Kosakonia arachidis
MNRTKLVLGAVILGSTLLAGCSSNAKIDQLSSDVQTLNAKVDQLSNDVNAIRS
DVQAAKDDAARANQRLDNQAHSYRK
>A0A3R5YF41|Kosakonia cowanii
MNRTKLVLGAVILGSTLLAGCSSNAKIDQLSSDVQTLNAKVDQLSNDVNAVR
SDVQAAKDDAARANQRLDNQAHSYRK
>A0A1C4DA14|Kosakonia oryzendophytica
MNRTKLVLGAVILGSTLLAGCSSNAKIDQLSSDVQTLNAKVDQLSNDVNAIRS
DVQAAKDDAARANQRLDNQAHSYRK
>A0A1C4F8K3|Kosakonia oryziphila
MNRTKLVLGAVILGSTLLAGCSSNAKIDQLSSDVQTLNAKVDQLSNDVNAIRS
DVQAAKDDAARANQRLDNQAHSYRK
>A0A4R0H1B1|Kosakonia quasisacchari
MNRTKLVLGAVILGSTLLAGCSSNAKIDQLSSDVQTLNAKVDQLSNDVNAIRS
DVQAAKDDAARANQRLDNQAHSYRK
>A0A1T5HP61|Kosakonia radicincitans
MNRTKLVLGAVILGSTLLAGCSSNAKIDQLSSDVQTLNAKVDQLSNDVNAIRS
DVQAAKDDAARANQRLDNQAHSYRK
>A0A6P1QLG9|Kosakonia sacchari
MNRTKLVLGAVILGSTLLAGCSSNAKIDQLSSDVQTLNAKVDQLSNDVNAIRS
DVQAAKDDAARANQRLDNQAHSYRK
>A0A1V2Z6W3| Leclercia adecarboxylata
MNRTKLVLGAVILGSTLLAGCSSNAKIDQLSSDVQTLNAKVDQLSNDVNAMR
SDVQAAKDDAARANQRLDNQATKYRK
>A0A1A9FB36| Lelliottia amnigena
MNRTKLVLGAVILASTMLAGCSSNAKIDQLSSDVQTLNAKVDQLSNDVNAM
RSDVQAAKDDAARANQRLDNQATKYRK
>A0A1J5VUD1| Lelliottia nimipressuralis
MNRTKLVLGAVILGSTLLAGCSSNAKIDQLSSDVQTLNAKVDQLSNDVNAMR
SDVQAAKDDAARANQRLDNQATKYRK
>A0A2X4UYT7| Leminorella richardii
MNRTKVVLGAVILGATLLAGCAKSTDTATNSKLTQLISDVAALRSDVQVAKDE
AARANQRLDNMTRGYKK
>A0A2Y9TUZ3| Limnobaculum parvum
MNRTKVVLGAVILSATLLAGCAKSTDTSTNSKLTQLISDVAALRSDVQVAKDE
AARANQRLDNMTRSYKK
>A0A502L672| Litorilituus lipolyticus
MKKIAIVAFAAAVTGCSNTSALEENISTLTNKVDALSTQVANLEAQQKAISKD
AKAAKDAAEQALNDAKKANDRIDNVVSSYKK
>A0A4P6P7Z2| Litorilituus sediminis
MLIKKIAVIAVAAALTGCANNSALEENIASLTNKVDSLSNEVAQLKSQSQAISK
DAKAAKSAAEDALASAQKANDRIDNVVASYKK

>A0A1X3S3X1|Lonsdalea britannica
MNRTKLVLGAVILGSTLLAGCSSNAKIDQLSSDVQTLNSKVDQLSNDVTAIRS
DVQAAKDDAARANQRLDNQVRTYKK
>A0A1X3RYB2|Lonsdalea iberica
MNRTKLVLGAVILGSTLLAGCSSNAKIDQLSSDVQTLNSKVDQLSNDVTAIRS
DVQAAKDDAARANQRLDNQVRTYKK
>A0A3N0UVR9|Lonsdalea populi
MNRTKLVLGAVILGSTLLAGCSSNAKIDQLSSDVQTLNSKVDQLSNDVTAIRS
DVQAAKDDAARANQRLDNQVRTYKK
>A0A1H3YID4|Lonsdalea quercina
MNRTKLVLGAVILGSTLLAGCSSNAKIDQLSSDVQTLNSKVDQLSNDVTAIRS
DVQAAKDDAARANQRLDNQVRTYKK
>A0A1B7L6S1| Mangrovibacter phragmitis
MNRTKLVLGAVILGSTLLAGCSSNAKIDQLSSDVQTLNAKVDQLSNDVNAMR
SDVQAAKDDAARANQRLDNMAHSYRK
>A0A317PU99| Mangrovibacter plantisponsor
MNRTKLVLGAVILGSTLLAGCSSNAKIDQLSSDVQTLNAKVDQLSNDVNAMR
SDVQAAKDDAARANQRLDNMAHSYRK
>A0A6N3G5G1| Metakosakonia massiliensis
MNRTKLVLGAVILGSTLLAGCSSNAKIDQLSSDVQTLNAKVDQLSNDVNAIRS
DVQAAKDDAARANQRLDNQAHSYRK
>A0A654QX11|Mixta calida
MNRTKLVLGAVILGSTLLAGCSSNAKIDQLSSDVQTLNAKVDQLSNDVNAMR
SDVQAAKDDAARANQRLDNQAHSYRK
>A0A1X1DG15|Mixta gaviniae
MNRTKLVLGAVILGSTLLAGCSSNAKIDQLSSDVQTLNAKVDQLSNDVNAMR
SDVQAAKDDAARANQRLDNQAHSYRK
>A0A6P1PWG4|Mixta intestinalis
MNRTKLVLGAVILGSTLLAGCSSNAKIDQLSSDVQTLNAKVDQLSNDVNAMR
SDVQAAKDDAARANQRLDNQAHSYRK
>A0A506VF55|Mixta tenebrionis
MNRTKLVLGAVILGSTLLAGCSSNAKIDQLSSDVQTLNAKVDQLSNDVNAMR
SDVQAAKDDAARANQRLDNQAHSYRK
>A0A2K1QDQ4|Mixta theicola
MNRTKLVLGAVILGSTLLAGCSSNAKIDQLSSDVQTLNAKVDQLSNDVNAMR
SDVQAAKDDAARANQRLDNQAHSYRK
>A0A0H2QC23|Moellerella wisconsensis
MIRTKLLLGSIVLASALLAGCSNSTEVQKLSSDVQTLNGKVDQLSNDVQTLRT
DVQAAQQEATRANQRLDNQVRTYKK
>A0A0A5SKE3| Morganella morganii
MGRSKIVLGAVVLASALLAGCSSNAKFDQLDNDVKTLNAKVDQLSNDVNAI
RADVQQAKDEAARANQRLDNQVRSYKK

>A0A1B8HQB9|Morganella psychrotolerans
MSRSKIVLGAVILASALLAGCSSNAKFDQLDNDVKTLNAKVDQLSNDVNAIR
SDVQQAKDEAARANQRLDNQVRSYKK
>A0A5J6WHM0|Moritella marina
MMKKLLVAGVITTAVLLTGCANNKADLTDSVGGLNSEVSELTNQVQSLQSEQ
DALKSMVQSATDAAQKAQEEAERANMRIDNIAASYSK
>A0A090IDP0|Moritella viscosa
MNSKLLIAGLIVSSALIGGCANNADLQNSVGSLTSQVSKLTSQVKMLESEQGS
MKSGIGSNAAAAQQALEEAQRANSRIDNIAASYSK
>A0A330LLY1|Moritella yayanosii
MINQLLIAGAIMTSVLLTGCANNNSDLQNSVGSLNSEVSKLMAQVQQLESEH
GTMKTAIQSATDTAQQALEEAQRANSRIDNIAASYSK
>A0A418YHS4| Motilimonas pumilua
MKRKLMTVVGATLALGMVGCSNTAEIAELSDKVDMLTTEVSSLKDQQAVLA
GEVSDAKSAAMDASSEAMRANDRIDNIAGSYSK
>A0A4P8SH86| Nissabacter sp. SGAir0207
MNRTKLVLGAVILGSTLLAGCSSNAKIDQLSSDVQTLNAKVDQLSNDVNAIRS
DVQAAKDDAARANQRLDNQAHAYKK
>A0A4Q9D0G9| Obesumbacterium proteus
MNRTKLVLGAVILASTMLAGCSSNAKIDQLSTDVQTLNTKVDQLSNDVNAM
RADVQTAKDDAARANQRLDNMATKYKK
>A0A235CF85| Oceanimonas baumannii
MRNKMMMVGGVVGALMLAGCSSTTALEQKLDSLSQDVQAVQQGQQSNAA
KIDRLAADVAEARASADRANSRLDQMGRYTK
>A0A2P5TPY7| Oceanisphaera arctica
MRNKMMLVGGVVGTLLLAGCSSTDALETKMDSLAQDVQAVQQGQQMNSA
KIDRLAADVAEARASADRANSRLDQMGRYTK
>A0A1J4QFS7| Oceanisphaera psychrotolerans
MRNKMMLVGGVVGTLLLAGCSSTDALETKLDSLAQDVQAVQQGQQMNSAK
IDRLAADVAEARASADRANSRLDQMGRYTK
>A0A1W6B9J1|Pantoea alhagi
MNRTKLVLGAVILGSTLLAGCSSNAKIDQLSSDVQTLNAKVDQLSNDVNAMR
SDVQAAKDDAARANQRLDNQAHSYRK
>A0A2V2BFI7|Pantoea allii
MNRTKLVLGAVILGSTLLAGCSSNAKIDQLSSDVQTLNAKVDQLSNDVNAVR
SDVQAAKDDAARANQRLDNQAHSYRK
>A0A2V2A244|Pantoea ananas
MNRTKLVLGAVILGSTLLAGCSSNAKIDQLSSDVQTLNAKVDQLSNDVNAVR
SDVQAAKDDAARANQRLDNQAHSYRK
>A0A0H3KVS3|Pantoea ananatis
MNRTKLVLGAVILGSTLLAGCSSNAKIDQLSSDVQTLNAKVDQLSNDVNAVR
SDVQAAKDDAARANQRLDNQAHSYRK

>A0A506NPI1|Pantoea anthophila
MNRTKLVLGAVVLASTLLAGCSSNAKIDQLSSDVQTLNAKVDQLSNDVNAVR
SDVQAAKDDAARANQRLDNQAHSYRK
>A0A443IHA2| Pantoea beijingensis
MNRTKLVLGAVILGSTLLAGCSGNAKIDQLSTDVQTLNAKVDQLSNDVNAIR
SDVQAAKDDAARANQRLDNQARSYRK
>A0A653TPP8|Pantoea brenneri
MNRTKLVLGAVILGSTLLAGCSSNAKIDQLSSDVQTLNAKVDQLSNDVNAVR
SDVQAAKDDAARANQRLDNQAHSYRK
>A0A7Z9ET13|Pantoea calida
MNRTKLVLGAVILGSTLLAGCSSNAKIDQLSSDVQTLNAKVDQLSNDVNAMR
SDVQAAKDDAARANQRLDNQAHSYRK
>A0A2S9I616|Pantoea coffeiphila
MNRTKLVLGAVILGSTLLAGCSSNAKIDQLSSDVQTLNAKVDQLSNDVNAVR
SDVQAAKDDAARANQRLDNQAHSYRK
>A0A1X1BXZ9|Pantoea conspicua
MNRTKLVLGAVVLASTLLAGCSSNAKIDQLSSDVQTLNAKVDQLSNDVNAVR
SDVQAAKDDAARANQRLDNQAHSYRK
>A0A1X1ETT8|Pantoea cypripedii
MNRTKLVLGAVILGSTLLAGCSSNAKIDQLSSDVQTLNAKVDQLSNDVNAIRS
DVQAAKDDAARANQRLDNQAHSYRK
>A0A506QCK2|Pantoea deleyi
MNRTKLVLGAVVLASTMLAGCSSNAKIDQLSSDVQTLNAKVDQLSNDVNAV
RSDVQAAKDDAARANQRLDNQAHSYRK
>A0A437T2I2|Pantoea dispersa
MNRTKLVLGAVILGSTLLAGCSSNAKIDQLSSDVQTLNAKVDQLSNDVNAIRS
DVQAAKDDAARANQRLDNQAHSYRK
>A0A5Q2FS29|Pantoea eucalypti
MNRTKLVLGAVVLASTMLAGCSSNAKIDQLSSDVQTLNAKVDQLSNDVNAV
RSDVQAAKDDAARANQRLDNQAHSYRK
>A0A1V9DMJ7|Pantoea latae
MNRTKLVLGAVILGSTLLAGCSSNAKIDQLSSDVQTLNAKVDQLSNDVNAIRS
DVQAAKDDAARANQRLDNQAHSYRK
>A0A0B1R3P7|Pantoea rodasii
MNRTKLVLGAVILGSTLLAGCSSNAKIDQLSSDVQTLNAKVDQLSNDVNAIRS
DVQAAKDDAARANQRLDNQAHSYRK
>A0A1X1CSQ9|Pantoea rwandensis
MNRTKLVLGAVILGSTLLAGCSSNAKIDQLSSDVQTLNAKVDQLSNDVNAIRS
DVQAAKDDAARANQRLDNQAHSYRK
>A0A1M5R182|Pantoea sesami
MNRTKLVLGAVILGSTLLAGCSSNAKIDQLSSDVQTLNAKVDQLSNDVNAIRS
DVQAAKDDAARANQRLDNQATKYRK

>A0A6G6JJN2|Pantoea stewartii
MNRTKLVLGAVILGSTLLAGCSSNAKIDQLSSDVQTLNAKVDQLSNDVNAVR
SDVQAAKDDAARANQRLDNQAHSYRK
>A0A7Z1Y3K3|Pantoea vagans
MNRTKLVLGAVVLASTLLAGCSSNAKIDQLSSDVQTLNAKVDQLSNDVNAVR
SDVQAAKDDAARANQRLDNQAHSYRK
>A0A1X1DBQ4|Pantoea wallisii
MNRTKLVLGAVILGSTLLAGCSSNAKIDQLSSDVQTLNAKVDQLSNDVNAIRS
DVQAAKDDAARANQRLDNQAHSYRK
>A0A3L8Q270|Parashewanella curva
MATYGHKLLGSVRTMKKIFTIAVISLPLFLAGCATDAALQDHIHATNQKLDLLS
SQISDLQSQHDQLMNSIKHNQSASERANDRLDNLATHYKK
>A0A3A6UFW9|Parashewanella spongiae
MNKKVLTIAGLALTALLGGCANTAALEESVANLGNKVDQLSAEVSSLKSEQG
QLASTVRSAKSAAMDAKAEAKRANDRLDNMATRYKK
>A0A1V2R4C1|Pectobacterium actinidiae
MNRTKLVLGAVILGSTLLAGCSSNAKIDQLSSDVQTLNAKVDQLSNDVNAIRS
DVQAAKDDAARANQRLDNQVRTYKK
>A0A3R8QJF0|Pectobacterium aquaticum
MNRTKLVLGAVILGSTLLAGCSSNAKIDQLSSDVQTLNAKVDQLSNDVNAIRS
DVQAAKDDAARANQRLDNQVRTYKK
>A0A7W2E1Y6|Pectobacterium aroidearum
MNRTKLVLGAVILGSTLLAGCSSNAKIDQLSSDVQTLNAKVDQLSNDVNAIRS
DVQAAKDDAARANQRLDNQVRTYKK
>Q6D622|Pectobacterium atrosepticum
MNRTKLVLGAVILGSTLLAGCSSNAKIDQLSSDVQTLNAKVDQLSNDVNAIRS
DVQAAKDDAARANQRLDNQVRTYKK
>A0A093SCF9|Pectobacterium betavasculorum
MNRTKLVLGAVILGSTLLAGCSSNAKIDQLSSDVQTLNAKVDQLSNDVNAIRS
DVQAAKDDAARANQRLDNQVRTYKK
>A0A086EV21|Pectobacterium brasiliense
MNRTKLVLGAVILGSTLLAGCSSNAKIDQLSSDVQTLNAKVDQLSNDVNAIRS
DVQAAKDDAARANQRLDNQVRTYKK
>A0A419B0J1|Pectobacterium carotovorum
MNRTKLVLGAVILGSTLLAGCSSNAKIDQLSSDVQTLNAKVDQLSNDVNAIRS
DVQAAKDDAARANQRLDNQVRTYKK
>A0A7V8IHA1|Pectobacterium fontis
MNRTKLVLGAVILGSTLLAGCSSNAKIDQLSSDVQTLNAKVDQLSNDVNAIRS
DVQAAKDDAARANQRLDNQVRTYKK
>A0A094STP9|Pectobacterium odoriferum
MNRTKLVLGAVILGSTLLAGCSSNAKIDQLSSDVQTLNAKVDQLSNDVNAIRS
DVQAAKDDAARANQRLDNQVRTYKK

>A0A0H3IA64|Pectobacterium parmentieri
MNRTKLVLGAVILGSTLLAGCSSNAKIDQLSSDVQTLNAKVDQLSNDVNAIRS
DVQAAKDDAARANQRLDNQVRTYKK
>A0A0J5XYY1|Pectobacterium peruviense
MNRTKLVLGAVILGSTLLAGCSSNAKIDQLSSDVQTLNAKVDQLSNDVNAIRS
DVQAAKDDAARANQRLDNQVRTYKK
>A0A093RDD7|Pectobacterium polaris
MNRTKLVLGAVILGSTLLAGCSSNAKIDQLSSDVQTLNAKVDQLSNDVNAIRS
DVQAAKDDAARANQRLDNQVRTYKK
>A0A4U7KAB6|Pectobacterium polonicum
MNRTKLVLGAVILGSTLLAGCSSNAKIDQLSSDVQTLNAKVDQLSNDVNAIRS
DVQAAKDDAARANQRLDNQVRTYKK
>A0A2T3W2K8|Pectobacterium punjabense
MNRTKLVLGAVILGSTLLAGCSSNAKIDQLSSDVQTLNAKVDQLSNDVNAIRS
DVQAAKDDAARANQRLDNQVRTYKK
>A0A221TAU8|Pectobacterium versatile
MNRTKLVLGAVILGSTLLAGCSSNAKIDQLSSDVQTLNAKVDQLSNDVNAIRS
DVQAAKDDAARANQRLDNQVRTYKK
>A0A1D7Z7F0|Pectobacterium wasabiae
MNRTKLVLGAVILGSTLLAGCSSNAKIDQLSSDVQTLNAKVDQLSNDVNAIRS
DVQAAKDDAARANQRLDNQVRTYKK
>A1ALI3| Pelobacter propionicus
MKKSLLLIATMLVLPVTFMGCATSGDLEKMQAQQKLIDAKADQALQDAQAA
KTAADAAKVKADDATLRAENAEKAAQERERIADEKAKKADAVFQKSMKK
>A0A1Y6MDI7|Photobacterium andalusiense
MKRTVSILAGVLLSASLVGCSSSDEVSQMQQLTNKVDTLSEQVSALQGQQDQ
IVGAVNDSRAASDAAYQEAMRANKRIDNIAGSYTK
>A0A0D8MQ19|Photobacterium angustum
MKRTVTILAGLLLSASLVGCSSTDADQMQTLTNKVDTLAEQVSALQGQQDQI
VSAVNDSRAASDAAYQEAMRANKRIDNIAGSYTK
>A0A090QJP1|Photobacterium aphoticum
MKRSIALATGLILSTTLMGCSSTEQAEQMTQLSNKIDTLSEQVSTLQTQQDAM
AGAVNDALTASDAAYQEAMRANQRIDNVAHSYKK
>A0A0J1H0Y9|Photobacterium aquae
MKRSAMLFAGLVLSASLMGCSNSQEAEQLSQLTNKVDALTDQVAAMQVQQD
NIAGAVNETSAAADAAYQEAMRANQRIDNVATSYRK
>A0A1Y6L051|Photobacterium aquimaris
MKRTVSILAGVLLSASLVGCSSSDEVSQMQQLTNKVDTLSQQVSALQGQQDQ
IVGAVNDSRAASDAAYQEAMRANKRIDHIAGSYTK
>A0A2N4UVP4|Photobacterium carnosum
MKRTVSILAGVLLSASLVGCSSSDEVSQMQQLTNKVDTLSTQVSALQGQQDQ
IVGAVNDSRAASDAAYQEAMRANKRIDNIAGSYTK

>A0A3S3QRY0|Photobacterium chitinilyticum
MNRSLSILTGIILSATLIGCSSSDEVDQMQQLTNKVDMLSDQVGALQSQQDQM
AGAVNDARAASDAAYQEAMRANQRIDNIASSYSK
>A0A2T3IDP7|Photobacterium damselae
MNRKVTILAGVILSATLMGCSSSSEMQQLTNKVDALSDQVSALQSQQDQLAG
AVNDARAASDAAYQEAMRANQRIDNIRGSYTK
>A0A2T3JDB2|Photobacterium frigidiphilum
MNRSLTILSGVILSAALMGCSSSDEVDQMQQLTNKVDMLSDQVSALQSQQDQ
MAGAVNDSRAASDAAYQEAMRANQRIDNIAGSYTK
>A0A0B9GYX9|Photobacterium gaetbulicola
MNRSLSILTGIILSATLIGCSSSDEVDQMQQLTNKVDMLSDQVGALQSQQDQM
AGAVNDARAASDAAYQEAMRANQRIDNIASSYSK
>A0A0J1K0R4|Photobacterium ganghwense
MNRSLSILTGIILSATLIGCSSSDEVDQMQQLTNKVDMLSDQVGALQSQQDQM
AGAVNDARAAADAAYQEAMRANQRIDNIASSYRK
>A0A0F5VB75|Photobacterium halotolerans
MNRSLSILTGIILSATLIGCSSSDEVDQMQQLTNKVDMLSDQVGALQSQQDQM
AGAVNDARASADAAYQEAMRANQRIDNMASSYRK
>A0A0D8P4E9|Photobacterium iliopiscarium
MKRTVSILAGVLLSASLVGCSSSDEVSQMQQLTNKVDTMAEQVSALQGQQD
QIVGAVNDSRAASDAAYQEAMRANKRIDNIAGSYTK
>A0A2T3L4A2|Photobacterium indicum
MNRSLTILSGVILSAALMGCSSSDEVDQMQQLTNKVDMLSDQVSALQSQQDQ
MAGAVNDSRAASDAAYQEAMRANQRIDNIAGSYTK
>A0A178K7J4|Photobacterium jeanii
MNRSLPILAGIILSAALVGCSNSDKPDAMQQLTNKVDMLSDQVSALQSQQDQ
MAGAVNDAKASSDAAYQEAMRANQRIDNMASSYTK
>A0A2T3KF56|Photobacterium kishitanii
MKRTVSILAGVLLSASLVGCSSSDEVSQMQQLTNKVDTLSEQVSALQGQQDQ
IVGAVNDSRAASDAAYQEAMRANKRIDNIAGSYTK
>A0A2G4WRN9|Photobacterium leiognathi
MKRTVSILAGVLLSASLVGCSSTDADQMQQLTNKVDTLAEQVSALQGQQDQI
VSAVNDSRAASDAAYQEAMRANKRIDNIAGSYTK
>A0A2T3MZE9|Photobacterium lipolyticum
MKRSLSILAGLILSASLIGCSSSDEVDQMQQLTNKVDMLSDQVSALQSQQDQ
MAGAVNDSRAASDAAYQEAMRANQRIDNIASSYSK
>A0A2T3J3Y5|Photobacterium lutimaris
MNRSLSILTGIILSATLIGCSSSDEVDQMQQLTNKVDMLSDQVGALQSQQDQM
AGAVNDARAASDAAYQEAMRANQRIDNIASSYSK
>A0A1Y6MDH2|Photobacterium malacitanum
MKRTVSILAGVLLSASLVGCSSSDEVSQMQQLTNKVDTLSEQVSALQGQQDQ
IVGAVNDSRAASDAAYQEAMRANKRIDNIAGSYTK

>L8JDT7|Photobacterium marinum
MNRSLSILTGIILSATLMGCSSSEQAEQLQQLTNKVDMLSDQVGALQSQQDQM
AGAVNDSRAAADAAYQEAMRANQRIDNIASSYSK
>A0A2T3JTU6|Photobacterium phosphoreum
MKRTVSILAGVLLSASLVGCSSSDEVSQMQQLTNKVDTLSSQVSALQGQQDQI
VGAVNDSRAASDAAYQEAMRANKRIDHIAGSYTK
>A0A1T5HWX3|Photobacterium piscicola
MKRTVSILAGVLLSASLVGCSSSDEVSQMQQLTNKVDTLSEQVSALQGQQDQ
IVGAVNDSRAASDAAYQEAMRANKRIDHIAGSYTK
>Q6LJB4|Photobacterium profundum
MNRSLTILSGVILSAALMGCSSSDEVDQMQQLTNKVDMLSDQVSALQSQQDQ
MAGAVNDSRAASDAAYQEAMRANQRIDNIAGSYTK
>A0A1Q9H1A1|Photobacterium proteolyticum
MNRSLSILTGIILSATLIGCSSSDEVDQMQQLTNKVDMLSDQVGALQSQQDQM
AGAVNDARAASDAAYQEAMRANQRIDNIASSYSK
>A0A2T3NMS1|Photobacterium rosenbergii
MNRSLSILTGIILSATLIGCSSSDEVDQMQQLTNKVDMLSDQVGALQSQQDQM
AGAVNDARAASDAAYQEAMRANQRIDNIASSYSK
>A0A2T3NUP6|Photobacterium sanctipauli
MNRSLSILTGIILSATLIGCSSSDEVDQMQQLTNKVDMLSDQVGALQSQQDQM
AGAVNDARAASDAAYQEAMRANQRIDNIASSYSK
>A0A264TCS4|Photobacterium sanguinicancri
MNRSLPILAGIILSAALVGCSSSDEPDAMQQLTNKVDMLSDQVGALQSQQDQ
MAGAVNDAKASSDAAYQEAMRANQRIDNMASSYTK
>A0A0J8XY86|Photobacterium swingsii
MNRSLPILAGIILSAALVGCSSSDEPDAMQQLTNKVDMLSDQVGALQSQQDQ
MAGAVNDAKASSDAAYQEAMRANQRIDNMASSYTK
>A0A1T4RIF6|Photobacterium toruni
MKRTVSILAGVLLSASLVGCSSSDEVSQMQQLTNKVDTLSSQVSALQGQQDQI
VGAVNDSRAASDAAYQEAMRANKRIDNIAGSYTK
>C7BHQ9| Photorhabdus asymbiotica subsp. asymbiotica
MNRTKIVLGAVVLASTLLAGCSSTAKVDQLSSDIQTLNAKVDQLSNDVNSVR
TDIQAAKDEAARANQRLDNQVRSYKK
>A0A1C0U3V0|Photorhabdus australis
MNRTKIVLGAVVLASTMLAGCSSTAKVDQLSSDIQTLNAKVDQLSNDVNSVR
TDIQAAKDEAARANQRLDNQVRSYKK
>A0A329XCL2|Photorhabdus bodei
MNRTKIVLGAVVLASTLLAGCSSTAKVDQLTSDIQTLNAKVDQLSNDVNAVR
TDIQAAKDDAARANQRLDNQVRSYKK
>A0A7X5QBH8|Photorhabdus cinerea
MNRTKIVLGAVVLASTMLAGCSSTAKVDQLSSDIQTLNAKVDQLSNDVNSVR
TDIQAAKDEAARANQRLDNQVRSYKK

>A0A5B0WK65|Photorhabdus heterorhabditis
MNRTKIVLGAVVLASTMLAGCSSTAKVDQLSSDIQTLNAKVDQLSNDVNSVR
TDIQAAKDEAARANQRLDNQVRSYKK
>A0A7X5H343|Photorhabdus kayaii
MNRTKIVLGAVVLASTLLAGCSSTAKVDQLTSDIQTLNAKVDQLSNDVNAVR
TDIQAAKDDAARANQRLDNQVRSYKK
>A0A7C9KC06|Photorhabdus khanii
MNRTKIVLGAVVLASTLLAGCSSTAKVDQLSSDVQTLNAKVDQLSNDVNAVR
TDIQAAKDDAARANQRLDNQVRSYKK
>A0A329VI23|Photorhabdus laumondii subsp. clarkei
MNRTKIVLGAVVLASTLLAGCSSTAKVDQLTSDIQTLNAKVDQLSNDVNAVR
TDVQAAKDDAARANQRLDNQVRSYKK
>A0A0A0CV80|Photorhabdus luminescens
MNRTKIVLGAVVLASTLLAGCSSTAKVDQLTSDIQTLNAKVDQLSNDVNAVR
TDIQAAKDDAARANQRLDNQVRSYKK
>A0A1B8YEM3|Photorhabdus namnaonensis
MNRTKIVLGAVVLASTLLAGCSSTAKVDQLTSDIQTLNAKVDQLSNDVNAVR
TDIQAAKDDAARANQRLDNQVRSYKK
>A0A7X5TL11|Photorhabdus stackebrandtii
MNRTKIVLGAVVLASTLLAGCSSTAKVDQLSSDVQTLNAKVDQLSNDVNAVR
TDIQAAKDDAARANQRLDNQVRSYKK
>A0A4R7Q0Z5|Photorhabdus temperata
MNRTKIVLGAVVLASTLLAGCSSTAKVDQLSSDVQTLNSKVDQLSNDVNAVR
TDIQAAKDDAARANQRLDNQVRSYKK
>A0A0F7LT53|Photorhabdus thracensis
MNRTKIVLGAVVLASTLLAGCSSTAKVDQLSSDVQTLNAKVDQLSNDVNAVR
TDIQAAKDDAARANQRLDNQVRSYKK
>A0A2S9J9L2| Phyllobacterium myrsinacearum
MKATKLVLGAVILGSTLLAGCSSNAKIDQLSSDVQTLNAKVDQLSNDVNAMR
SDVQAAKDDAARANQRLDNMATKYRK
>A0A0G3Q7G0| Phytobacter ursingii
MNRTKLVLGAVILGSTLLAGCSSNAKIDQLSSDVQTLNAKVDQLSNDVNAIRS
DVQAAKDDAARANQRLDNQAHSYRK
>A0A1A9AW70| Plesiomonas shigelloides
MNKTKLILGTIVLSSTLLAGCANTKLEQSVADLNTKVDQLATDVGSLRTDVQ
DAKAEAARANQRLDNMATSYKK
>A0A089PLQ9| Pluralibacter gergoviae
MNRTKLVLGAVILGSTLLAGCSSNAKIDQLSSDVQTLNAKVDQLSNDVNAMR
SDVQAAKDDAARANQRLDNQAHSYRK
>A0A0G3CT06| Pragia fontium
MNRTKVVLGAVILGATLLAGCAKSTDTATNSKLTQLISDVAALRSDVQVAKDE
AARANQRLDNMTRSYKK

>A0A6I7D414| Proteus columbae
MKAKLVLGAVILASGLLAGCSSSNNAQLDQISSDVSRLNTQVQQLSGDVQSA
RAEAKSAYDEAARANQRLDNQVTTYKK
>A0A410XD84| Proteus hauseri
MKAKLVLGAVIVASSLLAGCSSNNAQLDQISSDVSRLNTQVQQLSGDVQSAR
AEAKSAYDEAARANQRLDNQVTTYKK
>A0A1Z1SVX2| Proteus mirabilis
MKAKIVLGAVILASGLLAGCSSSNNAQLDQISSDVNRLNTQVQQLSSDVQSA
NAQAKAAYDEAARANQRLDNQVTTYKK
>A0A198F9E9| Proteus myxofaciens
MKAKIVLGAVILASGLLAGCSSNNNAQLDQISSDVNRLNTQVQQLSSDVQSA
RSEAKSAYDEATRANQRLDNQVKTYKK
>A0A0G4PYX8| Proteus penneri
MKAKLVLGAVILASGLLAGCSSSNNAQLDQISSDVSRLNTQVQQLSSDVQSA
RAEAKSAYDEAARANQRLDNQVTTYKK
>A0A6I6FWF0|Proteus terrae
MKAKLVLGAVILASGLLAGCSSSNNAQLDQISSDVSRLNTQVQQLSGDVQSA
RAEAKSAYDEAARANQRLDNQVTTYKK
>A0A6M8R7L9| Proteus vulgaris
MKAKLVLGAVILASGLLAGCSSSNNAQLDQISSDVSRLNTQVQQLSGDVQSA
RAEAKSAYDEAARANQRLDNQVTTYKK
>A0A291EEZ5|Providencia alcalifaciens
MIRTKIVLGSIVLASALLAGCSNSTEVQKLSSDVQTLNGKVDQLSNDVQSLRS
DVQTAQEEAARANQRLDNQVRTYKK
>K8WYB4|Providencia burhodogranariea
MIRTKFVLGSIVLASALLAGCSNSTEVQKLSSDVQTLNGKVDQLSSDVQTIRA
EVQTAQQEAARANQRLDNQVRSYKK
>A0A4D7J6H6|Providencia heimbachae
MIRTKIVLGSIVLASALLAGCSNSTEVQKLSSDVQTLNGKVDQLSNDVQSLRS
DVQTAQEEAARANQRLDNQVRTYKK
>A0A345LUR6|Providencia huaxiensis
MIRTKIVLGSIVLASALLAGCSNSTEVQKLSSDVQTLNGKVDQLSNDVQSLRS
DVQTAQEEAARANQRLDNQVRTYKK
>A0A1B8SRD2|Providencia rettgeri
MIRTKIVLGSIVLASALLAGCSNSTEVQKLSSDVQTLNGKVDQLSNDVQSLRS
DVQTAQEEAARANQRLDNQVRTYKK
>A0A379G3B8|Providencia rustigianii
MIRTKIVLGSIVLASALLAGCSNSTEVQKLSSDVQTLNGKVDQLSNDVQSLRS
DVQTAQEEAARANQRLDNQVRTYKK
>K8WIU4|Providencia sneebia
MIRTKIVLGSIVLASALLAGCSTNNSTEIQRLSSDVQTLNGKVDQLSSDVQTIR
AEVQTAQQEAARANQRLDNQVRSYKK

>A0A379GPW5|Providencia stuartii
MIRTKIVLGSIVLASALLAGCSNSTEVQKLSSDVQTLNGKVDQLSNDVQTIRA
EVQAAQQEAARANQRLDNQVRSYKK
>A0A7T8D474|Providencia vermicola
MIRTKIVLGSIVLASALLAGCSSNSTEVQKLSSDVQTLNGKVDQLSNDVQTIR
AEVQTAQQEAARANQRLDNQVRSYKK
>A0A090V9N1| Pseudescherichia vulneris
MNRTKLVLGAVILGSTLLAGCSSNAKIDQLSSDVQTLNAKVDQLSNDVNAIRS
DVQAAKDDAARANQRLDNQAHSYRK
>A0A244CNC1| Pseudoalteromonas ulvae
MFKKVAPFVALGVLVSLSGCSNTSGLEEQVKSLNAKVDSLTMKVDGLSSDVA
SANSMASQNNSDIKMLKSSIDSAQADASKANDRLDNLVSSYKK
>A0A366FLQ8| Pseudocitrobacter faecalis
MNRTKLVLGAVILGSTLLAGCSSNAKIDQLSSDVQTLNAKVDQLSNDVNAIRS
DVQAAKDDAARANQRLDNQAHSYRK
>A1SXX7| Psychromonas ingrahamii
MNKKLLITGAVASIVLLAGCSNHDDMNESMSNLTNQVSELSAKVDMVADDQ
ASMKADLATVAGEAERANSRIDNIATSYKK
>H2IT70| Rahnella aquatilis
MNRTKLVLGAVILASTMLAGCSSNAKIDQLSSDVSTLNSKVDQLSNDVNAIRS
DVQAAKDDAARANQRLDNQAHAYKK
>A0A419MH61| Rahnella inusitata
MNRTKLVLGAVILASTMLAGCSSNAKIDQLSSDVSTLNSKVDQLSNDVNAMR
SDVQAAKDDAARANQRLDNQAHAYKK
>A0A419NC16| Rahnella woolbedingensis
MNRTKLVLGAVILASTMLAGCSSNAKIDQLSSDVSTLNSKVDQLSNDVNAMR
SDVQAAKDDAARANQRLDNQAHAYKK
>A0A514EQJ3|Raoultella electrica
MNRTKLVLGAVILGSTLLAGCSSNAKIDQLSSDVQTLNAKVDQLSNDVNAMR
SDVQAAKDDAARANQRLDNQATKYRK
>A0A038CW44|Raoultella ornithinolytica
MNRTKLVLGAVILGSTLLAGCSSNAKIDQLSSDVQTLNAKVDQLSNDVNAMR
SDVQAAKDDAARANQRLDNQATKYRK
>A0A2X2EG12|Raoultella planticola
MNRTKLVLGAVILGSTLLAGCSSNAKIDQLSSDVQTLNAKVDQLSNDVNAMR
SDVQAAKDDAARANQRLDNQATKYRK
>A0A1V2BGS9|Raoultella terrigena
MNRTKLVLGAVILGSTLLAGCSSNAKIDQLSSDVQTLNAKVDQLSNDVNAMR
SDVQAAKDDAARANQRLDNQATKYRK
>D4G7U1|Riesia pediculicola
MKNKFILCFLTLIFLTLSGCTTNNQTKKISSDLNSLKDKVENISEETQSIQTDLQ
NTKEEAVRANQRLDNQVHKYKK

>A0A1H9GF69| Rosenbergiella nectarea
MNRTKLVLGAVILGSTLLAGCSSNAKIDQLSSDVQTLNTKVDQLSTDLNSVRT
DVQAAKDDAARANQRLDNQAHSYRK
>A0A1X0WEM6|Rouxiella badensis
MNRTKLVLGAVILGSTLLAGCSSNAKIDQLSSDVSTLNSKVDQLSNDVAAVKS
DVQAAKDDAARANQRLDNQAHAYKK
>A0A1X0W4U6|Rouxiella silvae
MNRTKLVLGAVILASTLLAGCSSNAKIDQLSSDVSTLNSKVDQLSNDVAAVKA
DTQAAKDDAARANQRLDNQAHAYKK
>A0A662ZKG9| Ruminobacter amylophilus
MNKLQVVLGTVALGAALVGCSDTSALNQKVDALSNKVEALSNDVEALKSSQ
AQTASKAQAAYDEAVRANERLDNLSNKYKK
>A0A6G9PZV0|Salinivibrio costicola
MKIRPMALAAIASTVLLVGCTSNTALQQSVDDLSAKVDALSNDVSMMKNDS
QMAYDEAKRANERIDNMAQSYTK
>A0A1V3GJL5|Salinivibrio kushneri
MKIRPMALAAIASTVLLVGCTSNTALQQSVDDLSAKVDALSNDVSMMKSDV
SDTKDTSQMAYDEAKRANKRIDNMAQSYTK
>A0A1V3GYV2|Salinivibrio sharmensis
MKIRPMALAAIASSMLLVGCTSNTALQQSVDDLSAKVDALSNDVSMMKSDV
SDTKDTSQMAYDEAKRANKRIDNMAQSYTK
>A0A5I1FMI6|Salmonella abony
MNRTNQLILGAVVLGSTLLAGCSSNAKIDQLSSDVQTLSAKVEQLSNDVNAM
RSDVQAAKDDAARANQRLDNKVVSHVRK
>A0A738DWZ0|Salmonella abortus-equi
MNRTNQLILGAVVLGSTLLAGCSSNAKIDQLSSDVQTLSAKVEQLSNDVNAM
RSDVQAAKDDAARANQRLDNKVVSHVRK
>B5F7C0|Salmonella agona
MNRTKLVLGAVILGSTLLAGCSSNAKIDQLSSDVQTLNAKVDQLSNDVNAMR
SDVQAAKDDAARANQRLDNQATKYRK
>A0A2T8MGS4|Salmonella anatum
MNRTKLVLGAVILGSTLLAGCSSNAKIDQLSSDVQTLNAKVDQLSNDVNAMR
SDVQAAKDDAARANQRLDNQATKYRK
>A9MEP0|Salmonella arizonae
MNRTKLVLGAVILGSTLLAGCSSNAKIDQLSSDVQTLNAKVDQLSNDVNAMR
SDVQAAKDDAARANQRLDNQATKYRK
>A0A3V6R8P5|Salmonella berta
MNRTKLVLGAVILGSTLLAGCSSNAKIDQLSSDVQTLNAKVDQLSNDVNAMR
SDVQAAKDDAARANQRLDNQATKYRK
>A0A5I2K3D6|Salmonella blockley
MNRTKLVLGAVILGSTLLAGCSSNAKIDQLSSDVQTLNAKVDQLSNDVNAMR
SDVQAAKDDAARANQRLDNQATKYRK

>A0A3S4EWT2|Salmonella bongori
MNRTKLVLGAVILGSTLLAGCSSNAKIDQLSSDVQTLNAKVDQLSNDVNAMR
SDVQAAKDDAARANQRLDNQATKYRK
>Q57PQ7|Salmonella choleraesuis
MNRTKLVLGAVILGSTLLAGCSSNAKIDQLSSDVQTLNAKVDQLSNDVNAMR
SDVQAAKDDAARANQRLDNQATKYRK
>A0A3W0AMP5|Salmonella derby
MNRTKLVLGAVILGSTLLAGCSSNAKIDQLSSDVQTLNAKVDQLSNDVNAMR
SDVQAAKDDAARANQRLDNQATKYRK
>A0A2I5HHC4|Salmonella diarizonae
MNRTKLVLGAVILGSTLLAGCSSNAKIDQLSSDVQTLNAKVDQLSNDVNAMR
SDVQAAKDDAARANQRLDNQATKYRK
>A0A3T3IIE3|Salmonella dublin
MNRTKLVLGAVILGSTLLAGCSSNAKIDQLSSDVQTLNAKVDQLSNDVNAMR
SDVQAAKDDAARANQRLDNQATKYRK
>A0A2T8T5P0|Salmonella enterica
MNRTNKLILGAVVLGSALLAGCSSNAKIDQLSSDVQTLSAKVDQLSNDVNAM
RSDVQAAKDDAARANQRLDNKVSRVRK
>A0A372N9P6|Salmonella enteritidis
MNRTNQLILGAVVLGSTLLAGCSSNAKIDQLSSDVQTLSAKVEQLSNDVNAM
RSDVQAAKDDAARENQRLDNKVVSHVRK
>A0A3V9NLV2|Salmonella gallinarum
MNRTNQLILGAVVLGSTLLAGCSSNAKIDQLSSDVQTLSAKVEQLSNDVNAM
RSDVQAAKDDAARENQRLDNKVVSHVRK
>A0A3V2VYT3|Salmonella hadar
MNRTNQLILGAVVLGSTLLAGCSSNAKIDQLSSDVQTLSAKVEQLSNDVNAM
RSDVQAAKDDAARANQRLDNKVVSHVRK
>A0A6C6ZIR7|Salmonella heidelberg
MNRTKLVLGAVILGSTLLAGCSSNAKIDQLSSDVQTLNAKVDQLSNDVNAMR
SDVQAAKDDAARANQRLDNQATKYRK
>A0A702LD96|Salmonella houtenae
MNRTKLVLGAVILGSTLLAGCSSNAKIDQLSSDVQTLNAKVDQLSNDVNAMR
SDVQAAKDDAARANQRLDNQATKYRK
>A0A637QEJ5|Salmonella infantis
MNRTNQLILGAVVLGSTLLAGCSNNAKIDQLSSDVQTLSAKVEQLSNDVNAM
RSDVQAAKDDAARENQRLDNKVVSHVRK
>A0A3A3NKH3|Salmonella montevideo
MNRTKLVLGAVILGSTLLAGCSSNAKIDQLSSDVQTLNAKVDQLSNDVNAMR
SDVQAAKDDAARANQRLDNQATKYRK
>A0A5H7LFY6|Salmonella moscow
MNRTKLVLGAVILGSTLLAGCSSNAKIDQLSSDVQTLNAKVDQLSNDVNAMR
SDVQAAKDDAARANQRLDNQATKYRK

>A0A3T3EM33|Salmonella muenchen
MNRTKLVLGAVILGSTLLAGCSSNAKIDQLSSDVQTLNAKVDQLSNDVNAMR
SDVQAAKDDAARANQRLDNQATKYRK
>A0A5H9DV60|Salmonella muenster
MNRTNRLILGAVVLGSTLLAGCASNAKIDQLSSDVQTLSAKVEQLSNDVNAM
RSDVQAAKDDAARANQRLDNKVVSHVRK
>A0A0R9MSH0|Salmonella newport
MNRTKLVLGAVILGSTLLAGCSSNAKIDQLSSDVQTLNAKVDQLSNDVNAMR
SDVQAAKDDAARANQRLDNQATKYRK
>A0A4S3DSE8|Salmonella oranienberg
MNRTKLVLGAVILGSTLLAGCSSNAKIDQLSSDVQTLNAKVDQLSNDVNAMR
SDVQAAKDDAARANQRLDNQATKYRK
>A0A5V6VQN4|Salmonella ordonez
MNRTKLVLGAVILGSTLLAGCSSNAKIDQLSSDVQTLNAKVDQLSNDVNAMR
SDVQAAKDDAARANQRLDNQATKYRK
>A0A5I6RT00|Salmonella potsdam
MNRTNQLILGAVVLGSTLLAGCSSNAKIDQLSSDVQTLSAKVDQLSNDVNAM
RSDVQAAKDDAARANQRLDNKVVSHVRK
>A0A5W9CJ55|Salmonella rubislaw
MNRTNKLILGAVVLGSALLAGCSSNAKIDQLSSDVQTLSAKVDQLSNDVNAM
RSDIQAAKDDAARANQRLDNKVSRARK
>A0A0N1R185|Salmonella schwarzengrund
MNRTKLVLGAVILGSTLLAGCSSNAKIDQLSSDVQTLNAKVDQLSNDVNAMR
SDVQAAKDDAARANQRLDNQATKYRK
>A0A3Y5ZAJ3|Salmonella senftenberg
MNRTKLVLGAVILGSTLLAGCSSNAKIDQLSSDVQTLNAKVDQLSNDVNAMR
SDVQAAKDDAARANQRLDNQATKYRK
>A0A403SH10|Salmonella thompson
MNRTKLVLGAVILGSTLLAGCSSNAKIDQLSSDVQTLNAKVDQLSNDVNAMR
SDVQAAKDDAARANQRLDNQATKYRK
>A0A3U8L4B3|Salmonella typhi
MNRTKLVLGAVILGSTLLAGCSSNAKIDQLSSDVQTLNAKVDQLSNDVNAMR
SDVQAAKDDAARANQRLDNQATKYRK
>A0A0F7J8A1|Salmonella typhimurium
MNRTKLVLGAVILGSTLLAGCSSNAKIDQLSSDVQTLNAKVDQLSNDVNAMR
SDVQAAKDDAARANQRLDNQATKYRK
>A0A735MZ47|Salmonella typhisuis
MNRTKLVLGAVILGSTLLAGCSSNAKIDQLSSDVQTLNAKVDQLSNDVNAMR
SDVQAAKDDAARANQRLDNQATKYRK
>A0A3V4B5B5|Salmonella virchow
MNRTKLVLGAVILGSTLLAGCSSNAKIDQLSSDVQTLNAKVDQLSNDVNAMR
SDVQAAKDDAARANQRLDNQATKYRK

>A0A4R3VP56| Samsonia erythrinae
MNRTKLVLGAVILGSTLLAGCSSNAKIDQLSSDVQTLNAKVDQLSNDVNAIRS
DVQAAKDDAARANQRLDNQVRTYKK
>A0A4R6EMD0| Scandinavium goeteborgense
MNRTKLVLGAVILASTMLAGCSSNAKIDQLSSDVQTLNAKVDQLSNDVNAM
RSDVQAAKDDAARANQRLDNQAHSYRK
>A0A240BYC5|Serratia ficaria
MNRTKLVLGAVILASTMLAGCSSNAKIDQLSSDVQTLNAKVDQLSNDVNAM
RSDVQAAKDDAARANQRLDNQAHAYKK
>A0A0F7HFV5|Serratia fonticola
MNRTKLVLGAVILASTMLAGCSSNAKIDQLSSDVQTLNAKVDQLSNDVNAIR
SDVQAAKDDAARANQRLDNQAHAYKK
>A0A7G2JMG3|Serratia grimesii
MNRTKLVLGAVILGSTLLAGCSSNAKIDQLSSDVQTLNAKVDQLSNDVNAIRS
DVQAAKDDAARANQRLDNQAHAYKK
>A0A379ZC49|Serratia liquefaciens
MNRTKLVLGAVILGSTLLAGCSSNAKIDQLSSDVQTLNAKVDQLSNDVNAIRS
DVQAAKDDAARANQRLDNQAHAYKK
>A0A080UXV1|Serratia marcescens
MNRTKLVLGAVILGSTLLAGCSSNAKIDQLSSDVQTLNAKVDQLSNDVNAMR
SDVQAAKDDAARANQRLDNQAHAYKK
>A0A5C7D7D4|Serratia nematodiphila
MNRTKLVLGAVILGSTLLAGCSSNAKIDQLSSDVQTLNAKVDQLSNDVNAMR
SDVQAAKDDAARANQRLDNQAHAYKK
>D4E6Y5|Serratia odorifera
MNRTKLVLGAVILGSTLLAGCSSNAKIDQLSSDVQTLNAKVDQLSNDVNAIRS
DVQAAKDDAARANQRLDNQAHAYKK
>A0A1S8CQL7|Serratia oryzae
MNRTKLVLGAVILASTMLAGCSSNAKIDQLSSDVQTLNAKVDQLSNDVNAIR
SDVQAAKDDAARANQRLDNQAHAYKK
>A0A2X4UVY1|Serratia plymuthica
MNRTKLVLGAVILGSTLLAGCSSNAKIDQLSSDVQTLNAKVDQLSNDVNAIRS
DVQAAKDDAARANQRLDNQAHAYKK
>A0A1W5DJ79|Serratia proteamaculans
MNRTKLVLGAVILASTMLAGCSSNAKIDQLSSDVQTLNAKVDQLSNDVNAIR
SDVQAAKDDAARANQRLDNQAHAYKK
>A0A2X2H9R6|Serratia quinivorans
MNRTKLVLGAVILASTMLAGCSSNAKIDQLSSDVQTLNAKVDQLSNDVNAIR
SDVQAAKDDAARANQRLDNQAHAYKK
>A0A2X5BR34|Serratia rubidaea
MNRTKLVLGAVILGSTLLAGCSSNAKIDQLSSDVQTLNAKVDQLSNDVNSIRS
DVQAAKDDAARANQRLDNQAHSYKK

>A0A1B3FBI2|Serratia surfactantfaciens
MNRTKLVLGAVILGSTLLAGCSSNAKIDQLSSDVQTLNAKVDQLSNDVNAMR
SDVQAAKDDAARANQRLDNQAHAYKK
>A0A068Z3B8|Serratia symbiotica
MNRTKLVLGSVILGSTLLAGCSSNTKIEQLSTDVQTLNAKVDQLSNDVTAIRS
DVQAAKDDAARANQRLDNQVHTYKK
>A0A5C7BQJ3|Serratia ureilytica
MNRTKLVLGAVILGSTLLAGCSSNAKIDQLSSDVQTLNAKVDQLSNDVNAMR
SDVQAAKDDAARANQRLDNQAHAYKK
>A0A6G9QN63|Shewanella aestuarii
MNKKVLLIAGVAMTALIGGCANTTALEESVANLGNKVDQLSAEVGSLKSEQS
KLSADVKGAKAASMDAQAEAKRANDRLDNMASSYKK
>A0A1S2TKI3|Shewanella algae
MNKKVLMIAGVAMTALLGGCANTTALEESVANLGNKVDQLSAEVSSLKAEQ
GKLSADVKDAKAAAMDAQSEAKRANDRLDNVASRYKK
>A1S3T4|Shewanella amazonensis
MKKVLLIAGVAMTALLGGCANTTALEESVANLSNKVDQLSADVSALKSEQG
QIAADAKAAKAAAMDAQAEAKRANDRLDNVASSYKK
>A0A431WGY1|Shewanella atlantica
MNKKVLMIAGVAMTALLGGCANTTALEESVANLGNKVDQLSSEVSSLKSEQ
GALSADVKDAKAAAMDAQAEAKRANDRLDNVASSYKK
>A0A161WSI2|Shewanella baltica
MNKKVLMIAGLAMTALLGGCANTTALEESVATLGNKVDQLSADVGSLKSEQ
SKLSADVKDAKAASMDAQAEAKRANDRLDNVASRYKK
>A0A330M259|Shewanella benthica
MNKKVLMIAGVAMTALLGGCANTTALEESISNLGNKVDQLSSEVSSLKSEQG
ALSADVKDAKAAAMDAQAEAKRANDRIDNVASSYKK
>A0A220UJF9|Shewanella bicestrii
MNKKVLMIAGLAMTALLGGCANTTALEESVATLGNKVDQLSADVSSLKSEQS
KLSADVKDAKAAAMDAQAEAKRANDRLDNVASRYKK
>A0A431WTM6|Shewanella canadensis
MNKKVLMIAGVAMTALLGGCANTTALEESVANLGNKVDQLSSEVSSLKSEQ
GALSANVKDAKAAAMDAQAEAKRANDRLDNVASSYKK
>A0A6G7LUX1|Shewanella chilikensis
MNKKVLMIAGVAMTALLGGCANTTALEESVANLGNKVDQLSAEVSSLKAEQ
GKLSADVKDAKAAAMDAQSEAKRANDRLDNVASRYKK
>A0A1E5IRW4|Shewanella colwelliana
MNKKVLMIAGVAMTALLGGCANTTALEESVANLGNKVDQLSAEVSSLKSEQ
GALSADVKDAKAAAMDAQAEAKRANDRIDNIASSYKK
>A0A5B8QZJ8|Shewanella decolorationis
MNKKVLMIAGLAMTALLGGCANTTALEESVATLGNKVDQLSADVSSLKSEQS
KLSADVKDAKAAAMDAQAEAKRANDRLDNVASRYKK

>Q12KC3|Shewanella denitrificans
MNKKVLMIAGVAMTALLGGCANTTALEESVANLGNKVDQLSAEVGSLKSEQ
SKLAVDVKGAKAAMDAQAEAKRANDRLDNMASSYKK
>A0A4R2FK90|Shewanella fodinae
MNKKVLMIAGVAMTALLGGCANTTALEESVANLGNKVDQLSAEVSAMKAD
QSKLSADVKDAKAAMDAQAEAKRANDRLDNVATRYKK
>A0A106C353|Shewanella frigidimarina
MNKKVLMIAGVAMTALLGGCANTTALEESVANLGNKVDQLSAQVGSLKSEQ
SKLSADVKGAKAASMDAQAEAKRANDRLDNMASSYKK
>B0TIP5|Shewanella halifaxensis
MNKKVLMIAGVAMTALLGGCANTTALEESVANLGNKVDQLSAEVSSLKSDH
AKMSADINDATAAALVGVAEAERANARIDNIATSYKK
>A0A553JJ59|Shewanella hanedai
MNKKVLMIAGVAMTALLGGCANTTALEESVANLGNKVDQLSSEVSSLKSEQ
GALSANVKDAKAAAMDAQAEAQRANARIDNVASSYKK
>A0A6L7HUZ1|Shewanella insulae
MNKKVLMIAGVAMTALLGGCANTTALEESVANLGNKVDQLSAEVSSLKSEQ
GALAADVKDAKAAAMDAQAEAKRANDRIDNIASSYKK
>A0A3S9L1M6|Shewanella khirikhana
MKKVLLIAGVAMTALLGGCANTTALEESVANLSNKVDQLSADVSALKSEQG
QIAADAKAAKAAAMDAQAEAKRANDRLDNVASSYKK
>A0A3G8LY20|Shewanella livingstonensis
MNKKVLMIAGVAMTALLGGCANTTALEESVANLGNKVDQLSAQVGSLKSEQ
SKLSADVKGAKAASMDAQAEAKRANDRLDNMASSYKK
>A3QBN0|Shewanella loihica
MNKKVLMIAGVAMTALLGGCANTTALEESVANLGNKVDQLSAEVSSLKSEQ
GALAADVKDAKAAAMDAQAEAKRANDRIDNIASSYKK
>A0A094J7V9|Shewanella mangrovi
MNKKVLMIAGVALTALLGGCANTTALEESVANLGSKVDQLSAEVSALKSEQS
SLSADVKDAKAAAADAQAEAKRANDRLDNVASRYKK
>A0A4Y6J4K4|Shewanella marisflavi
MNKKVLMIAGVAMTALLGGCANTTALEESVANLGNKVDQLSAEVSSLKSEQ
GALSADVKDAKAAAMDAQAEAKRANDRIDNIASSYKK
>A0A411PER4|Shewanella maritima
MKKTMMIAGVALTALLGGCANTTALEESVANLGNKVDQLSAEVGALKSEQS
KLSADVKGAKAAAMDAQAEAKRANDRLDNMASSYKK
>A0A1N6U1Q4|Shewanella morhuae
MNKKVLMIAGLAMTALLGGCANTTALEESVATLGNKVDQLSADVGSLKSEQ
SKLSADVKDAKAASMDAQAEAKRANDRLDNVASRYKK
>Q8EHD1|Shewanella oneidensis
MNKKVLMIAGLAMTALLGGCANTTALEESVATLGNKVDQLSADVSSLKSEQ
GKLSADVKDAKAAAMDAQAEAKRANDRLDNVASRYKK

>A8H160|Shewanella pealeana
MNKKVLMIAGVAMTALLGGCANTTALEESVANLGNKVDQLSAEVSSLKSDH
GKMSADINDAKAAAMDAQAEAQRANDRIDNVASSYKK
>B8CJF8|Shewanella piezotolerans
MNKKVLMIAGVAMTALLGGCANTTALEESVANLGNKVDQLSAEVSSLKSDH
GKMSADINDAKAAAMDAQAEAKRANDRIDNVASSYKK
>A0A4Y5YC50|Shewanella polaris
MNKKVLMIAGVAMTALLGGCANTTALEESVANLGNKVDQLSAQVGSLKSEQ
SKLSADVKGAKAASMDAQAEAKRANDRLDNMATSYKK
>A0A1S6HQ91|Shewanella psychrophila
MNKKVLMIAGVAMTALLGGCANTTALEESISNLGNKVDQLSSEVSSLKSEQG
ALSADVKDAKSAAMDAQAEAKRANDRIDNVASSYKK
>A0A366ITR2|Shewanella putrefaciens
MNKKVLMIAGLAMTALLGGCANTTALEESVATLGNKVDQLSADVGSLKSEQ
SKLSADVKDAKAASMDAQAEAKRANDRLDNVASRYKK
>E6XNN8|Shewanella putrefaciens
MNKKVLMIAGLAMTALLGGCANTTALEESVATLGNKVDQLSADVGSLKSEQ
SKLSADVKDAKAAAMDAQAEAKRANDRLDNVATRYKK
>A8FS71|Shewanella sediminis
MNKKVLMIAGVAMTALLGGCANTTALEESVANLGNKVDQLSSEVSSLKSEQ
GALSADVKDAKAAAMDAQAEAKRANDRLDNVASSYKK
>A0A658KXL3|Shewanella vesiculosa
MNKKVLMIAGVAMTALLGGCANTTALEESVANLGNKVDQLSAEVGSLKSEQ
SKLSADVKGAKAASMDAQAEAKRANDRLDNMASSYKK
>D4ZGR1|Shewanella violacea
MNKKVLMIAGVAMTALLGGCANTTALEESISNLGNKVDQLSSEVSSLKSEQG
ALSADVKDAKAAAMDAQAEAMRANSRIDNVASSYKK
>B1KHF0|Shewanella woodyi
MNKKVLMIAGVAMTALLGGCANTTALEESVANLGNKVDQLSSEVSALKSEQ
GALSADVKDAKAAAMDAQAEAQRANARIDNVASSYKK
>A0A073KS16|Shewanella xiamenensis
MNKKVLMIAGLAMTALLGGCANTTALEESVATLGNKVDQLSADVGSLKSEQ
SKLSADVKDAKAAAMDAQAEAKRANDRLDNVASRYKK
>A0A1Q8MN95|Shigella boydii
MKATKLVLGAVILGSTLLAGCSSNAKIDQLSSDVQTLNAKVDQLSNDVNAMR
SDVQAAKDDAARANQRLDNMATKYRK
>A0A2S8DHH7|Shigella dysenteriae
MKATKLVLGAVILGSTLLAGCSSNAKIDQLSSDVQTLNAKVDQLSNDVNAMR
SDVQAAKDDAARANQRLDNMATKYRK
>A0A1W2MPD9|Shigella flexneri
MKATKLVLGAVILGSTLLAGCSSNAKIDQLSSDVQTLNAKVDQLSNDVNAMR
SDVQAAKDDAARANQRLDNMATKYRK

>A0A0I1IY43|Shigella sonnei
MKATKLVLGAVILGSTLLAGCSSNAKIDQLSSDVQTLNAKVDQLSNDVNAMR
SDVQAAKDDAARANQRLDNMATKYRK
>I2B8C1| Shimwellia blattae
MNRTKLVLGAVILGSTLLAGCSSNAKIDQLSSDVQTLNAKVDQLSNDVNAMR
SDVQAAKDDAARANQRLDNMATKYRK
>A0A2P8VG37| Siccibacter turicensis
MNRTKLVLGAVILGSTLLAGCSSNAKIDQLSSDVQTLNAKVDQLSNDVNAIRS
DVQAAKDDAARANQRLDNQAHSYRK
>Q2NT13| Sodalis glossinidius
MNRTKLVLGAVILGSTLLAGCSSNAKIDQLSSDVQTLNAKVDHISNDVNALSS
DLQAAKDDAARANQRLDNQATSYRK
>W0HY69| Sodalis praecaptivus
MNRTKLVLGAVILGSTLLAGCSSNAKIDQLSSDVQTLNAKVDQISNDVNALRS
DVQAAKDDAARANQRLDNQATSYRK
>E8LLB1| Succinatimonas hippei
MIQFLEKIMTKYQAILGAAVLSATVLAGCSSSDALNAKLDAIAADVDALKAQ
QSKLANDVAFVKSDAARANERLDNLARRYKK
>A0A662ZSK8| Succinivibrio dextrinosolvens
MNKFQATLAVVALGSSVLVGCASNSALEAKIDAIAADVDALKAQQSKLANDV
AFVKSDAARANDRLDNMTRRYKK
>A0A2P5GU54| Superficieibacter electus
MNRTKLVLGAVILGSTLLAGCSSNAKIDQLSSDVQTLNAKVDQLSNDVNAIRS
DVQAAKDDAARANQRLDNQTRTYKK
>A0A1Y0LIF9|Tatumella citrea
MNRTKLVLGAVILGSTLLAGCSSNAKIDQLSSDVQTLNTKVDQLSNDVNAVRS
DVQAAKDDAARANQRLDNQAHAYRK
>A0A095UEI9|Tatumella morbirosei
MNRTKLVLGAVILGSTLLAGCSSNAKIDQLSSDVQTLNTKVDQLSNDVNAVRS
DVQAAKDDAARANQRLDNQAHAYRK
>A0A2X5PD39|Tatumella ptyseos
MNRTKLVLGAVILGSTLLAGCSSNAKIDQLSSDVQTLNTKVDQLSNDVNAVRS
DVQAAKDDAARANQRLDNQAHAYRK
>A0A3E0U518|Thalassotalea euphylliae
MNKFTLIAGVTAVLGLSGCANNSQLDQMSNQIDRLSNQVSKLSREVDNLKTR
QQQQKRKITEANELASQANERVNNMVATFKK
>A0A5R9ISY7|Thalassotalea litorea
MKNKMMTLAGMVFALGLAGCSSNEMLEKNVADLTMKVDNLSSQVDSLSSE
VADLKVQQQQATADSMAAKEMAAEANERVDNVVESYKK
>A0A4U1B971|Thalassotalea mangrovi
MKNKMMTLAGMVFALGLAGCSSNEMLEKNVADLNMKVDNLSAQVDSLSSE
VADLKVQQQQATADAMAAKEMAAEANERVDNVVESYKK

>A0A1I0B3D6| Thorsellia anophelis
MNRKIVLGAIVLASTVLAGCASNARVDAVEGQLNSINAKLDGLSAQIETAIGE
AKRANDRLDNMQKKSYTK
>C4L7B0| Tolumonas auensis
MNKYQVLLGTIALSSTLLVGCANTSKVESDVQTLTGKVDQLATEIGSIKDGQS
KLAADVADAKAEAARANARLDNLATSYKK
>A0A085AM34|Trabulsiella guamensis
MNRTKLVLGAVILGSTLLAGCSSNAKIDQLSSDVQTLNAKVDQLSNDVNAIRS
DVQAAKDDAARANQRLDNQATKYRK
>A0A0L0H217|Trabulsiella odontotermitis
MNRTKLVLGAVILGSTLLAGCSSNAKIDQLSSDVQTLNAKVDQLSNDVNAIRS
DVQAAKDDAARANQRLDNQATKYRK
>A0A1M6BGH4|Vibrio aerogenes
MNKLLFAIAASGVVFLSGCASQMDSSASAKLDKLSNQVSQLSDEVAALKSEQ
MTLNNKLDQASDAITSANKEAARANMRIDHIAQSYTK
>A0A0A1E9S6|Vibrio aestuarianus
MNKMLIAAAASSVLLLAGCASGPEEEMAKMDELTNKVSELSDQISALQSEQS
MLASKVNQAADASAAAQEEAARANERIDNIAQSYTK
>A0A7X8TNF9|Vibrio agarilyticus
MKKVLIAAAATSLFALAGCSNGYDTTVGELQNEVNSLSKQVAQLKNMTAQSE
MTSDVAAAAALSAQEEAERANQRLDGMSGSWNK
>A0A2K1CPQ7|Vibrio agarivorans
MNKTLIAAAATSVFLLAGCASSDDATTSKLNELSNQVSELSQNVQSLQSDVQ
KSGAAAMAAQEEAERANDRIDNIAQSYTK
>A0A347UV79|Vibrio alfacsensis
MNKTLIAAAAASVLLLAGCASSDDAAMANAAKLDELSNQVSQLSQDVQSLQ
SNVQAAGNAAMSAQEEAARANERIDNIAQSYTK
>A0A0H0Y4X7|Vibrio alginolyticus
MNKTLIAAAATSVLLLAGCASSDDAATANAAKLDELSNQVSQLSQDVQALQS
DVQKSGAAAMAAQEEAERANERIDNIAQSYTK
>A0A191W9R5|Vibrio anguillarum
MNKMLIAAAASSVLLLAGCASGPDDAAMTKMDQLSNQVSQLSDQISALKSE
QSTLSSKVSQAADAAAAAQEEAARANERIDNIAQSYTK
>A7K1E8|Vibrio antiquarius
MNKTLIAAAATSVLLLAGCASSDDAATANAAKLDELSNQVSQLSQDVQALQS
DVQKSGAAAMAAQEEAERANERIDNIAQSYTK
>A0A3S0Q434|Vibrio aquaticus
MNKMLIAAAASSVLLLAGCASGPDEATTAKMDELSNQVSQLSQDVQALQSE
VRKSGDAAMSAQEEAARANERIDNIAQSYTK
>A0A5P9CPW5|Vibrio aquimaris
MNKMLIAAAASSVLFLAGCASGPDDASSAKIDELSNQVSQLSQDVQALKSES
MSAREEAARANERIDNIAQSYTK

>A0A7Z2T884|Vibrio astriarenae
MNKMLIAAAASSVLLLAGCASGPDEETTAQLNELNAQVNQLSQDVSSLKSM
YAADVQKANDAAMSAQEEAARANERIDNIAQSYTK
>A0A1C3IL55|Vibrio atlanticus
MNKTLIAAAASVFILAGCSSEPEEAAMSQMDQLTNQVAELTSEVEALKGDKA
AAEMKAQEAAAAAMAAKEEADRANDRIDNIAESYTK
>U3AV12|Vibrio azureus
MNKTLIAAAATSVFLLAGCASSDDATTSKLNELSNQVSELSQNVQSLQSDVQ
KSGAAAMAAQEEAERANDRIDNIAQSYTK
>A0A1J4R555|Vibrio barjaei
MKKVLIAAAASSVLLLAGCASGPDEATTAKMDDLSNQVSQLSSDVQALKNE
VQMGNKNAMAAQEEAERANERIDNIAQSYTK
>A0A177Y2D4|Vibrio bivalvicida
MNKMLIAAAASSVLLLAGCASGPDAATTAKMDELSNQVSQLSQDVQALQSE
VRKSGDSAMAAQEEAARANERIDNIAQSYTK
>E8LXL0|Vibrio brasiliensis
MNKMLIAAAASSVLLLAGCASGPDEATTAKMDELSNQVSQLSQDVQALQSE
VRKSGDAAMAAQEEAARANERIDNIAQSYTK
>A0A193KHR2|Vibrio breoganii
MNNKFLLVAGVSSVLLLAGCASGPDEETTAKMADQDARVSQLEQDVAALKA
AHEKDAAMHDQSAMEAKEAAMAAQDEAARANERLDNIAQSYTK
>A0A0A3ETI0|Vibrio campbellii
MNKTLIAAAATSVLLLAGCASSDEAATATKLDELSNQVSQLSQDVQSLQSDV
QKSNDAAMAAQEEAARANERIDNIAQSYTK
>A0A0A6QST5|Vibrio caribbeanicus
MNKMLIAAAASSVLLLAGCASGPDAATTAKMDELSNQVSQLSQDVQALQSE
VRKSGDAAMSAQEEASRANERIDNIAQSYTK
>A0A1C3JE91|Vibrio celticus
MNKTLIAAAASVFILAGCSSEPEEAAVSEMDQLTNQVAQLTSEVEALKSDKAA
AEMKAQEAEAAAMAAKEEADRANERIDNIAESYTK
>A0A2S7VR19|Vibrio chagasii
MNKTLIAAAASVFILAGCSSEPEEAAVSEMDQLTNQVAQLTSEVEALKSDKAA
AEMKAQEASEAAMAAKEEADRANDRIDNIAESYTK
>A0A085PVQ9|Vibrio cholerae
MNKMLIAAAASSVLLLAGCASGPDEATTAKMNEISTQVSELNSQVAALASKV
DQAAEAAKAAQEEAARANERIDNIAQSYTK
>A0A151KYW2|Vibrio cidicii
MNKILIAAAATSVLLLAGCASGPDAQTKAAMSQVDQLKNQVEMLSKEVAAL
KSNQADAEMQAQNASMAAKEASMAAMAAQEEAVRANERIDNIAQSYTK
>A0A1T4Q593|Vibrio cincinnatiensis
MNKMLLAATASSVLLLAGCASGTDEAMTTKLDELNNQVSQLNEQVSALRSE
TASLTSKANTATDAAVSAQEEAARANERIDNIAQSYTK

>A0A4Y3IP19|Vibrio comitans
MNNKFLLVAGVSSVLLLAGCASGPDEETTAKMADQDARVSQLEQDVAALKA
AHEKDAAMHDQSAMEAKEAAMAAQDEAARANERLDNIAQSYTK
>A0A097B062|Vibrio coralliilyticus
MNKMLIAAAASSVLLLAGCASGPDEASSAKIDELSNQVSQLSQDVQALQSDV
RKSGDAAMSAQEEAARANERIDNIAQSYTK
>A0A0T7D2N2|Vibrio coralliirubri
MNKTLIAAAASVFILAGCSSEPEEAAVSEMDQLTNQVAQLTSEVEALKSDKAA
AEMKAQEAEAAAMAAKEEADRANDRIDNIAESYTK
>A0A0T7DJ57|Vibrio crassostreae
MNKTLIAAAASVFILAGCSSEPEEAAVSEMDQLTNQVAQLTSEVEALKSDKAA
AEMKAQEAEAAAMAAKEEADRANERIDNIAESYTK
>A0A7Z1MEM9|Vibrio cyclitrophicus
MNKTLIAAAASVFILAGCSSEPEEAAMSEMDQLTNQVAELSSEVEALKSDKA
AAEMKAQEASAAAMAAKEEADRANDRIDNIAESYTK
>A0A0T7E418|Vibrio diabolicus
MNKTLIAAAATSVLLLAGCASSDDAATANAAKLDELSNQVSQLSQDVQALQS
DVQKSGAAAMAAQEEAERANERIDNIAQSYTK
>A0A2J8GXY6|Vibrio diazotrophicus
MNKMLIAAAASSVLLLAGCASGPDEATTAKLDELNSQVSQLNDEVAALKSEH
SALASKANAASDSAMAAQEEAARANERIDNIAQSYTK
>A0A178J513|Vibrio europaeus
MNKMLIAAAASSVLLLAGCSSTPDEATTAKIDELTNQVSQLSQDVQALQSEVR
KSGDSAMAAQEEAARANERIDNIAQSYTK
>U3CEK8|Vibrio ezurae
MNKKIILVAGVSSVLLLAGCASGPDDATKAQMADTDSRVSQLEKDLAALKAE
HEKDSAENKATAMQAKEAAMAAQDESARANERLDNVAQSYTK
>A0A0X8LJP1|Vibrio fluvialis
MNKMLIAAAASSVLLLAGCASGPDEATTMKLDELSNQVSQLSDEVASLKSDH
AAVMSKLNATSDAAMAAQEEAARANERIDNIAQSYTK
>A0A066UT84|Vibrio fortis
MNKVLIAAAASVFVLAGCSSDPEEAAMSEVEQLSNQVAQLSSEVEALKSEKA
DAEMKAQEATDAAMAAKEEAMRANERIDNIANSYTK
>A0A0Q2V4S7|Vibrio furnissii
MNKMLIAAAASSVLLLAGCASGPDEATTAKLDELNNQVSQLSDQVASLKSDH
AAMMSKANATSDAAMAAQEEAARANERIDNIAQSYTK
>A0A0F4NNX8|Vibrio galatheae
MNKMLIAAAASSVLLLAGCASGPDEATTAKMDELSNQVSQLSQDVQALQSE
VRKSGDSAMAAQEEAARANERIDNIAQSYTK
>A0A1Z2SM51|Vibrio gazogenes
MNKVFLAVAASGVVFLSGCASSTDSATTAKMDELSNQVSQLSDQVASLKSSQ
MMMSDKIDQTSDSVNAVREEAQRANERIDHIAQSYTK

>A0A5M9P5E8|Vibrio gigantis
MNKTLIAAAASVFILAGCSSEPEEAAVSEIDQLTNQVAQLTSEVEALKSDKAA
AEMKAQEAEAAAMAAKEEADRANERIDNIAESYTK
>V5FJK9|Vibrio halioticoli
MNKKIILAAGVSSVLLLAGCASGPDEATKAQMADTDSRVSQLEQDLAALKAE
HEKDSAENKAAAMQAKEAAMAAQDESARANERLDNVAQSYTK
>A0A1H5VEA4|Vibrio hangzhouensis
MKKVLIAAAASSVLLLAGCASGPDEATTAKLDDLSNQVSQLSADVQSLKGEV
QESNKNAMAAQEEAARANERIDNIAQSYTK
>A0A0D0J630|Vibrio harveyi
MNKTLIAAAATSVLLLAGCASSDEAATATKLDELSNQVSQLSQDVQSLQSDV
QKSSDAAMAAQEEAARANERIDNIAQSYTK
>A0A0M0I0T3|Vibrio hepatarius
MNKMLIAAAASSVLLLAGCASGPDEETTAKMDELSNQVSQLSQDVQALQSD
VRKSGDAAMAAQEEAARANERIDNIAQSYTK
>F9RX44|Vibrio ichthyoenteri
MNKMIIAAAASSVLLLAGCASGPDEATTAKLDELSNQVSQLSQDVNALAGGV
QASGDAAMAAQEEAARANERIDNIAQSYTK
>A0A4Y3HVD2|Vibrio inusitatus
MNNKFLLIAGVSSVLLLAGCASGPDEETTAKMADQDARVSQLEQDVAALKA
AHENDADSAMEAKQAKEAAMAAQDEAARANERLDNIAQSYTK
>A0A0B8P8Y0|Vibrio ishigakensis
MNNKFLLAAGVSSVLLLAGCASGPDEETTAKMNDQDARISQLESDVAALKA
AHEKDSAENKAAAMQAKEAAMAAQDEAARANERLDNVSQSYTK
>A0A2S7W7H4|Vibrio jasicida
MNKTLIAAAATSVLLLAGCASSDEAATATKLDELSNQVSQLSQDVQSLQSDV
QKSSDAAMAAQEEAARANERIDNIAQSYTK
>A0A2N7JDL0|Vibrio kanaloae
MNKTLIAAAASVFILAGCSSEPEEAAMSEMDQLTNQVAELSNEVEALKSDKA
AAEMKAQEASAAAMAAKEEADRANDRIDNIAESYTK
>A0A1B9Q1U1|Vibrio lentus
MNKTLIAAAASVFILAGCSSEPEEAAVSEMDQLTNQVAELTSEVEALKSDKAA
AEMKAQEASAAAMAAKEEADRANDRIDNIAESYTK
>A0A1Y6IVV0|Vibrio mangrovi
MNKVFLAVAASGVVFLSGCASSTDSATMAKMDELSNQVSQLSDEVATLKNG
QMMLSDKIDQNSESISAVREEAQRANERIDHIAQSYTK
>A0A7W2FNN6|Vibrio marinisediminis
MNKMFIAAAATSVLLLAGCASGPDEATTAKLDELSNQVSQLSQDVQALNSDV
QKSGSAAMAAQEEAARANERIDNIAQSYTK
>A0A090RSV1|Vibrio maritimus
MKKVLIAAAASSVLLLAGCASGPDEATTAKMDDLSNQVSQLSADVQALKNE
VQMGNKNAMAAQEEAERANERIDNIAQSYTK

>A0A241TDR8|IPR016367|Vibrio mediterranei
MKKVLIAAAASSVLLLAGCASGPDEATTAKMDDLSNQVSQLSSDVQALKNE
VQMGNKNAMAAQEEAERANERIDNIAQSYTK
>A0A067BIY1|Vibrio metoecus
MNKMLIAAAASSVLLLAGCASGPDEATTAKMNEISNQVSELNSQVAALTSKV
DQATEAAKAAQEEAARANERIDNIAQSYTK
>A0A380Q1S7|Vibrio metschnikovii
MNKMLLAAAASSVLLLAGCASGPDEATTTKLDQLNNQVSQLNSQIAALKSE
NASLTDAALAAQEEAARANERIDNIAQSYTK
>A0A1D8SH82|Vibrio mimicus
MNKMLIAAAASSVLLLAGCASGPDEATTAKMNEISNQVSELNTQVAALTSKV
DQATEAAKAAQEEAARANERIDNIAQSYTK
>A0A0C3E6X1|Vibrio mytili
MNKTLIAAAATSVLLLAGCASSDDAVTANAAKLDELSNQVSQLSQDVQSLQS
DVQMSKDAAMSAQEEAARANERIDNIAQSYTK
>A0A1B1EIJ6|Vibrio natriegens
MNKTLIAAAATSVLLLAGCASSDDATTAKLDELSNQVSMLSQDVQTLQSDVQ
MSKDAAMSAQEEAARANERIDNIAQSYTK
>A0A099LXQ1|Vibrio navarrensis
MNKILIAAAATSVLLLAGCASGPDAQTKAAMSQVDELKNQVEMLSKEVAAL
KSNQADAEMQAQNASMAAKEASMAAMAAQEEAVRANERIDNIAQSYTK
>A0A4P8G581|Vibrio neocaledonicus
MNKTLIAAAATSVLLLAGCASSDDAATANAAKLDELSNQVSQLSQDVQALQS
DVQKSGAAAMAAQEEAERANERIDNIAQSYTK
>A0A0F4P305|Vibrio neptunius
MNKMLIAAAASSVLLLAGCASGPNDASSAKLDELSNQVSQLSQDVQALQSD
VRKSGDAAMSAQEEAARANERIDNIAQSYTK
>A0A0M0HMI2|Vibrio nereis
MNKMLIAAATSSVLLLAGCASGPDEATTAKMDELSNQVSQLSQDVQSLQSDV
RRSTDAAMSAQEEAARANERIDNIAQSYTK
>U4KEB4|Vibrio nigripulchritudo
MNNKLLLAAGISSVLFLAGCSGTPDTTSKKIDELTNQVQQLSQQVSSLQSSQE
KASMKAEEAASAANGAKNAAMSAQQEASRANSRIDNIAQSYTK
>C9QFZ8|Vibrio orientalis
MNKMLIAAAASSVLLLAGCASGPDEATTAQLDELNNQVSQLSQNVEALQAE
VSKSGDAAMAAQEEAARANERIDNIAQSYTK
>A0A4Y8WK39|Vibrio ouci
MNKMLIAAAASSVLLLAGCASGPDEATTAQLDELNNQVSQLSQNVEALQAE
VSKSGDAAMAAQEEAARANERIDNIAQSYTK
>A0A3G2Q029|Vibrio owensii
MNKTLIAAAATSVLLLAGCASSDEAATATKLDELSNQVSQLSQDVQSLQSDV
QKSNDAAMAAQEEAARANERIDNIAQSYTK

MNKTLIAAAATSVLLLAGCASSDEAATATKLDELSNQVSQLSQDVQSLQSDV
QKSNDAAMAAQEEAARANERIDNIAQSYTK
>A0A1R4B665|Vibrio palustris
MNKMLLAAATSSVLLLAGCASGTNEATTSKLDNLTDQVEQLQDEVASLKSEH
AALASKTNQSADAAMAAQDEAQRANKRIDNIAQSYTK
>A0A1Q9HR18|Vibrio panuliri
MNKMFIAAAATSVLLLAGCASGPDEATTAKLDELSNQVSQLSQDVNALKSDV
QKSGSAAMAAQEEAARANERIDNIAQSYTK
>A0A072L5U8|Vibrio parahaemolyticus
MNKTLIAAAATSVLLLAGCASSDDAATANAAKLDELSNQVSQLSQDVQSLQS
DVQKSGDAAMAAQEEAARANERIDNIAQSYTK
>A0A432D834|Vibrio penaeicida
MNSKLLLAAGISSVLFLAGCSGAPDTASSKLDELTNQVQQLSQQVSSLQSSQE
KASMKAEEAASAANGAKSAAMSAQQEAARANSRIDNIAQSYTK
>A0A7Y3IKC0|Vibrio plantisponsor
MNKMLIAAAAASSVLLLAGCASGPDEATTAKLDELNSQVSQLNDEVAALKSEH
SALASKANAASDSAMAAQEEAARANERIDNIAQSYTK
>A0A090P116|Vibrio ponticus
MNKMFIAAAATSVLLLAGCASGPDEATTAKLDELSNQVSQLSQDVNSLKSDV
NKAGGAAMAAQEEAARANERIDNIAQSYTK
>U3BEX2|Vibrio proteolyticus
MNKMLIAAAAASSVLLLAGCASGPDEATTAKMDELSNQVSQLSQDVQSLKSEV
QKTGAAAQSAQEEAARANERIDNIAQSYTK
>A0A223N3Y5|Vibrio qinghaiensis
MNKMLIAATASSILLLAGCASGPDGVEMTKMDQLSNQVSQLSDQISALKSEQ
STLSSKVSQAADAATAAQEEAARANERIDNIAQSYTK
>A0A1M7YPP6|Vibrio quintilis
MNKLLFAIAASGVVFLSGCASQMDSSASAKLDKLSSQVSQLSDEVAALKSEQ
MTLNNKLDQTSDAITSANKEAARANMRIDHIAQSYTK
>A0A0C2K2R6|Vibrio renipiscarius
MNKIIIAAAAASSVLLLAGCASGPDEATTAKLDELSNQVSQLSQDVNALAGGV
QASGDAAMAAQEEAARANERIDNIAQSYTK
>A0A2K7T029|Vibrio rotiferianus
MNKTLIAAAATSVLLLAGCASSDDAATANAAKLDELSNQVSQLSQDVQALQS
NVQKSGEAAMAAQEEAARANERIDNIAQSYTK
>A0A1R4LQZ5|Vibrio ruber
MNKVFLAVAASGVVFLSGCASSTDSATTAKMDELSNQVSQLSDQVASLKSSQ
MMLSDKIDQTSDSVNAVREEAQRANERIDHIAQSYTK
>A0A511QH73|Vibrio sagamiensis
MNKTLIAAAATSVFLLAGCASSDDATTSKLNELSNQVSELSQNVQSLQSDVQ
KSGAAAMAAQEEAERANDRIDNIAQSYTK
>A0A1B1NWE6|Vibrio scophthalmi

MNKMIIAAAASSVLLLAGCASGPDEATTAKLDELSNQVSQLSQDVNALAGGV
QASGDAAMAAQEEAARANERIDNIAQSYTK
>A6CVF6|Vibrio shilonii
MKKVLIAAAASSVLLLAGCASGPDEATTAKMDDLSNQVSQLSSDVQALKNE
VQMGNKNAMAAQEEAERANERIDNIAQSYTK
>E8M375|Vibrio sinaloensis
MNKMLIAAAASSVLLLAGCASGPDEAATAKMDELNNQISQLSQDVQALQSE
VRKSGDAAMSAQEEAARANERIDNIAQSYTK
>A0A3A6R0G1|Vibrio sinensis
MNKMLIAAAASSVLLLAGCASSEDATTAKLDELSNQVSQLSQDVQGLQSDVQ
QSNSAAMAAQEEAARANERIDNIAQSYTK
>A0A1N6M934|Vibrio spartinae
MNKVFLAVAASGVVFLSGCASSTDSATTAKMDELSNQVSQLSDQVASLKSSQ
MMMSDKIDQTSDSVNAVREEAQRANERIDHIAQSYTK
>A0A0P6ZDD5|Vibrio splendidus
MNKTLIAAAASVFILAGCSSEPEEAAVSEMDQLTNQVAELSSEVEALKSDKAA
AEMKAQEASAAAMAAKEEADRANDRIDNIAESYTK
>A0A511QPX5|Vibrio superstes
MNNKFLLIAGVSSVLLLAGCASGPDEETTAKMADQDARVSQLEQDVAALKA
AHEKDAAMHDQSAMEAKEAAMAAQEEAARANERLDNIAQSYTK
>A0A0A1E782|Vibrio tapetis
MNKKLILAAGVSSVVFLAGCSGSPDSTEMKIDKLTNQVEQLSQQVQTLQTSQ
TTATMEAKEAASSAMSAKDAAMSAQQEASRANDRIDNIAQSYTK
>A0A1A6L4A9|Vibrio tasmaniensis
MNKTLIAAAASVFILAGCSSEPEEAAVSEMDQLTNQVAELTSEVEALKSDKAA
AEMKAQEASAAAMAAKEEADRANDRIDNIAESYTK
>A0A240EF83|Vibrio thalassae
MKKVLIAAAASSVLLLAGCASGPDEATTAKMDDLSNQVSQLSADVQALRSE
VLIGNKNAMAAQEEAERANERIDNIAQSYTK
>A0A109D6H3|Vibrio toranzoniae
MNKTLIAAAASVFILAGCSSEPEEAAMSEMDLLTNQVAELSNEVESLKNDKA
AAEMKAQQAEAAAMAAKEEADRANDRIDNIAESYTK
>F9T4Z4|Vibrio tubiashii
MNKMLIAAAASSVLLLAGCASGPDEATTAKMDELSNQVSQLSQDVQALQSE
VRKSGDSAMAAQEEAARANERIDNIAQSYTK
>A0A090SHI9|Vibrio variabilis
MKKVLIAAAASSVLLLAGCASGPDEATTAKMDDLSNQVSQLSADVQALKNE
VQMGNKNAMAAQEEAERANERIDNIAQSYTK
>A0A3N9TKK1|Vibrio viridaestus
MNKLFLVAASSALILSGCASSTDEATTSKLDELSNKVDMLSEKISSMEASHGQ
MTEKLNQTADAASAAQEEAQRANERIDNIAQSYTK
>A0A087IRR1|Vibrio vulnificus

MNKILIAAATSSVLLLAGCASGPDEQTKAAMAQVEDLKNQVDVLTKEVAAL
KSNQADAEMKAKEAATAAMAAQEEAERANERIDNIAQSYTK
>A0A1G8AFV7|Vibrio xiamenensis
MNKMLIAAAASSVLLLAGCATGPDAETTAQMDELSNQVSQLSQEVSSMKSEL
TAEVQKSSDAAMAAQEEAARANERIDNIAQSYTK
>Q8D2J9| Wigglesworthia glossinidia
MFNKKIIFITTFGLFLLSSCAKNPNMNKISYDVQDLKYKINQISSDINHIQSDVN
KAKEEAERANNRMDNNTRYYRK
>A0A1Y2SRD2|Xenorhabdus beddingii
MNRTKVVLGAVILASTLLAGCSSATKVEQLASQVQTLGSKVDQLSNDLSSIRS
DVQSAKDEAARANQRLNNQVRSYKK
>A0A0B6XDK5|Xenorhabdus bovienii
MNRTKVVFGAVILASTLLAGCSSATKVEQLATQVNTLGSKVDQLRNDLSAVH
SDVQSAKEEAARANKRLDNQVRSYKK
>A0A2D0J3X9|Xenorhabdus budapestensis
MNRTKVVLGAVILASTMLAGCTSPPKVDQLSSQVQTLGSKVDQLSNEVSSMH
SDVQTARDEAARANQRLDNQVRSYKK
>W1IMQ8|Xenorhabdus cabanillasii
MNRTKVVLGAVILASTMLAGCTSTTKVDQLSSQVQTLGSKVDQLSNEVSSVR
SDVQTARDEAARANQRLDNQVRSYKK
>A0A068QRD4|Xenorhabdus doucetiae
MNRTKVVFGAVILASTLLAGCSSATKVEQLASQVQTLGSKVDQLSSDISSIRSD
VQSARDEAARANQRLDNQVRSYKK
>A0A1Q5TZY5|Xenorhabdus eapokensis
MNRTKVVFGAVILASTLLAGCSSATKVEQLASQVQTLGSKVDQLSSDLSSIRS
DVQSARDEAARANQRLDNQVRSYKK
>A0A2D0IP45|Xenorhabdus ehlersii
MNRTKVVFGAVILASTLLAGCSSATKVEQLASQVQTLGSKVDQLSSDLSSIRS
DVQSARDEAARANQRLDNQVRSYKK
>A0A2G0Q1U3|Xenorhabdus hominickii
MNRTKVLLGAVILASTMLAGCSSATKVEQLASQVQTLGSKVDQLSNDLSSVR
SDVQSAKDEAARANQRLNNQVKSYKK
>A0A1N6MYG9|Xenorhabdus innexi
MNRTKIVLGAVILASTMLAGCTSTAKKVEQLSSQVLTLGSKVDQLSNEVGSM
RSDVQTARDEAARANQRLDNQVRSYKK
>A0A2D0KAJ3|Xenorhabdus ishibashii
MNRTKVVFGAVILASTLLAGCSSATKVEQLASQVQTLGSKVDQLSSDLSSIRS
DVQSARDEASRANQRLDNQVRSYKK
>A0A1I4YYS7|Xenorhabdus japonica
MNRPKVVFGAVILASTLLAGCSSATKVEQLASQVQTLGSKVDRLSSDLSSIRS
DVQSAKDEATRANQRLNNQVRSYKK
>A0A0J5FQ00|Xenorhabdus khoisanae

MNRTKVVLGAVILASTLLAGCSSATKVEQLASQVQTLGSKVDQLSNDLSSIRS
DVQSAKDEAARANQRLNNQVRSYKK
>A0A1I7FAA4|Xenorhabdus koppenhoeferi
MNRTKVVFGAVILASTLLAGCSSATKVEQLASQVQTLGSKVDQLSNDLGSIRS
DVQSAKDEAARANQRLNNQVHSYKK
>A0A2D0LAL0|Xenorhabdus kozodoii
MNRTKVVFGAVILASTLLAGCSSATKVEQLASQVQTLGSKVDQLSSGLSAIRS
DVQSAKDEAARANQRLDNQVRSYKK
>A0A1I3NGH6|Xenorhabdus mauleonii
MNRTKVVFGAVILASTLLAGCSSATKVEQLASQVQTLGSKVDQLSNDLSSIRS
DVQSAKDEAARANQRLNNQVRSYKK
>A0A2D0JM80|Xenorhabdus miraniensis
MNRTKVVLGAVILASTLLAGCSSATKVEQLASQVQTLGSKVDQLSNDLSSIRS
DVQSAKDEAARANQRLNNQVRSYKK
>A0A7G8K4S4|Xenorhabdus nematophila
MNRTKVLLGAVILASTLLAGCSSATKVEQLASQVQTLGSKVDQLSNDLSSVRS
DVQSAKDEAARANQRLNNQVRSYKK
>A0A068R1W9|Xenorhabdus poinarii
MNRTKVVFGAVILASTLLAGCSSATKVEQLASQVQTLGSKVDQLSSDLNSVRS
DVQSAKDDAARANQRLNNQVRSYKK
>A0A2D0KUE3|Xenorhabdus stockiae
MNRTKIVLGAVILASTMLAGCTSTAKKVDQLSSQVLTLGSKVDQLSNEVGSM
RSDVQTAKDEAARANQRLDNQVRSYKK
>W1IQW7|Xenorhabdus szentirmaii
MNRTKVVFGAVILASTLLAGCSSATKVEQLASQVQTLGSKIDQLNNDLSSVRS
DVQTAKDEAARANQRLNNQVRSYKK
>A0A1Q5U523|Xenorhabdus thuongxuanensis
MNRTKVVFGAVILASTLLAGCSSATKVEQLASQVQTLGSKVDQLSSDLSSIRS
DVQSARDEAARANQRLDNQVRSYKK
>A0A1Y2SDF5|Xenorhabdus vietnamensis
MNRTKVVFGAVILASTLLAGCSNATKVEQLASQVHTLGSKVDRLSSDISSIRSE
AQSAKDEAARANQRLNNQVRSYKK
>A0A0T9T6Y6| Yersinia aldovae
MNRTKLVLGAVILASTMLAGCSSNAKIDQLSSDVQTLNAKVDQLSNDVNAIR
SDVQAAKDDAARANQRLDNQAHAYKK
>A0A0T9UUB7| Yersinia aleksiciae
MNRTKLVLGAVILASTMLAGCSSNAKIDQLSSDVQTLNAKVDQLSNDVNAIR
SDVQAAKDDAARANQRLDNQAHAYKK
>A0A2G4TZP8| Yersinia bercovieri
MNRTKLVLGAVILASTMLAGCSSNAKIDQLSSDVQTLNAKVDQLSNDVNAIR
SDVQAAKDDAARANQRLDNQAHAYKK
>A0A0E1NGB2| Yersinia enterocolitica

MNRTKLVLGAVILASTMLAGCSSNAKIDQLSSDVQTLNAKVDQLSNDVNAIR
SDVQAAKDDAARANQRLDNQAHAYKK
>A0A3S6EY62| Yersinia entomophaga
MNRTKLVLGAVILASTMLAGCSSNAKIDQLSSDVQTLNAKVDQLSNDVNAIR
SDVQAAKDDAARANQRLDNQAHAYKK
>A0A0T9TXG7| Yersinia frederiksenii
MNRTKLVLGAVILASTMLAGCSSNAKIDQLSSDVQTLNAKVDQLSNDVNAIR
SDVQAAKDDAARANQRLDNQAHAYKK
>A0A481QDG2| Yersinia hibernica
MNRTKLVLGAVILASTMLAGCSSNAKIDQLSSDVQTLNAKVDQLSNDVNAIR
SDVQAAKDDAARANQRLDNQAHAYKK
>A0A0B6I138| Yersinia intermedia
MNRTKLVLGAVILASTMLAGCSSNAKIDQLSSDVQTLNAKVDQLSNDVNAIR
SDVQAAKDDAARANQRLDNQAHAYKK
>A0A0T9KQL2| Yersinia kristensenii
MNRTKLVLGAVILASTMLAGCSSNAKIDQLSSDVQTLNAKVDQLSNDVNAIR
SDVQAAKDDAARANQRLDNQAHAYKK
>A0A2R4NQA0| Yersinia massiliensis
MNRTKLVLGAVILASTMLAGCSSNAKIDQLSSDVQTLNAKVDQLSNDVNAIR
SDVQAAKDDAARANQRLDNQAHAYKK
>A0A0U1I073| Yersinia mollaretii
MNRTKLVLGAVILASTMLAGCSSNAKIDQLSSDVQTLNAKVDQLSNDVNAIR
SDVQAAKDDAARANQRLDNQAHAYKK
>A0A0T9LFU7| Yersinia nurmii
MNRTKLVLGAVILASTMLAGCSSNAKIDQLSSDVQTLNAKVDQLSNDVNAIR
SDVQAAKDDAARANQRLDNQAHAYKK
>A0A0T9QTS5| Yersinia pekkanenii
MNRTKLVLGAVILASTMLAGCSSNAKIDQLSSDVQTLNAKVDQLSNDVNAIR
SDVQAAKDDAARANQRLDNQAHAYKK
>A0A6B3T5S4| Yersinia pestis
MNRTKLVLGAVILASTMLAGCSSNAKIDQLSSDVQTLNAKVDQLSNDVNAVR
ADVQAAKDDAARANQRLDNQAQAYKK
>A0A0T9JEB5| Yersinia pseudotuberculosis
MNRTKLVLGAVILASTMLAGCSSNAKIDQLSSDVQTLNAKVDQLSNDVNAVR
SDVQAAKDDAARANQRLDNQAQAYKK
>A0A0U1HPA2| Yersinia rohdei
MNRTKLVLGAVILASTMLAGCSSNAKIDQLSSDVQTLNAKVDQLSNDVNAIR
SDVQAAKDDAARANQRLDNQAHAYKK
>A0A085U7W2| Yersinia ruckeri
MNRTKLVLGAVILASTMLAGCSSNAKIDQLSSDVQTLNAKVDQLSNDVNAIR
SDVQAAKDDAARANQRLDNQAHAYKK
>A0A0T9QHT6| Yersinia similis

MNRTKLVLGAVILASTMLAGCSSNAKIDQLSSDVQTLNAKVDQLSNDVNAVR
SDVQAAKDDAARANQRLDNQAQAYKK
>A0A0E8XK02| Yersinia wautersii
MNRTKLVLGAVILASTMLAGCSSNAKIDQLSSDVQTLNAKVDQLSNDVNAVR
SDVQAAKDDAARANQRLDNQAQAYKK
>A0A6H0K6P9| Yokenella regensburgei
MNRTKLVLGAVILGSTLLAGCSSNAKIDQLSSDVQTLNAKVDQLSNDVNAMR
SDVQAAKDDAARANQRLDNQATKYRK
>A0A231N1U4| Zobellella denitrificans
MRNKMMLVGGVVSALLLAGCSSTTALESKLDSLAQDVQAVQQGQQMNSAKI
DRLAADVAEARASADRANSRLDQMGRYTK
>A0A2P7RD43| Zobellella endophytica
MRNKMMLVGGVVSALLLAGCSSTTALESKLDSLAQDVQAVQQGQQMNSAKI
DRLAADVAEARASADRANSRLDQMGRYTK
>A0A2P7QEN4| Zobellella taiwanensis
MRNKMMLVGGVVSALLLAGCSSTTALESKLDSLAQDVQAVQQGQQANSAKI
DRLAADVAEARASADRANSRLDQMGRYTK
